# Targeting *Lactobacillus johnsonii* to reverse chronic kidney disease

**DOI:** 10.1038/s41392-024-01913-1

**Published:** 2024-08-05

**Authors:** Hua Miao, Fei Liu, Yan-Ni Wang, Xiao-Yong Yu, Shougang Zhuang, Yan Guo, Nosratola D. Vaziri, Shi-Xing Ma, Wei Su, You-Quan Shang, Ming Gao, Jin-Hua Zhang, Li Zhang, Ying-Yong Zhao, Gang Cao

**Affiliations:** 1 School of Pharmacy, Zhejiang Chinese Medical University, Hangzhou, Zhejiang China; 2 State Key Laboratory of Quality Research in Chinese Medicines, Macau University of Science and Technology, Macau, China; 3 State Key Laboratory of Kidney Diseases, First Medical Center of Chinese PLA General Hospital, Beijing, China; 4 Department of Urology, Cancer Hospital, Chinese Academy of Medical Sciences and Peking Union Medical College, Beijing, China; 5 Department of Nephrology, Shaanxi Traditional Chinese Medicine Hospital, Xi’an, Shaanxi China; 6 Department of Medicine, Rhode Island Hospital and Alpert Medical School, Brown University, Providence, RI USA; 7 Department of Public Health and Sciences, University of Miami, Miami, FL USA; 8 School of Medicine, University of California Irvine, Irvine, CA USA; 9 Department of Nephrology, Baoji Central Hospital, Baoji, Shaanxi China; 10 Department of Nephrology, Xi’an Peoples Hospital, Xi’an, Shaanxi China

**Keywords:** Kidney diseases, Systems biology

## Abstract

Accumulated evidence suggested that gut microbial dysbiosis interplayed with progressive chronic kidney disease (CKD). However, no available therapy is effective in suppressing progressive CKD. Here, using microbiomics in 480 participants including healthy controls and patients with stage 1–5 CKD, we identified an elongation taxonomic chain Bacilli-Lactobacillales-Lactobacillaceae-*Lactobacillus*-*Lactobacillus johnsonii* correlated with patients with CKD progression, whose abundance strongly correlated with clinical kidney markers. *L. johnsonii* abundance reduced with progressive CKD in rats with adenine-induced CKD. *L. johnsonii* supplementation ameliorated kidney lesion. Serum indole-3-aldehyde (IAld), whose level strongly negatively correlated with creatinine level in CKD rats, decreased in serum of rats induced using unilateral ureteral obstruction (UUO) and 5/6 nephrectomy (NX) as well as late CKD patients. Treatment with IAld dampened kidney lesion through suppressing aryl hydrocarbon receptor (AHR) signal in rats with CKD or UUO, and in cultured 1-hydroxypyrene-induced HK-2 cells. Renoprotective effect of IAld was partially diminished in AHR deficiency mice and HK-2 cells. Our further data showed that treatment with *L. johnsonii* attenuated kidney lesion by suppressing AHR signal via increasing serum IAld level. Taken together, targeting *L. johnsonii* might reverse patients with CKD. This study provides a deeper understanding of how microbial-produced tryptophan metabolism affects host disease and discovers potential pathways for prophylactic and therapeutic treatments for CKD patients.

## Introduction

Chronic kidney disease (CKD) is a global progressive refractory disease with high prevalence and mortality that extensive affects 15–20% adult population, especially in patients with hypertension and diabetes.^1^ Numerous proportion patients ultimately progress to end-stage kidney disease that needs dialysis or kidney transplantation. Regardless of the underlying etiology, renal fibrosis is the finally and common outcomes and pathological characteristics of progression of CKD.^2^ Mounting evidence has highlighted that a verity of factors that were implicated in progressive renal fibrosis included classical risk factors, microvascular damage, inflammation and metabolic alterations that were associated with hyperactive renin-angiotensin system, aryl hydrocarbon receptor (AHR), Wnt/β-catenin and transforming growth factor β/Smad signaling pathways as well as the endogenous metabolite disorder and microbial dysbiosis.^2–4^ Although CKD and its complications are associated with dietary, tobacco use, obesity, high blood pressure, ageing and diabetes mellitus,^5,6^ these classical risk factors cannot totally explain clinical outcomes of renal disease. With development of high throughout metabolomics techniques, a myriad of known and novel host-produced metabolites such as indoxyl sulfate, p-cresol glucuronide and p-cresol sulfate are identified in CKD patients.^4,7^ The retention of endogenous metabolites especially uremic toxins is one of the important characteristics of CKD and its complications.^4^ In the last two decades, several seminal reviews have displayed a variety of host-produced uremic toxins that contribute to CKD.^8^ In the recent several years, metagenomic technology has demonstrated microbial dysbiosis in CKD patients,^9,10^ suggesting that microbial dysbiosis and the disorder of microbial-derived metabolites had an important effect on CKD patients.

Metabolic regulation is a mainly physiological functions in gut microbiota. Microbial-produced metabolites were demonstrated to be as important factors for host health. The microbial dysbiosis led to metabolite disorder, which were involved in CKD.^8,11^ Significant alterations in gut microbial structure accompanied by the disturbance of serum metabolite levels in CKD patients indicated gut microbiome-based metabolome disorder in CKD.^10^ Several seminal reviews highlighted a significant increase in the abundance of Fusobacteria and Proteobacteria as well as *Streptococcus*, *Escherichia_Shigella* and *Desulfovibrio*, while a significant decrease in the abundance of *Faecalibacterium*, *Prevotella_9* and *Roseburia* in patients with CKD; and a significant increase in the abundance of Proteobacteria and *Fusobacterium* and *Streptococcus*, while a significant decrease in the abundance of *Faecalibacterium*, *Coprococcus* and *Prevotella* in patients with end-stage kidney disease,^3,12,13^ which were accompanied by higher levels of indoxyl sulfate and p-cresyl sulfate as well as lower levels of short chain fatty acids.^14,15^ The microbial dysbiosis was also demonstrated in various renal diseases such as diabetic kidney disease and membranous nephropathy.^3,13^ Substantial evidence also dictated a critical link between alteration in gut microbiota and outcomes of patients undergoing hemodialysis, peritoneal dialysis and kidney transplantation.^16,17^ Therefore, targeting microbial dysbiosis is a critical therapy for treatment management of patients with CKD.

Although combined metagenome and metabolome revealed links between dysregulation of microbial-produced metabolites in host (serum) and dysbiosis of gut microbiota in many diseases such as obesity, hypertension and acute liver injury, indicating that microbial-produced metabolites contributed to disease development and progression,^18^ the identification of microbial-produced metabolites and deeper understanding of its molecular mechanism in CKD is still in its infancy compared with above-mentioned diseases. A few microbial-produced metabolites such as phenyl sulfate in CKD have been identified by combined metagenomics and metabolomics.^19^ AHR is a typical low-molecular-weight metabolites-activated transcription factor that transcribes downstream target genes, such as cytochrome P450 family 1 subfamily A member 1 (*CYP1A1*), cyclooxygenase-2 (*COX-2*), cytochrome P450 family 1 subfamily A member 2 (*CYP1A2*) and cytochrome P450 family 1 subfamily B member 1 (*CYP1B1*).^20^ Considerable evidence has suggested that a variety of endogenous metabolites, such as indole-3-acetic acid and indoxyl sulphate, were potent AHR ligands and could mediate AHR activity.^4^ Although substantial publications have demonstrated that microbial-produced metabolites contributed to various biological activities, the effect as a AHR ligand is one of their main role in many diseases.^20^ Increasing publications have suggested that hyperactive AHR signaling was involved in patients with CKD.^21,22^ However, the underlying mechanisms of gut microbiota-associated changes and microbial-produced metabolites-mediated AHR effects in CKD remain enigmatic.

Here, firstly, we identified renal function-associated bacteria in feces of healthy controls and five stages of CKD patients. Secondly, we further verified renal function-associated bacteria in feces of chronic renal failure (CRF) rats induced by adenine- and assessed the effect of *Lactobacillus johnsonii* administration on renal function and fibrosis. Thirdly, we identified renal function-associated serum metabolite (indole-3-aldehyde, IAld) in CRF rats and further verified metabolites in serum and feces of rats induced by unilateral ureteral obstruction (UUO) and 5/6 nephrectomy (NX) as well as patients with CKD. Fourthly, we found IAld-produced specific *L. johnsonii* and reveal *L. johnsonii*-produced IAld that affect renal fibrosis via inhibiting AHR pathway in CRF and UUO rats as well as 1-hydroxypyrene (HP)-stimulated HK-2 cells.

## Results

### Demographic characteristics in participants

Supplementary Fig. 1 showed the overview of current study design. The clinical parameters on healthy subjects and CKD patients with five stages were presented in supplementary Table 1. Compared with healthy controls, age and body weight did not significantly correlate with CKD progression. Systolic and diastolic blood pressure were only significantly increased in CKD5 stage. Estimated glomerular filtration rate (eGFR) marked negatively correlated with CKD progression. Renal function indexes including serum levels of creatinine, cystatin C, urea and uric acid as well as 24-h proteinuria levels and urine P/C ratio significantly positively correlated with CKD progression. In addition, the serum levels of total protein and albumin significantly negatively correlated with CKD progression. The serum levels of total cholesterol (TC), triglyceride, LDL-C and HDL-C did not marked correlate with CKD progression, indicating that the effect of lipid metabolism from liver on gut microbiota were excluded in this study. Routine blood data such as red blood cell count (RBC), hemoglobin and platelets negatively correlated with CKD progression, indicating that anemia occurred in CKD patients.

### Microbial dysbiosis correlates with declining kidney functions in patients with CKD progression

**Fig. 1:**
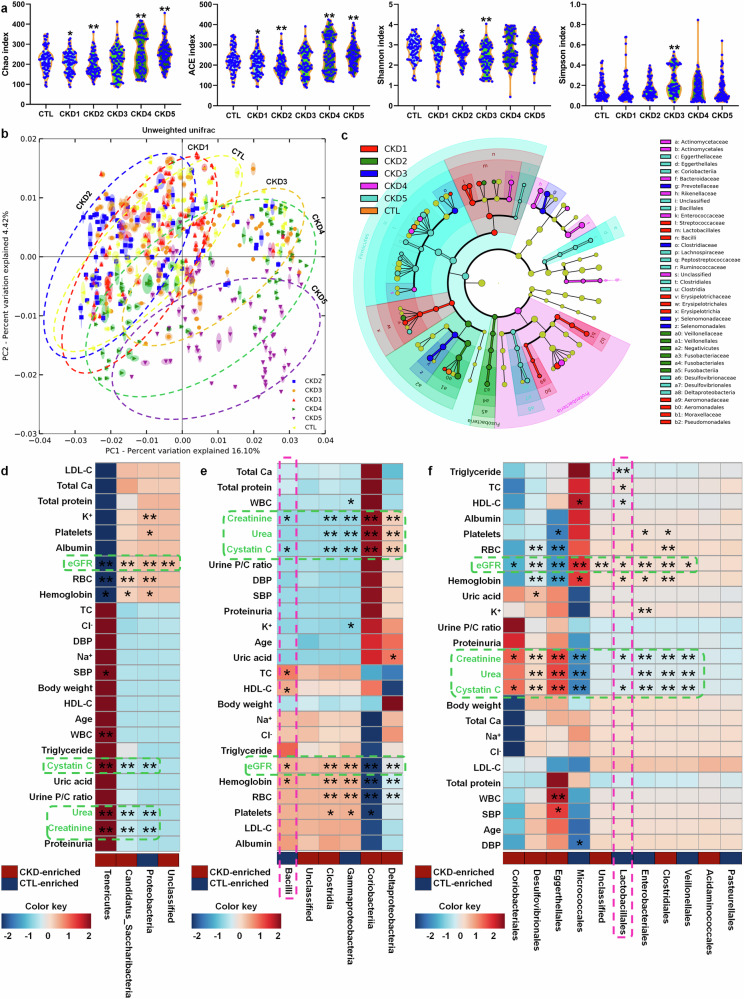
Microbial dysbiosis in patients with CKD progression. **a** α-diversity (Chao, ACE, Shannon and Simpson indexes) in controls and five stages of CKD patients (control, n = 80/group; CKD1, n = 81/group; CKD2, n = 80/group; CKD3, n = 79/group; CKD4, n = 79/group; CKD5, n = 81/group). *P < 0.05, **P < 0.01 compared with healthy controls. **b** PCoA based on unweighted UniFrac in controls and five stages of CKD patients. **c** Cladogram presented **c**rucial bacteria with an evolutionary relationship associated with CKD based on linear discriminant analysis effect size. Each circle showed a classification level from phylum to species, from the inner to outer circles. The size of each circle is proportional to relative abundance. **d** Associations between 4 significantly changed bacteria at the phylum level and 25 physiological and biochemical indexes in patients with CKD. **e** Associations between 6 significantly changed bacteria at the class level and 25 physiological and biochemical indexes in patients with CKD. **f** Associations between 11 significantly changed bacteria at the order level and 25 physiological and biochemical indexes in patients with CKD. *P < 0.05, **P < 0.01

As shown in Fig. 1a, Chao and ACE indexes reflecting species richness positively correlated with CKD progression. Shannon index negatively correlated with stages of CKD2 and CKD3. Simpson index reflecting community evenness positively correlated with CKD3 stage. Based on unweighted UniFrac, principal coordinates analysis (PCoA) showed that CKD1 and CKD2 was not separated from healthy controls, while CKD3, CKD4 and CKD5 was separated from healthy controls and CKD early stages including CKD1 and CKD2 (Fig. 1b). Of note, CKD3, CKD4 and CKD5 showed a separation tendency from CKD3 to CKD5 (Fig. 1b). Based on linear discriminant analysis effect size, cladogram showed altered microbiota in the feces of patients with CKD (Fig. 1c). These data indicated microbial dysbiosis in CKD patients.

At the six taxonomic levels, the abundances of bacteria were analyzed by using original linear regression method. Based on the values of trend P and false discovery rate (FDR), the significant changed bacteria of 4, 6, 11, 18, 28 and 46 were selected at the levels of phylum, class, order, family, genus and species in healthy subjects and five stages of patients with CKD, respectively (supplementary Table 2). Estimate values further showed that the abundances of 69 bacteria positively correlated with progressive CKD, while the abundances of 44 bacteria negatively correlated with CKD progression (supplementary Table 2). These data indicated that changed bacteria in feces were involved in progressive CKD.

### Taxonomic chain Bacilli-Lactobacillales-Lactobacillaceae-*Lactobacillus*-*L. johnsonii* correlates with renal function decline in CKD progression

**Fig. 2:**
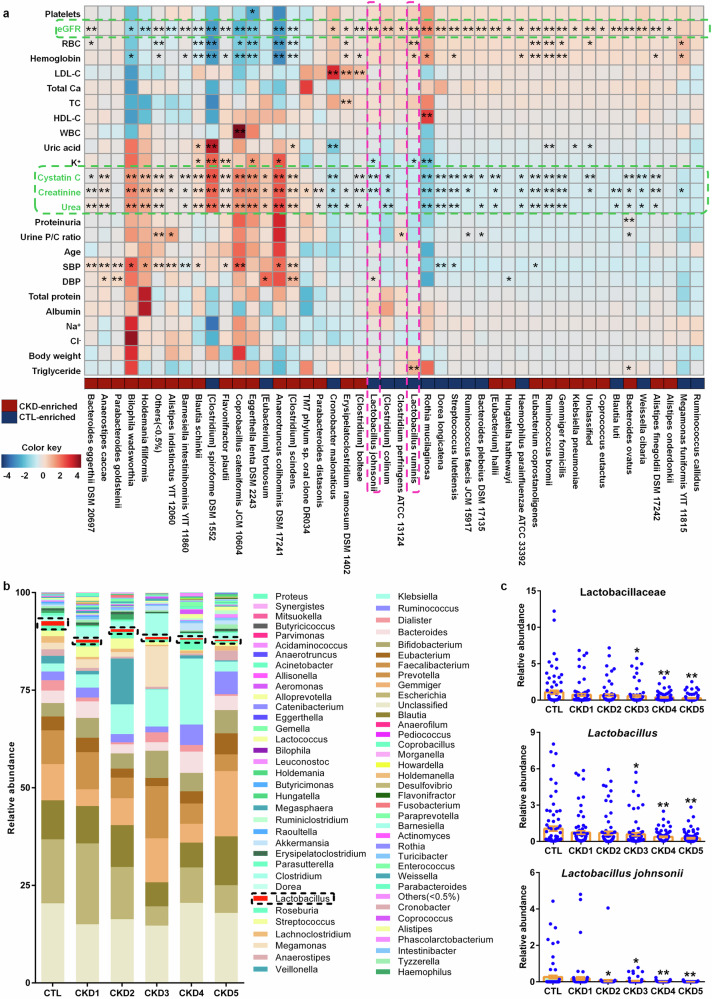
Decreased *Lactobacillus* abundance correlated with renal function decline in patients with CKD progression. **a** Associations between 46 significantly changed bacteria at the species level and 25 physiological and biochemical indexes in patients with CKD. *P < 0.05, **P < 0.01. **b** Taxonomic distributions of bacteria at the genus level in controls and five stages of CKD. As shown by dashed box. **c** The relative abundan**c**es of Lactobacillaceae, *Lactobacillus* and *Lactobacillus johnsonii* in controls and five stages of CKD patients. *P < 0.05, **P < 0.01 compared with healthy controls (control, n = 80/group; CKD1, n = 81/group; CKD2, n = 80/group; CKD3, n = 79/group; CKD4, n = 79/group; CKD5, n = 81/group). Data are represented as mean ± SEM

At the phylum level, three significantly changed bacteria correlated with eGFR, creatinine, urea and cystatin C (Fig. 1d). Similar results were also observed from class to species (Figs. 1e, f, 2a and supplementary Figs. 2, 3). Therefore, these results indicated that significantly changed bacteria are associated with declining kidney functions in patients with CKD progression. In addition, the abundances of significantly changed bacteria correlated with changes of RBC, hemoglobin and platelets from phylum to species (Figs. 1d–f, 2a and supplementary Figs. 2, 3), indicating that anemia was related to microbial dysbiosis in CKD patients. Intriguingly, the abundances of Bacilli, Lactobacillales, Lactobacillaceae, *Lactobacillus*, *L. johnsonii* and in feces positive correlated with eGFR while negative correlated with levels of creatinine and cystatin C in the serum of CKD progression (Figs. 1d–f, 2a and supplementary Figs. 2, 3). In addition, the abundance of Lactobacillaceae negative correlated with urea levels in the serum of CKD progression (supplementary Fig. 2). The abundance of *Lactobacillus ruminis* positively correlated with eGFR in CKD progression (Fig. 2a). These data suggested that an expansion taxonomic chain Bacilli-Lactobacillales-Lactobacillaceae-*Lactobacillus*-*L. johnsonii* correlated with declining kidney functions in CKD progression.

### Reduced *L. johnsonii* abundance positively correlates with declining kidney functions in CKD progression

Compared to healthy subjects, *Lactobacillus* abundance was significantly decreased in five stages of patients with CKD (Fig. 2b). Further results showed that *Lactobacillus* abundance presented a decreasingly tendency from healthy controls to CKD early stages (CKD1 and CKD2) to medium stages (CKD3) to CKD end stages (CKD4 and CKD5) (Fig. 2c). The abundance of Bacilli and Lactobacillales presented a decreasingly tendency from CKD early stages to medium stages and CKD end stages (supplementary Fig. 4). Of note, the change of relative abundance of Lactobacillaceae also showed a decreasingly tendency from healthy controls to CKD early stages to CKD end stages (Fig. 2c). Compared with healthy controls, the abundances of two *Lactobacillus* species including *L. johnsonii* and *L. ruminis* were marked decreased in five stages of CKD patients (Fig. 2c and supplementary Fig. 4). Interestingly, the abundance of *L. johnsonii* showed a decreasingly tendency from healthy controls to CKD early stages to CKD end stages (Fig. 2c). These data indicated *L. johnsonii* might have a directly correlation with renal function.

### Reduced *L. johnsonii* abundance positively correlates with declining kidney functions in CRF rats

**Fig. 3:**
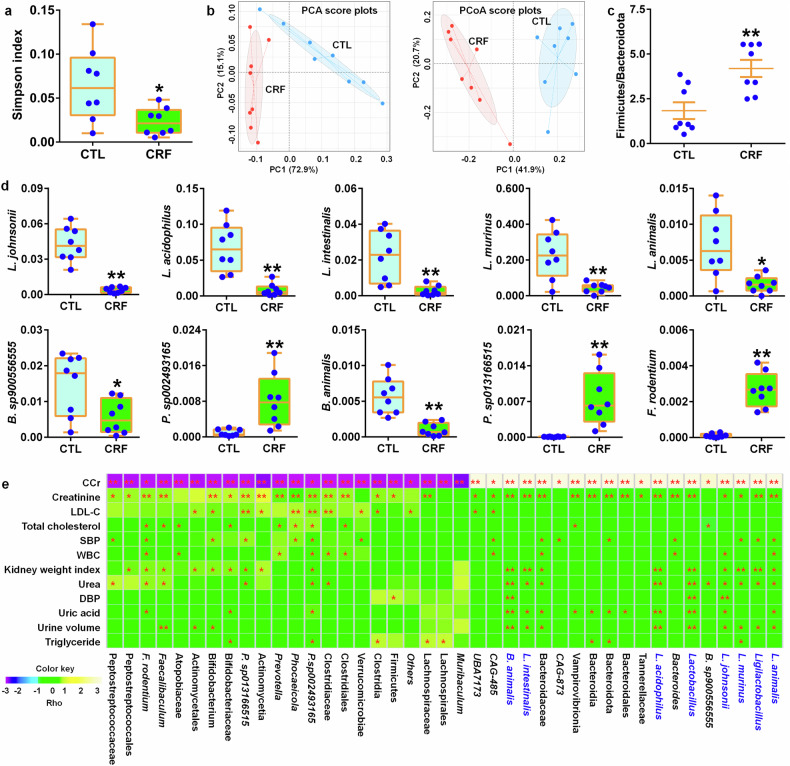
Reduced *L. johnsonii* abundance positively correlated with renal function decline in adenine-induced CRF rats. **a** Simpson index of control and CRF rats at the gene level. *P < 0.05, **P < 0.01 compared with control rats (n = 8/group). **b** PCA and PCoA of control and CRF rats at the gene level. **c** Firmicutes/Bacteroidota ratio of control and CRF rats. *P < 0.05, **P < 0.01 compared with control rats (n = 8/group). **d** The abundance of 10 significantly differential bacteria of control and CRF rats at the species level. *P < 0.05, **P < 0.01 compared with control rats (n = 8/group). **e** Heatmap of the Spearman’s rank correlation coefficient showing 41 bacteria at the different levels that correlate with physiological and biochemical indexes linked positively or negatively to CRF. Rho in the color key represents the Spearman rank correlation coefficient. *P < 0.05, **P < 0.01. Data are represented as mean ± SEM

Next, we determined whether reduced *L. johnsonii* correlated with renal function decline in the adenine-induced rats. The result showed significantly decreased Simpson index in CRF rats compared to control rats (Fig. 3a). In addition, both PCA and PCoA suggested that CRF rats could be separated from healthy control rats (Fig. 3c).

As shown in supplementary Fig. 5a, specifically, Firmicutes and Bacteroidota were mainly dominated in both control and CRF rats. Significant increased Firmicutes abundance and decreased Bacteroidota abundance were observed in feces of CRF rats compared to control rats (supplementary Fig. 5b). Firmicutes/Bacteroidota ratio, which reflects overall microbial composition in feces, was significant altered in CRF rats (Fig. 3d). The abundances of *L. johnsonii*, *L. acidophilus*, *L. intestinalis*, *L. murinus*, *L. animalis* and *Bifidobacterium animalis* showed a significant decrease while *Faecalibaculum rodentiumin* abundance showed a significant increase in CRF rats compared to control rats (Fig. 3e), indicating that altered *Lactobacillus* was involved in CRF rats.

CRF rats were completely separated from healthy control rats by 41 significantly altered bacteria (supplementary Fig. 6). As shown in Fig. 3f, the abundances of bacteria correlated with creatinine clearance rate (CCr), indicating that excessive adenine has a key effect on gut microbiota. Intriguingly, significantly reduced *Lactobacillus* and *Ligilactobacillus* abundances and its five species including *L. johnsonii*, *L. acidophilus*, *L. intestinalis*, *L. murinus* and *L. animalis* negatively correlated with increased serum levels of creatinine, urea and uric acid. Taken together, these results indicated that the depletion of *L. johnsonii* was associated with renal function decline in CRF rats.

### *L. johnsonii* treatment retards renal fibrosis

**Fig. 4:**
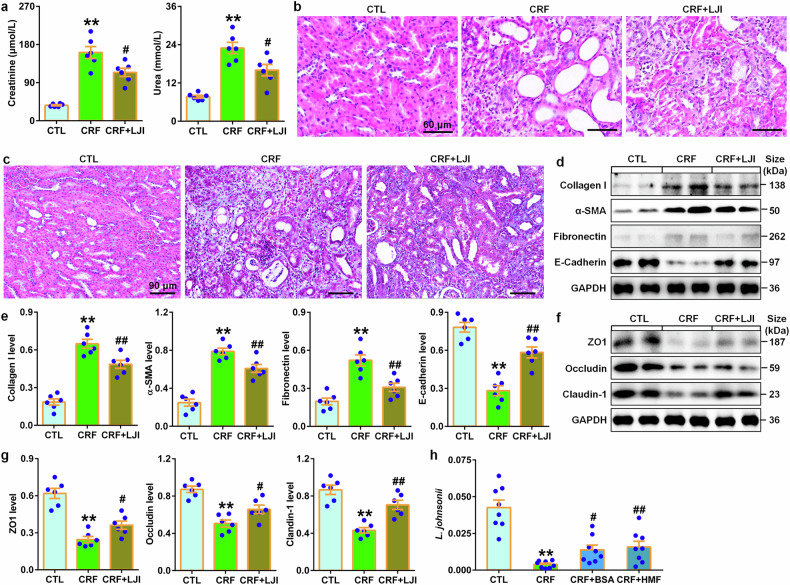
*L. johnsonii* administration ameliorates renal fibrosis in the adenine-induced CRF rats. **a** Serum levels of creatinine and urea in the control, CRF and *LJI*-treated CRF rats. **b** Images of H&E-stained kidney tissues of control, CRF and *LJI*-treated CRF rats. Scale bar, 60 μm. **c** Images of Masson’s trichrome-stained kidney tissues of control, CRF and *LJI*-treated CRF rats. Scale bar, 90 μm. **d** Protein levels of intrarenal collagen I, α-SMA, fibronectin and E-cadherin in control, CRF and *LJI*-treated CRF rats. **e** Quantitative analysis of intrarenal collagen I, α-SMA, fibronectin and E-cadherin levels in control, CRF and *LJI*-treated CRF rats. **f** Protein expression of ZO1, occludin and claudin-1 of colon tissues in control, CRF and *LJI*-treated CRF rats. **g** Quantitative analysis of ZO1, occludin and claudin-1 expression of colon tissues in control, CRF and *LJI*-treated CRF rats. **h** The abundance of fecal *LJI* in the adenine-induced CRF rats treated with BSA and HMF. *P < 0.05, **P < 0.01 compared with control rats (n = 6/group). ^#^*P* < 0.05, ^##^*P* < 0.01 compared with CRF rats (n = 6/group). Data are represented as mean ± SEM

Treatment with *L. johnsonii* ameliorated renal injury and fibrosis (Fig. 4a–c), which were accompanied by inhibiting expression of intrarenal profibrotic proteins including α-SMA, collagen I, and fibronectin as well as preserving E-cadherin levels in CRF rats (Fig. 4d, e). In colon tissues, treatment with *L. johnsonii* treatment significantly inhibited the decreased -zonula occludens-1 (ZO1), occludin and claudin-1 protein expressions in CRF rats (Fig. 4f, g). Treatment with barleriside A (BSA) and 5,6,7,8,3’,4’-hexamethoxyflavone (HMF) significantly enrich fecal *L. johnsonii* abundance in CRF rats (Fig. 4h). These findings indicate that *L. johnsonii* improved renal function and fibrosis. Therefore, targeting *L. johnsonii* may be a promising treatment to CKD.

### Microbial-derived indole derivatives from tryptophan metabolism play a critical role in CRF rats

**Fig. 5:**
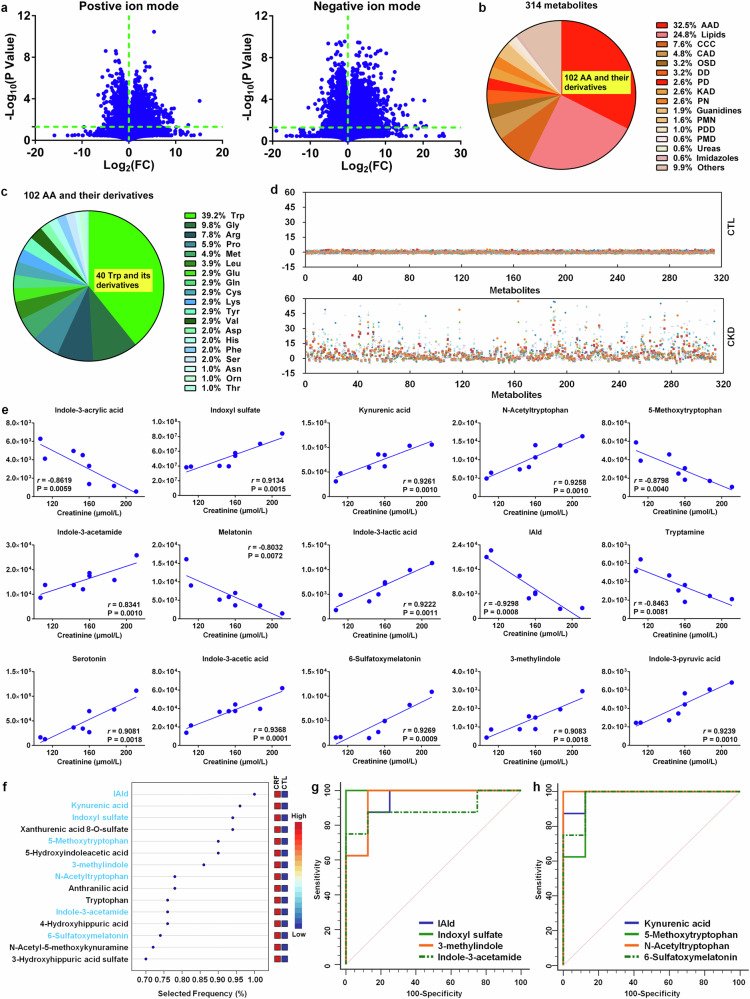
Significantly altered indole metabolites of tryptophan are correlated with renal function decline in the adenine-induced CRF rats. **a** The geometric mean ratio of each variable in CRF rats versus control rats was presented in positive and negative ion modes. **b** Pie chart based on 314 serum metabolites from control and CRF rats. **c** Pie chart based on 102 amino acid metabolites from control and CRF rats. **d** z-score plot of 314 metabolites in control and CRF rats. Each point represents an individual metabolite in one sample. Z-score plots for the data are normalized to the mean of control samples. **e** The associations between creatinine levels and intensities of tryptophan metabolites in CRF rats. Metabolites were selected based on *r* > 0.800. **f** Top metabolites presented by VIP scores based on classification method of linear support vector machines by feature ranking method of support vector machines built-in. Support vector machines models were constructed with tryptophan and its 39 metabolites from control and CRF rats. Red and blue indicate increased and decreased levels, respectively. **g** Analysis of PLS-DA based ROC curves of 4 tryptophan metabolites by gut microbiota in control and CRF rats. **h** Analysis of PLS-DA based ROC curves of 4 tryptophan metabolites by host in control and CRF rats

Compared to control rats, declining renal function was observed in CRF rats (supplementary Fig. 7a). Based on UPLC-HDMS, we obtained 13251 and 21696 fragment ions in positive and negative ion modes of the serum from CRF and control rats, respectively. Initially, fragment ions were chosen based on the *P* value. 2278 and 6030 fragment ions had *P* < 0.05 in positive and negative ion modes, respectively (Fig. 5a). PCA score plots displayed that 2278 and 6030 fragment ions could discriminate CRF group from control group, suggesting that serum metabolic pattern showed a significantly change in CRF rats (supplementary Fig. 7b). After removing xenobiotics, 314 metabolites were identified based on our previously publications (supplementary Table 3).^23,24^ According to chemical structure of metabolites, we classified 314 metabolites and showed that the metabolites mainly included 102 amino acids and their metabolites, 78 lipids, 24 carbohydrates, 33 nucleosides, nucleotides and their derivatives and 8 amines. In addition, the remaining other 30 metabolites were not be grouped into specific chemical structures (Fig. 5b and supplementary Table 3). Of note, the most amounts of amino acids and their derivatives mainly include 40 tryptophan and its metabolites, as well as 10 glycine derivatives, 8 arginine derivatives and 6 proline derivatives (Fig. 5c). PCA plots and dendrogram displayed that 314 metabolites could separate adenine-induced group from control group (supplementary Fig. 7c, d). In addition, prediction class probability showed that eight adenine-induced rats were correctly grouped with the sensitivity of 100% and eight control rats were grouped in control area with the specificity of 100% (supplementary Fig. 7e). The z-score results displayed that the most of 314 metabolites showed an increase in CRF rats (Fig. 5d). Taken together, altered serum metabolic profiles were related to the perturbation of amino acids and their derivatives in CRF rats.

We further uncover biological functions of changed metabolites. The top seven pathways include the following: 1) the biosynthesis of arginine; 2) the metabolism of tryptophan; 3) the metabolism of alanine, glutamate and aspartate; 4) the metabolism of phenylalanine; 5) the biosynthesis of phenylalanine, tryptophan and tyrosine; 6) the metabolism of hypotaurine and taurine; 7) the synthesis and degradation of ketone bodies (supplementary Fig. 8a and supplementary Table 4). Changes in these pathways in CRF indicated that certain amino acid metabolism was severely disturbed in adenine-induced rats. A pathway enrichment overview of changed metabolites highlighted that tryptophan metabolism showed a significant enrichment in serum metabolomics in CRF versus control rats (supplementary Fig. 8b). Therefore, these results showed that tryptophan metabolism played a critical and central effect in CRF rats.

We further confirm the effect of tryptophan metabolism on CRF. The results showed that tryptophan and its 39 derivatives could discriminate CRF rats from control rats (supplementary Fig. 9a, b). Predicted class probabilities also showed 87.5% sensitivity and 100% specificity (supplementary Fig. 9c). CRF and control rats could be clearly separated and most of metabolites were marked increased in CRF rats (supplementary Fig. 9d). The levels of 8 metabolites such as tryptophan, 5-methoxytryptophan, melatonin, indole-3-acrylic acid and IAld are significantly decreased, while the levels of other 32 metabolites are marked increased in serum of CRF rats (supplementary Fig. 10a, b). Taken together, these analyses demonstrated that tryptophan metabolism perturbation played a critical role in CRF rats.

To investigate whether serum tryptophan and its 39 derivatives correlated with serum creatinine level, we tested associations between tryptophan derivatives and creatinine in CRF rats. Among these metabolites, the intensities of 15 metabolites displayed a marked correlation with serum creatinine level and had correlation coefficient of more than 0.800 (Fig. 5e). Other 25 metabolites presented a low correlation with serum creatinine level (supplementary Fig. 11a, b). Interestingly, 14 of 15 metabolites belonged to indole derivatives, which were microbial tryptophan metabolites. 15 metabolites had a top-ranked variable importance in projection scores overlapped between CRF and controls (Fig. 5f). Among 15 metabolites, eight metabolites including IAld, kynurenic acid, indoxyl sulfate, 5-methoxytryptophan, 3-methylindole, N-acetyltryptophan, indole-3-acetamide and 6-sulfatoxymelatonin had a significant correlation with serum creatinine level. Except for kynurenic acid, other 7 metabolites structurally belonged to indole derivatives form tryptophan metabolism by gut microbiota. Additionally, ROC analyses showed that 8 metabolites had an area under curve (AUC) of 0.89 or greater (Fig. 5g, h and supplementary Table 5). Therefore, these results displayed that renal fibrosis was closely related to gut microbial dysbiosis in CRF rats.

### Aberrant microbial-derived indole derivatives from tryptophan metabolism are associated with patients with CKD

**Fig. 6:**
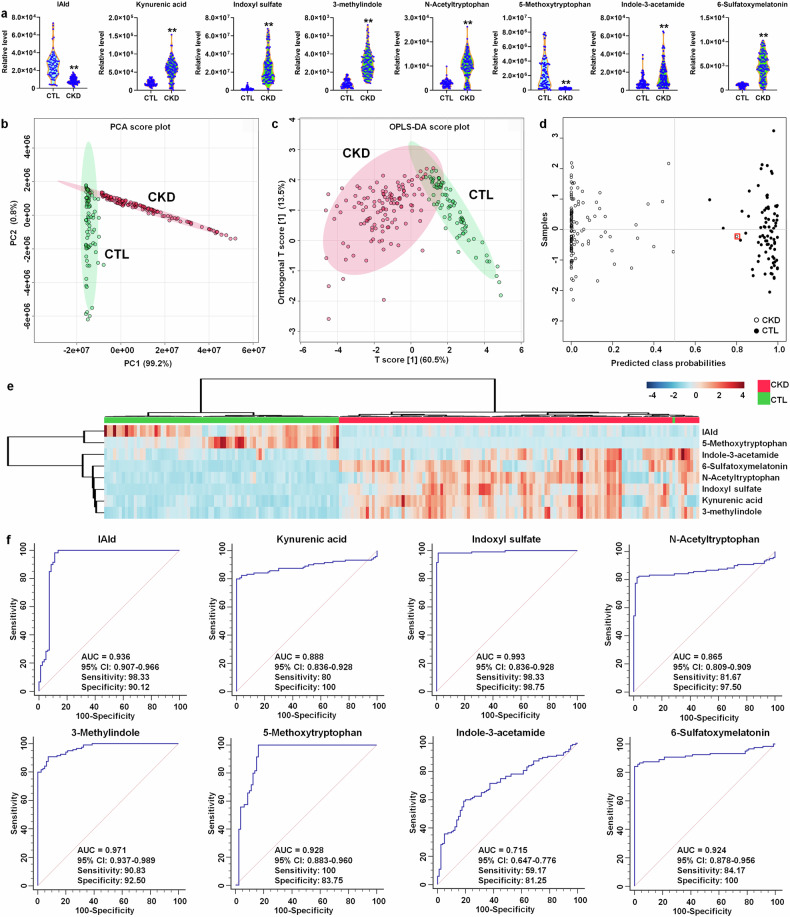
Aberrant microbial-derived indole derivatives from tryptophan metabolism were associated with CKD patients. **a** Violin plot showing the relative levels of eight tryptophan metabolites in controls and CKD patients. In the plot, the median, 75th percentile and 25th percentile are represented by the center line, upper dashed line and lower dashed line, respectively. *P < 0.05, **P < 0.01 compared with controls (controls, n = 80/group; CKD, n = 120/group). **b** PCA score plots of eight tryptophan metabolites from 80 controls and 120 CKD patients. **c** OPLS-DA score plots of eight tryptophan metabolites from 80 control and 120 CKD patients. **d** Diagnostic performances of eight tryptophan metabolites based on the support vector machines method. The black circles with red squares are for the incorrectly predicted samples in control group. **e** Heatmap of eight tryptophan metabolites between controls and CKD patients. **f** Analysis of PLS-DA based ROC curves of eight tryptophan metabolites in controls and CKD patients

Clinical data of 80 health controls and 120 CKD patients were presented in supplementary Table 6. CKD patients showed marked decreased renal function. The levels of IAld and 5-methoxytryptophan displayed a significant decrease, while the levels of other 7 metabolites displayed a significantly increase in the serum of CKD patients (Fig. 6a), which were consistent with the results of CRF rats. Eight tryptophan metabolites could discriminate CKD patients from healthy controls (Fig. 6b, c). Predicted class probabilities showed 99.2% sensitivity and 100% specificity (Fig. 6d). In addition, Heatmap suggested that CKD and control samples could be separated (Fig. 6e). Additionally, ROC analysis suggested that the eight metabolites displayed a high AUC value with high specificity and sensitivity (Fig. 6f).

### Reduced serum IAld level correlates with renal function decline in CKD patients and rat models

**Fig. 7:**
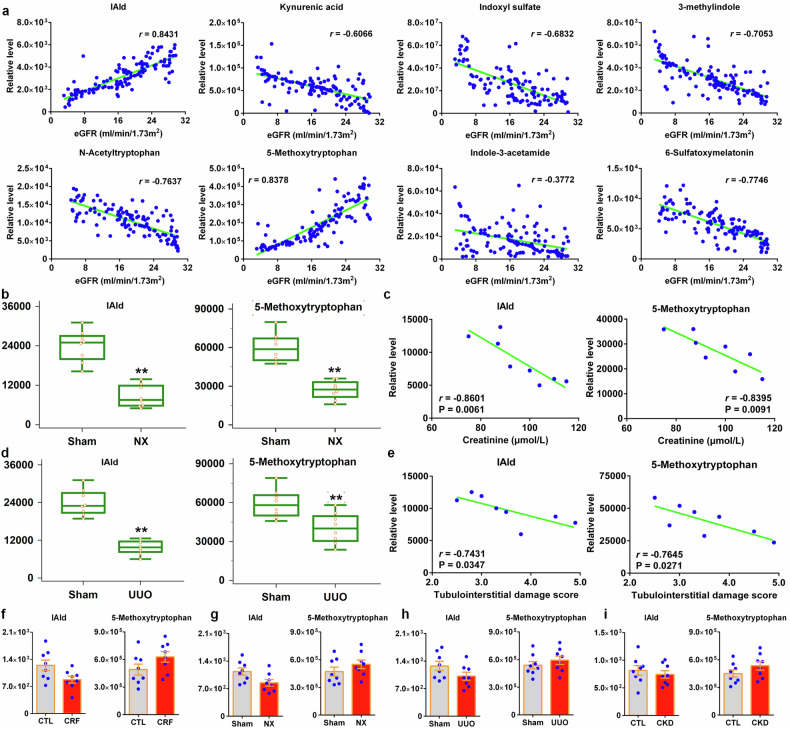
Reduced serum IAld level correlated with renal function decline in CKD patients and rat models. **a** Associations between levels of eight tryptophan metabolites and eGFR in CKD patients. **b** Combined box-and-whisker and dot plot of levels of IAld and 5-methoxytryptophan in serum of Sham and NX rats. Mean values are presented by horizontal bars. The whiskers indicate the maximum and minimum points (*n* = 8/group). **c** The associations between creatinine levels and IAld and 5-methoxytryptophan levels in NX rats. **d** Combined box-and-whisker and dot plot of levels of IAld and 5-methoxytryptophan in serum of Sham and UUO rats. Mean values are presented by horizontal bars. The whiskers indicate the maximum and minimum points. *P < 0.05, **P < 0.01 compared with Sham rats (*n* = 8/group). **e** The associations between tubulointerstitial damage score and IAld and 5-methoxytryptophan levels in UUO rats. **f** Relative levels of IAld and 5-methoxytryptophan in feces of control and CRF rats (*n* = 8/group). **g** Relative levels of IAld and 5-methoxytryptophan in feces of Sham and NX rats (*n* = 8/group). **h** Relative levels of IAld and 5-methoxytryptophan in feces of Sham and UUO rats (*n* = 8/group). **i** Relative levels of IAld and 5-methoxytryptophan in feces of healthy controls and CKD patients (*n* = 8/group). Data are represented as mean ± SEM

Two metabolites including IAld and 5-methoxytryptophan displayed a good correlation with eGFR and showed correlation coefficient of more than 0.800 (Fig. 7a). The levels of IAld and 5-methoxytryptophan were significant decreased in NX rats compared with sham rats (Fig. 7b), and showed a significant correlation with serum creatinine level in NX rats (Fig. 7c). Similar results were also demonstrated in UUO and sham rats (Fig. 7d, e). The levels of fecal IAld and 5-methoxytryptophan in feces were slightly decreased in rats treated by adenine, NX and UUO (Fig. 7f-h). Similar results were also demonstrated in CKD patients (Fig. 7i). These data indicated the reduced serum IAld level correlated with renal function decline in CKD.

### Reduced *L. johnsonii* positively correlates with decreased serum IAld level in CRF rats

Catalog pathway showed that altered bacteria were associated with significantly downregulated biosynthesis of secondary metabolites in CRF rats (supplementary Table 7). In addition, the pathways such as metabolism of amino acids were also involved in metabolic disorders of gut microbial dysbiosis (supplementary Table 7). We further performed correlation analysis between renal function-associated bacteria and creatinine-associated 15 tryptophan derivatives. Reduced *Lactobacillus* and its species correlated with serum microbial-derived indole derivatives including IAld and indole-3-pyruvic acid (IpyA) in CRF rats (supplementary Fig. 12a). Of note, reduced *L. johnsonii* abundance positively correlated with reduced serum IAld level in CRF rats (supplementary Fig. 12a). KEGG Orthology analysis indicated significant altered a number of enzymes were related to tryptophan catabolism in CRF rats. As shown in supplementary Fig. 12b, Aromatic amino acid transaminase (ArAT, K00832) was a key enzyme in bacteria, including *lactobacilli*. Tryptophan could be converted to IpyA by ArAT. Significantly increased ArAT bioactivity indicated an increase in IpyA generation (supplementary Fig. 12c). In addition, significantly increased branched-chain amino acid aminotransferases (K00826) bioactivity might also increase IpyA generation. These results were consistent with increased IpyA levels in serum of CRF rats. Indolepyruvate decarboxylase (K04103) was an important enzyme for converting IpyA to indole-3-acetaldehyde. Significantly decreased indolepyruvate decarboxylase bioactivity suggested a decrease in generation of indole-3-acetaldehyde and even further IAld (supplementary Fig. 12c). Moreover, significantly decreased aromatic-L-amino-acid/L-tryptophan decarboxylase (K01593) bioactivity indicated a decrease in IAld generation (supplementary Fig. 12c). Collectively, these findings indicated that adenine-induced CRF had a key effect on IpyA pathway, one of the main pathways for IAld generation from tryptophan (tryptophan→IpyA→IAld). These data suggest that IpyA pathway played a key role in CRF.

### The renoprotective effect of IAld and *L. johnsonii* administration are associated with suppressing AHR signaling

**Fig. 8:**
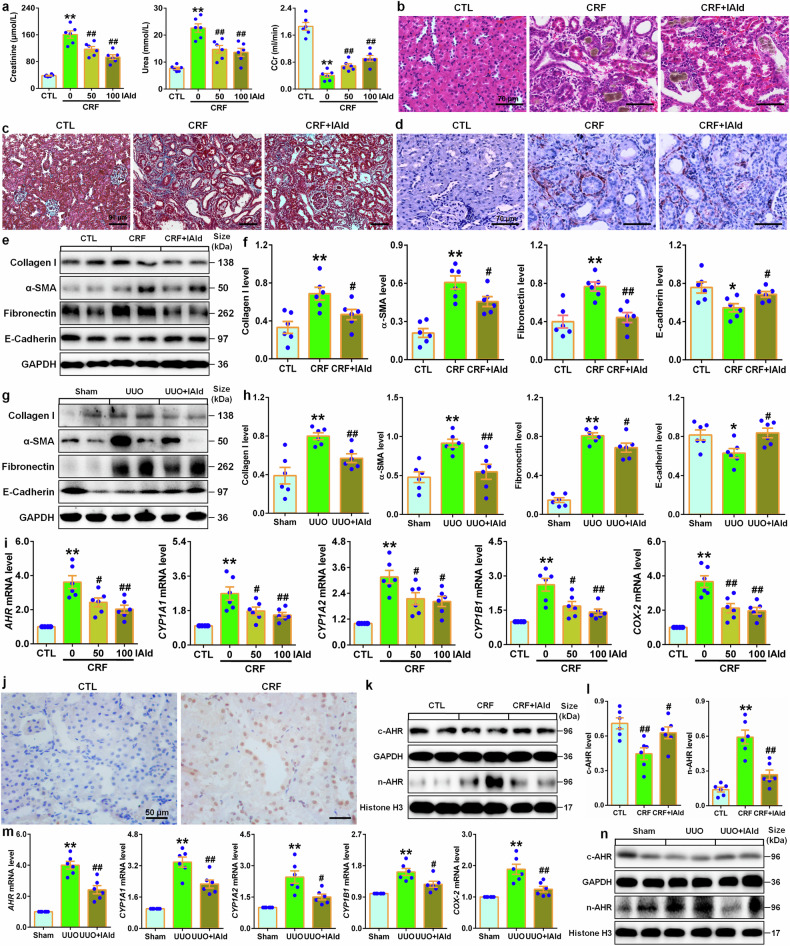
The renoprotective effect of IAld and *L. johnsonii* administration were associated with inhibiting AHR signaling pathway. **a** Serum creatinine and urea levels and CCr in the control, CRF and different doses of IAld-treated CRF rats. **b** Images of H&E-stained kidney tissues in the control, CRF and IAld-treated CRF rats. Scale bar, 70 μm. **c** Images of Masson’s trichrome-stained kidney tissues in the control, CRF and IAld-treated CRF rats. Scale bar, 90 μm. **d** Immunohistochemical findings with anti-α-SMA of kidney tissues in the control, CRF and IAld-treated CRF rats. Scale bar, 70 μm. **e** Protein expression of collagen I, α-SMA, fibronectin and E-cadherin of kidney tissues in control, CRF and IAld-treated CRF rats. **f** Quantitative analysis of collagen I, α-SMA, fibronectin and E-cadherin of kidney tissues in the control, CRF and IAld-treated CRF rats. **g** Protein expression of collagen I, α-SMA, fibronectin and E-cadherin of kidney tissues in the Sham, UUO and IAld-treated UUO rats. **h** Quantitative analysis of collagen I, α-SMA, fibronectin and E-cadherin of kidney tissues in the Sham, UUO and IAld-treated UUO rats. **i** The mRNA levels of AHR and its target genes including *CYP1A1*, *CYP1A2*, *CYP1B1* and *COX-2* of kidney tissues in the control, CRF and different doses of IAld-treated CRF rats. **j** Immunohistochemical analysis with anti-AHR of kidney tissues in the control and CRF rats. **k** Protein expression of AHR in cytoplasm and nuclei of kidney tissues in the control, CRF and IAld-treated CRF rats. **l** Quantitative analysis of AHR expression in cytoplasm and nuclei of kidney tissues in the control, CRF and IAld-treated CRF rats. **m** Obstructed intrarenal mRNA levels of *AHR* and its target genes including *CYP1A1*, *CYP1A2*, *CYP1B1* and *COX-2* in the Sham, UUO and IAld-treated UUO rats. **n** Protein expression of AHR in cytoplasm and nuclei of kidney tissues in the Sham, UUO and IAld-treated UUO rats. *P < 0.05; **P < 0.01 compared with control or sham rats (*n* = 6/group). ^#^*P* < 0.05; ^##^*P* < 0.01 compared with CRF or UUO rats (*n* = 6/group). Data are represented as mean ± SEM

Treatment with two doses of IAld marked lowered serum creatinine and urea levels while increased CCr in CRF rats (Fig. 8a). IAld ameliorated renal injury and fibrosis (Fig. 8b–d), which were accompanied by inhibiting expression of intrarenal profibrotic proteins (Fig. 8e, f). Similar results were presented in UUO rats (Fig. 8g, h and supplementary Fig. 13a). In colon tissues, IAld treatment significantly inhibited decreased protein expressions of intestinal epithelial cells in CRF rats (supplementary Fig. 14). Increasing evidence has suggested that a number of tryptophan catabolites, such as indoxyl sulfate, IAld and IAA, were demonstrated to be AHR ligands that mediated AHR signaling pathway in various diseases.^20,25^ Our latest findings have demonstrated hyperactive AHR signaling in CRF rats.^26^ Both doses of IAld significantly inhibited mRNA expression of AHR and its downstream four genes (Fig. 8i), which were accompanied by significantly inhibiting intrarenal AHR nuclear translocation (Fig. 8j). Further study showed that IAld treatment significant inhibited increased nuclei AHR protein expression in CRF rats (Fig. 8k, l). Similar results were presented in UUO rats (Fig. 8m, n supplementary Fig. 13b). Collectively, these data suggested that inhibiting AHR signaling pathway was involved in renoprotective effect of IAld and LJI.

### IAld protects against renal fibrosis by suppressing AHR signaling

**Fig. 9:**
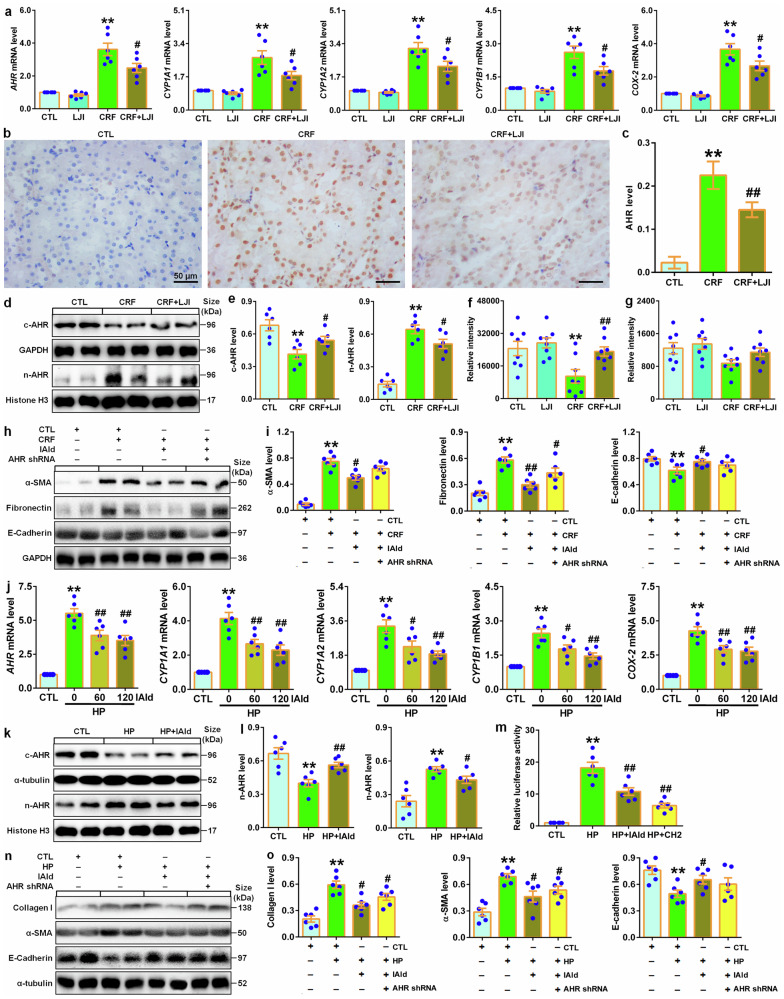
IAld protected against renal fibrosis through inhibiting AHR signaling pathway. **a** Intrarenal mRNA levels of *AHR* and its target genes including *CYP1A1*, *CYP1A2*, *CYP1B1* and *COX-2* in the control, *LJI*, CRF and *LJI*-treated CRF rats. **b** Immunohistochemical analysis with anti-AHR of kidney tissues in the control, CRF and *LJI*-treated CRF rats. Scale bar, 50 μm. **c** Quantitative analysis of immunohistochemistry with anti-AHR of kidney tissues in the control, CRF and *LJI*-treated CRF rats. **d** Protein expression of AHR in cytoplasm and nuclei of kidney tissues in the control, CRF and *LJI*-treated CRF rats. **e** Quantitative analysis of AHR expression in cytoplasm and nuclei of kidney tissues in the control, CRF and *LJI*-treated CRF rats. **f** Serum IAld relative intensity in the control, *LJI*, CRF and *LJI*-treated CRF rats. **g** Fecal IAld relative intensity in the control, *LJI*, CRF and *LJI*-treated CRF rats. **h** Protein expression of α-SMA, fibronectin and E-cadherin of kidney tissues in indicated groups. **i** Quantitative analyses of α-SMA, fibronectin and E-cadherin of kidney tissues in indicated groups. **j** The mRNA expression levels of AHR and its target genes including *CYP1A1*, *CYP1A2*, *CYP1B1* and *COX-2* in indicated groups. **k** Protein levels of AHR in cytoplasm and nuclei of HK-2 cells in indicated groups. **l** Quantitative analysis of AHR expression in in cytoplasm and nuclei of HK-2 cells in indicated groups. **m** Luciferase assays of AHR of HK-2 cells in indicated groups. **n** Protein levels of α-SMA, fibronectin and E-cadherin of HK-2 cells in indicated groups. **o** Quantitative analyses of α-SMA, fibronectin and E-cadherin of HK-2 cells in indicated groups. Dot presents the single data results in bar graph. *P < 0.05; **P < 0.01 compared with control rats or cells (n = 6/group). ^#^*P* < 0.05; ^##^*P* < 0.01 compared with adenine-induced CRF rats or HP-induced HK-2 cells (*n* = 6/group). Data are represented as mean ± SEM

Treatment with *L. johnsonii* significantly inhibited mRNA expression of AHR and its downstream four genes in the kidney tissues of CRF rats (Fig. 9a), which were accompanied by inhibiting intrarenal AHR nuclear translocation (Fig. 9b, c). Further study showed that treatment with *L. johnsonii* inhibited increased nuclei AHR protein expression in CRF rats (Fig. 9d, e). In addition, treatment with *L. johnsonii* significantly increased serum IAld level but not affected fecal IAld level in CRF rats (Fig. 9f, g). These data suggested that *L. johnsonii* treatment inhibited AHR signaling pathway. In contrast, IAld inhibitory effect was partially attenuated in AHR knockout-treated CRF mice (Fig. 9h, i).

Cell viability analysis showed that IAld did not significantly affect cell viability at 7.5–240 μM within 48 h in HK-2 cells (supplementary Fig. 15). In CKD patients, our recent publication identified HP, an endogenous metabolite, mediated renal fibrosis via AHR signaling pathway.^26^ Treatment with two concentrations of IAld significantly suppressed mRNA expression of AHR and its downstream four target genes in HP-induced HK-2 cells (Fig. 9j), which were accompanied by significantly inhibiting AHR nuclear translocation (Fig. 9k, l). Similarly, IAld treatment inhibited pro-fibrotic protein expressions and preserves E-cadherin protein expression in HK-2 cells induced by HP (Fig. 9n, o), in contrast, IAld inhibitory effect was partially mitigated in AHR siRNA-treated HK-2 cells induced by HP compared to CTL siRNA (Fig. 9n, o). Collectively, these results demonstrated that microbial-derived IAld improved renal function and abolished renal fibrosis through attenuating AHR signaling.

## Discussion

**Fig. 10:**
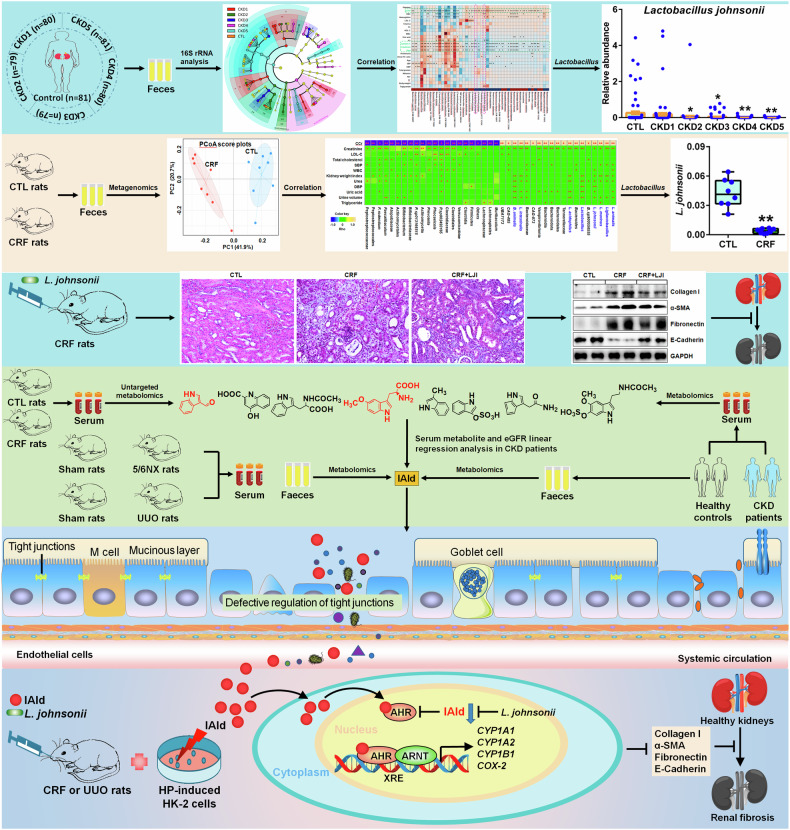
Renoprotective effects of *L. johnsonii* and molecular mechanism by AHR inhibition via IAld in CKD. An elongation chain Bacilli-Lactobacillales-Lactobacillaceae-*Lactobacillus*-*L. johnsonii* correlated with renal function decline in patients with CKD progression. Reduced *L. johnsonii* abundance was further observed in feces of CRF rats. *L. johnsonii* supplementation ameliorated renal injury and fibrosis. Eight metabolites were associated with kidney function. Serum IAld was further verified by rats induced by NX and UUO as well as CKD patients. IAld levels correlated with eGFR in CKD patients. IAld were produced by *L. johnsonii* via IpyA metabolic pathway. Treatment with IAld or *L. johnsonii* could ameliorate renal injury and fibrosis via AHR signaling pathway in CRF and/or UUO rats as well as HP-stimulated HK-2 cells. Parts of this schematic was created using Servier Medical Art, CC BY 4.0

We first identified that reduced *L. johnsonii* abundance in feces positively correlated with eGFR in healthy controls and five stages of CKD patients. Our study further demonstrated the reduced *L. johnsonii* abundance in feces of CRF rats (Fig. 10). Two previous studies showed a decrease in *L. johnsonii* abundance in feces of CKD rats induced by cationic bovine serum albumin and NX.^27,28^ In addition, Zhang et al s a decrease in *L. johnsonii* abundance in feces of mice with cisplatin-induced renal injury.^29^ Our current study demonstrated that *L. johnsonii* supplementation ameliorated renal injury. A previous study reported that *L. johnsonii* N6.2 supplementation ameliorated indigestion and cephalic syndromes in healthy subjects with no type 1 diabetes risk.^30^ *L. johnsonii* N6.2 consumption is well tolerated in adult subjects. These findings suggested the safety and feasibility of *L. johnsonii* N6.2 in preventing adult individuals at risk for type 1 diabetes.

Several studies have highlighted that *L. johnsonii* supplementation displayed beneficial effects on patients.^31–33^ *L. johnsonii* La1 supplementation reduced *Helicobacter pylori* colonization in adult volunteers.^31^ Similar result showed that *L. johnsonii* La1 supplementation showed an inhibitory effect on *Helicobacter pylori* colonization in asymptomatic volunteers.^32^ Moreover, *L. johnsonii* La1 supplementation showed a significant but low suppressive effect in colonized children.^32^ However, multicenter clinical trial suggested that *L. johnsonii* La1 in patients with Crohn’s disease failed to improve early endoscopic recurrence.^34^ Van Gossum et al reported *L. johnsonii* N6.2 had no adverse events in healthy individuals.^34^ In addition. no serious adverse events were observed in combining *L. johnsonii* EM1 with levocetirizine for treating perennial allergic rhinitis.^35^ So far, no study showed the beneficial and harmful effects of *L. johnsonii* on patients with CKD. There are no side effects of long-term *L. johnsonii* supplementation in CKD patient. Based on animal models, extensive studies have suggested that *L. johnsonii* supplementation showed beneficial effects in various diseases.^36^ Xin et al reported that *L. johnsonii* BS15 supplementation ameliorated renal damage induced by high fluoride exposure.^37^ *L. johnsonii* inhibited oxidative stress and antiinflammation in the kidney tissue of mice treated with high fluoride exposure.^37^ Moreover, previous study showed that a mixture of *Lactobacillus* strains including *L. johnsonii* elevated IL-10 and reduced major immune deposit IgG2a as well as disturbed Treg-Th17 balance towards a Treg phenotype in mice lupus nephritis.^38^ In addition, the latest study showed *L. johnsonii* abundance positively correlated with the activities of Na^+^-K^+^-ATP-ase and Ca^2+^-Mg^2+^-ATP-ase in serum of deficiency kidney-yang syndrome mice with diarrhea.^39^ Moreover, Liu et al reported that fecal microbiota transplantation improved *L. johnsonii* abundance in NX rats.^27^ Therefore, these findings suggested that reduced *L. johnsonii* abundance could indicate changes in renal function in CKD progression and it might be a CKD therapy target. Although there are a number of reports of *L. johnsonii* showing efficacy in animal models with CKD, there are no approved commoditization products of *L. johnsonii* and its metabolites including IAld sold on the market with very few in clinical trials. Therefore, clinical translation needs to be performed to realize the potential of *L. johnsonii* and IAld.

Previous study demonstrated significantly decreased plasma IAld levels in patients with advanced atherosclerosis compared with control cohorts.^40^ In addition, IAld levels were significantly reduced in lesional and non-lesional skin of patients with atopic dermatitis compared with healthy subjects.^41^ A recent publication that elevating IAld level in feces was related to resistance to radiation- mediated pathology resulting to gastrointestinal, hematopoietic and cerebro-vascular damage.^42^ Taken together, these findings suggest significantly altered IAld levels in disease milieu.

Subsequently, we first demonstrated that IAld administration ameliorated renal fibrosis by suppressing AHR signaling. Our previous studies showed the upregulated nuclei AHR protein expression in CKD.^26,43^ In addition, the latest study suggested that the upregulation of mRNA expression of *CYP1A1* and *CYP1B1* were demonstrated in peripheral artery disease patients and mice with CKD.^44^ Recent study showed that indoxyl sulfate and kynurenine inhibited β-catenin protein expression and decreasing Wnt activity through AHR expression in microvascular endothelial cells.^45^ Further study demonstrated that adenine-induced hindlimb ischemia CKD and indoxyl sulfate-induced mice suggested downregulated β-catenin protein expression in capillaries, which were associated with serum levels of indoxyl sulfate and kynurenine and AHR effect in endothelial cells.^45^ Moreover, treatment with AHR inhibitor improved postischemic angiogenic response in CKD mice and serum levels of tryptophan catabolites and AHR-mediating activity in endothelial cells elevating adverse limb risk.^45^ These findings indicated that IAld protected against multi-organ fibrosis, which were consistent with common molecular mechanisms of multi-organ fibrosis.^46^ There is no report about the advantages or disadvantages of IAld supplementation compared with current CKD treatment and side effects of long-term IAld supplementation in CKD patients. For epithelial barrier injury, our current study showed that IAld administration significantly inhibited the downregulation of protein expressions of occludin, ZO1 and claudin-1 of colon tissues in CRF rats. Several seminal publications demonstrate that IAld could improve age-dependent and intestinal homeostasis through AHR signaling pathway,^47^ which indicated IAld in preventing age-associated morbidity that was associated with the epithelial barrier protective effect of IAld. In addition, IAld feeding reversed antifungal resistance upon infection and mitigated colitis in wild-type mice. However, these effects were not observed in *AHR*^*-/-*^ mice. The current study and that of others suggests that IAld protects against renal injury through improving intestinal epithelial barrier functions and suppressing AHR signaling pathway (Fig. 10).

In summary, we first found that reduced *L. johnsonii* abundance positively correlated with declining renal function in CKD. We first identified that serum level of reduced IAld positively correlated with declining renal function in rats and CKD patients. Our study demonstrated that supplementation of *L. johnsonii* and IAld ameliorated renal injury and fibrosis through AHR signaling pathway. Taken together, *L. johnsonii* supplementation protected against renal fibrosis through inhibiting AHR signaling pathway via increasing IAld level. This study provides a understanding of how microbial-produced tryptophan metabolites affect host disease mechanisms and discovers potential pathways for CKD treatment.

## Materials and methods

### Participants

This is a cross-sectional study in 400 CKD patients containing five stages (CKD1 = 81, CKD2 = 80, CKD3 = 79, CKD4 = 79, CKD5 = 81) by using eGFR equation and 80 age- and sex-matched healthy controls. The samples of overnight fasting serum, 24-h urine and feces are collected between 2016 and 2018 from Shaanxi Traditional Chinese Medicine Hospital, Xi’an Peoples Hospital and Baoji Central Hospital. Sample collection is approved Shaanxi Traditional Chinese Medicine Hospital (Permit Number: SXSY-235610). The causes of CKD were diabetes, hypertension, chronic glomerulonephritis, idiopathic membranous nephropathy, immunoglobulin A nephropathy, obstructive uropathy and chronic tubulointerstitial nephritis. Patients with CKD do not receive antibiotic intervention for at least one month before sample collection. All recruited participants do not take any food including probiotics such as yogurt within one week before sample collection. We exclude patients with acute kidney injury, lung injury, liver disease, neurodegenerative disorders, colitis, active vasculitis or cancer in this study. Healthy controls are excluded if they have any of the following diseases: kidney dysfunction, hypertension, diabetes, cardiovascular diseases or under regular medications. Serum and urinary are stored at -80 °C. Blood and urine biochemical data were detected in clinical laboratory.

### 16S rRNA sequencing analysis

#### DNA extraction

The feces are collected and frozen at −80 °C. Total DNA is extracted by using QIAamp Fast DNA stool minikit (Qiagen, Hilden, Germany) for 16 S rRNA sequencing.

#### 16S rRNA gene amplicon sequencing

Hypervariable V4 region from 16 S rRNA gene in bacteria is configured with specific 515 F and 806 R primers with their barcodes based on the previous methods.^48^ A sequencing library was established by using TruSeq DNA PCR-Free sample preparation kit and sequenced on Illumina HiSeq 2500 platform to generate 250-bp paired-end reads.

#### 16S rRNA sequencing data processing

Raw paired-end reads are analyzed by using FLASH and filter with mothur. High-quality sequences with no less than 97% similarity are assigned to same OTUs by USEARCH Global annotated to the Greengenes database via RDP Classifier. We further carry out BLAST on OTU sequences with cut-off of 95% coverage and 98% identity in order to obtain higher resolution species.

#### Construction of bacterial taxonomy and KO profiles

Based on the different levels, the relative abundances of bacterial taxonomy are then obtained. The relative abundances of the bacterial taxonomy and KEGG Orthology are obtained by using relative abundance of their respective genes.^49^

#### Gene count, α-diversity and β-diversity

Total gene count from each sample is calculated based on the previous methods.^50^ α-diversity is calculated based on the previous methods.^51^ Unweighted UniFrac PCoA is obtained by QIIME (v1.80).

### Validation experiment for gut microbiota

#### Animal model study

6–8 weeks old male SD rats with weighing 190–210 g are purchased from Central Animal Breeding House of Xi’an Jiaotong University (Xi’an, China). Adenine-induced CRF rats is reproduced as described in our previous publications.^23^ Briefly, rats are randomly divided into healthy control group and CRF group (n = 8/group). CRF group is given 200 mg/kg/day adenine by oral administration for three weeks, which produced renal failure in rats. All rats are killed after anaesthetised with 10% urethane at week 3. For clinical biochemical analysis, serum and 24-h urine are obtained, while the kidney and colon tissues in the CRF rats are collected for pathologic, mRNA and Western blot analyses. Serum and fecal samples are collected from all rats for metabolomic and metagenomic sequencing analyses, respectively. All animal care and experimental procedures are approved by Ethics Committee for Animal Experiments of Zhejiang Chinese Medical University (No. 20200713-06).

#### Metagenomic analysis

The fecal samples of rats were performed by using metagenomic analysis. The DNA extraction, library construction and sequencing, metagenomic sequence processing, taxonomic and functional profiling and diversity analysis were described in our previous publication.^28^

#### Metabolomic analysis

The samples of serum and feces from rats and humans are analyzed using a Waters ACQUITY HSS T3 column (2.1 mm × 100 mm, 1.8 μm, UK) with a Waters Acquity^TM^ UPLC system equipped with a Waters Xevo^TM^ G2 QTof MS. The metabolomic approaches, including sample and extraction and preparation, metabolite separation, mass spectrometry detection, data processing and metabolite identification, are carried out based on our reported methods.^23,24^

#### Validation cohort

The 200 serum and feces including 120 adult patients with stage 4-5 CKD and 80 age-matched healthy subjects were collected as the external validation cohort as described previously.^24^ Serum and fecal samples are collected from participants for metabolomic analyses.

#### Validation studies by animal models

To further verify the identification of renal function-associated serum metabolites and bacterial species, NX and UUO rat model is reproduced as described in our previous publications.^24,52^ The rats are randomized into the Sham and NX or Sham and UUO. NX and UUO operation are performed and the samples of serum and feces are collected for NX at week 12 and for UUO at week 1 for the following studies.

#### L. johnsonii culture and CRF rats treated by L. johnsonii

*L. johnsonii* (GDMCC1.730) is cultured with gut microbiota medium and DeMan, Rogosa and Sharpe broth overnight at 37 °C, respectively. The strain is kept under anaerobic conditions with an atmosphere of 80% N_2_, 10% CO_2_, and 10% H_2_. After centrifugation (3000 *rpm*, 10 min), the bacterial cells are washed twice with sterilized phosphate buffered saline (PBS) solution. Then, the bacteria are diluted to an optical density according to the OD_600_ value. Based on the previous publication,^36^ The rats are given the concentration of 10^9^ CFU of *L. johnsonii* in 0.2 mL of sterilized PBS by oral administration once everyday for 21 continuous days, and control and CRF groups are gavaged with 0.2 mL of sterilized PBS.

#### Validation studies by natural products

In order to demonstrate whether targeting *L. johnsonii* is a promising treatment to CKD, we test the effects of BSA and HMF on *L. johnsonii*. The rats are randomized into the healthy control, adenine-induced CRF, CRF + BSA and CRF + HMF groups (n = 8). Once daily, 10 mg/kg BSA and 10 mg/kg HMF are administered to CRF + BSA and CRF + HMF groups, respectively, by oral administration and all rats are killed after anaesthetised with 10% urethane at week 3. For clinical biochemical analysis, serum and 24-h urineare obtained, while the kidneys in the CRF rats are collected for following studies.

#### Renoprotective effect of IAld

To study the renoprotective effect of IAld, the rats are randomized into the healthy control, adenine-induced CRF, CRF+IAld (50 mg/kg) and CRF+IAld (100 mg/kg) groups (*n* = 6). Once daily, two doses of IAld are administered to the CRF rats, by oral gavage and all rats are killed after anaesthetised with 10% urethane at week 3. Creatinine and urea in the CRF rats are determined from serum samples, while the kidney and colon tissues are collected for the following studies. In addition, the rats are randomized into sham, UUO, UUO+IAld groups (*n* = 6). Once daily, 100 mg/kg IAld is administered to the CRF IAld group by oral gavage and all rats are killed after anaesthetised with 10% urethane at the 7th day. The obstructed kidney is collected for the following studies.

#### Cell viability analysis

HK-2 obtained from the American Type Culture Collection (Manassas, VA, USA) were cultured in DMEM/F-12 and supplemented with 10% FBS under 5% CO_2_ at 37 °C. HK-2 cells are treated with the different concentrations of IAld at different time points. Finally, the treated cells are used for further experiments. The absorbance at 450 nm is detected by using a microplate reader (Thermo Scientific, New York, USA). The cell viabilityis determined six times.

#### Antagonistic effect of IAld in the HP-stimulated HK-2 cells

To test the effect of IAld on AHR signaling, HK-2 cells are co-cultured with 10 nM HP and 60 or 120 μM IAld for 12 h to study IAld antagonistic effect on HP-stimulated AHR expression. 10 μM CH223191 is used as positive control.

#### Knockdown of AHR in the adenine-induced CRF mice and HP-stimulated HK-2 cells

We further assess the effects of IAld on adenine-induced CRF mice and HP-induced HK-2 cells. The delivery of adenovirus carrying shRNA against AHR or non-specific shRNA is performed as described in our previous publication.^52^ In addition, AHR knockdown in HP-stimulated HK-2 cells is carried out by using shRNA as described in our previous publications.^52,26^ AHR shRNA-transfected CRF mice and HP-stimulated HK-2 cells are treated with IAld. The effect of shRNA against AHR is detected by AHR mRNA expression. The samples are analyzed by Western blot analysis.

#### Physiological and biochemical data

SBP and DBP are measured as described in our previous publication.^52^ Kidney weight index is calculated based on ratio of kidney weight and body weight. Serum biochemistry parameters including creatinine, urea, uric acid, TC, triglyceride and LDL-C levels and urine creatinine levels are measured using an Olympus AU6402 automatic analyser. Routine blood parameters including RBC, white blood cells count (WBC), hemoglobin and platelets are determined by HF-3800 routine blood analyzer. CCr was calculated.

#### Light microscopic analysis

Light microscopy is performed by using paraffin-embedded biopsies stained with haematoxylin-eosin (H&E) and Masson’s trichrome staining as described in our previous publications.^26,52^

#### Immunohistochemical analysis

AHR, α-SMA and collagen I expressions are performed in kidney tissues based on previous reported method.^52^ All histological analyses are carried out by two investigators in a blinded manner.

#### Quantitative real-time polymerase chain reaction analysis

The specific primers including *AHR*, *CYP1A1*, *CYP1A2*, *CYP1B1* and *COX-2* are presented in our previous publications.^26,52^ The quantitative real-time polymerase chain reaction is performed in our previous publications.^53^ Gene expression is normalised by β-actin, and fold increases over control values are obtained by using 2^−DΔΔCt^ relative quantitative analysis.

#### Luciferase assay

The relative activity of AHR is tested in HK-2 cells by a dioxin-responsive element-driven luciferase reporter assay system kit. The experiments are performed in our previous publications.^52^

#### Western blot analysis

Cytoplasmic and nuclear proteins are extracted and protein expression is detected by using western blotting as described in our previous publication.^52^ The protein levels are normalized to glyceraldehyde-3-phosphate dehydrogenase (GAPDH), α-tubulin or histone H3. Specific bands are detected by ImageJ 1.48 v software.

#### Pathway analysis of significantly altered metabolites

Differential metabolites and metabolite enrichment are analyzed using 82 murine-related KEGG pathways. Both over-representation analysis based on the hypergeometric test and impact of metabolite alterations according to pathway topology by relative betweenness centrality measure were considered.^54^ Metabolites are mapped to the KEGG pathways by using Human Metabolome Database, which display significance compared to control group.

#### Statistical analysis

The number of replicates is 6-8/group, and the results are shown as mean ± SEM. Statistical analysis is conducted by GraphPad Prism and SPSS softwares. The significance of differences between two groups is performed by using unpaired Student’s *t*-test. Multiple groups are carried out by using one-way analysis of variance followed by a *post-hoc* test. In addition, the significance between two groups is also carried out by using Mann-Whitney U test. Some results were normalized to control. All tests were two-tailed. PCA and OPLS-DA of metabolites are carried out by using SIMCA-P software to cluster the plots between two groups. In addition, Spearman rank correlation is used for association analysis between each clinical parameter and each bacterium or between each serum metabolite and each bacterium in control and CRF groups. In some analyses*, P* values are corrected by using multiple comparisons based on Benjamini-Hochberg false discovery rate (FDR). *P* < 0.05 is considered statistical significant.

### Supplementary information

Supplementary Material

Original and and uncropped Western blots

## Data Availability

Most data relevant to the study are included in the article or uploaded as supplementary information. The raw data of RNA sequencing have been deposited, please find it in the National Center for Biotechnology Information database (No. SRP515910). Additional data are available upon reasonable request.
