# Supplementary material for: Targeting Lactobacillus johnsonii to reverse chronic kidney disease
Source: Signal Transduct Target Ther. 2024 Aug 5;9:195. doi: 10.1038/s41392-024-01913-1 (PMC11298530; doi:10.1038/s41392-024-01913-1)
Supplement: Supplementary file 1 — Supplementary Material [file 41392_2024_1913_MOESM1_ESM.docx]

**Supplementary Materials**

Targeting *Lactobacillus johnsonii* to reverse chronic kidney disease

Hua Miao, Fei Liu, Yan-Ni Wang, Xiao-Yong Yu, Shougang Zhuang, Yan Guo, Nosratola D. Vaziri, Shi-Xing Ma, Wei Su, You-Quan Shang, Ming Gao, Jin-Hua Zhang, Li Zhang, Ying-Yong Zhao, Gang Cao

Correspondence to: zhaoyybr@163.com

**This PDF file includes:**

Figures. S1 to S15

Tables S1 to S7


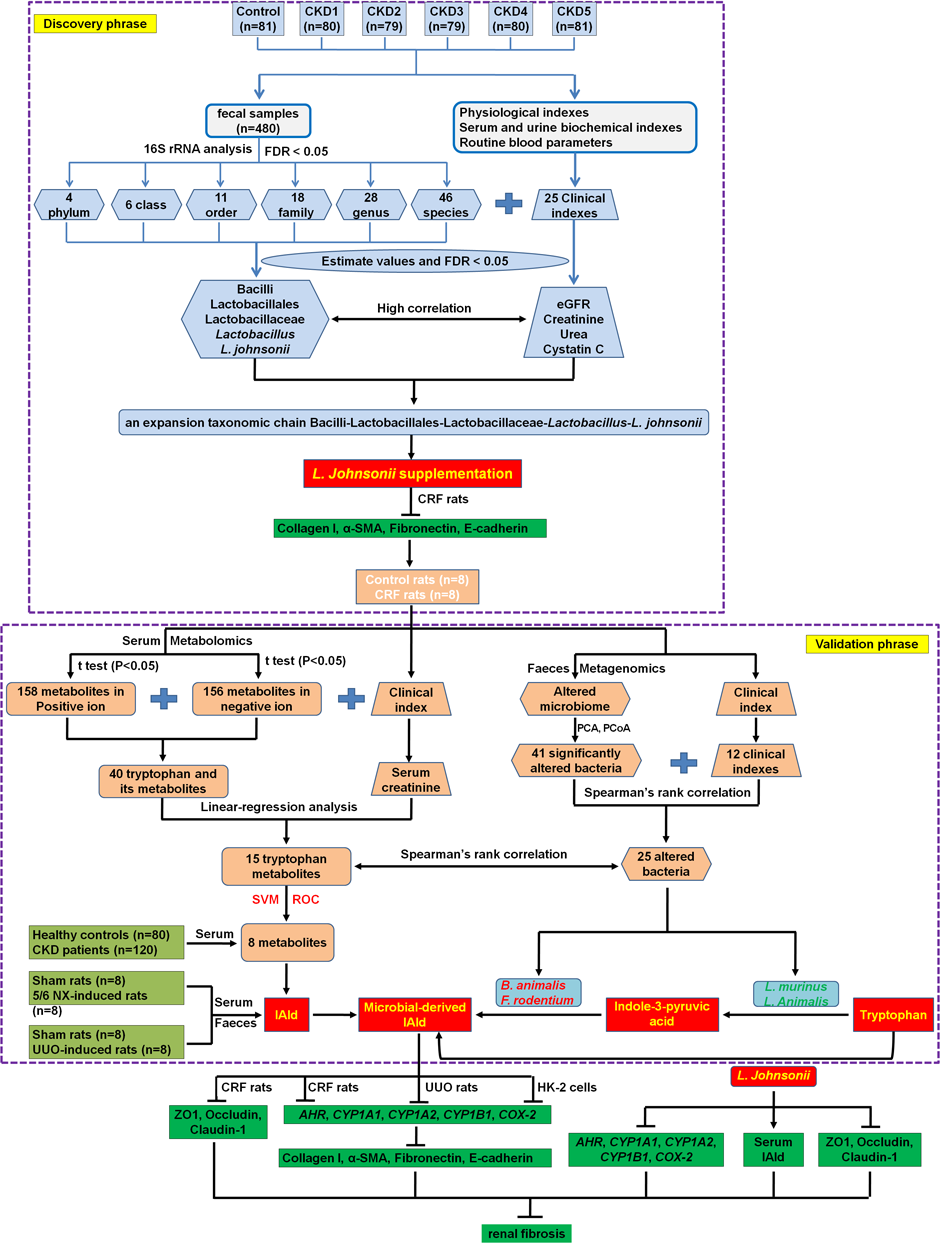


**Figure S1.**

**The overview of study design.** Flow diagram of microbiomic analysis presented significantly altered bacteria in the discovery phases by using 480 participants including patients with stage 1–5 CKD and healthy controls. The significant changed bacteria were selected at the levels of phylum, class, order, family, genus and species. The correlation was performed between significant changed bacteria and physiological indexes, serum and urine biochemical indexes and routine blood parameters. An elongation taxonomic chain Bacilli-Lactobacillales-Lactobacillaceae-*Lactobacillus*-*L. johnsonii* correlated with renal function decline in CKD progression, whose abundance strongly correlated with clinical renal function markers. *L. johnsonii* abundance reduced with progression of CKD and in CRF rats. *L. johnsonii* supplementation ameliorated renal injury and fibrosis. Serum IAld, whose level strongly negatively correlated with serum creatinine level in CRF rats, decreased in serum of rats induced by NX and UUO as well as advanced CKD patients. Treatment with IAld attenuates renal fibrosis, inhibits AHR signaling in rats with CRF or UUO, and in cultured 1-HP-stimulated HK-2 cells. Renoprotective effect of IAld is partially diminished in AHR deficiency mice and HK-2 cells. Our further results show that *L. johnsonii* supplementation retards renal fibrosis by inhibiting AHR signaling via increasing serum IAld level.


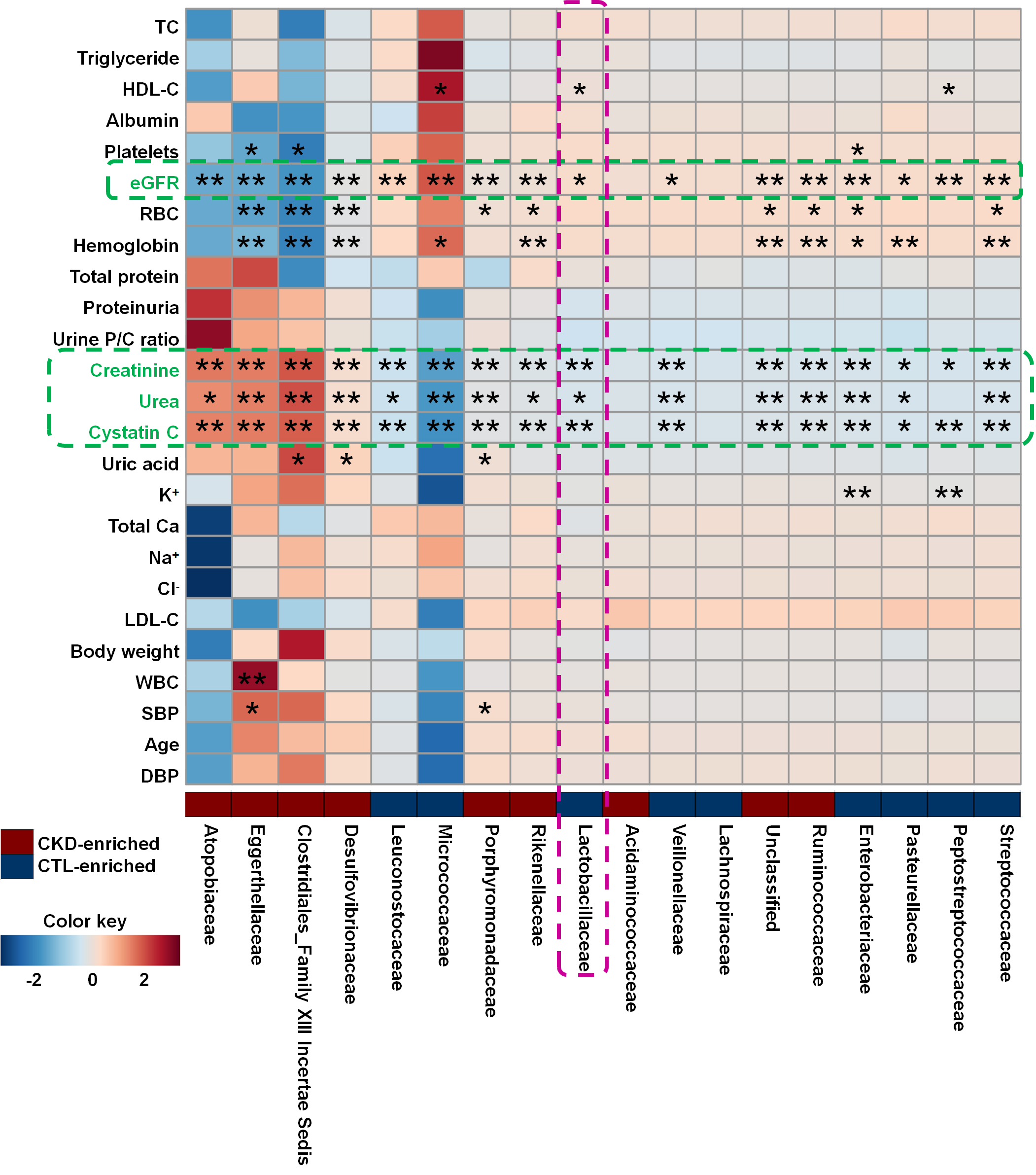


**Figure S2.**

**Associations between significantly changed bacteria at the family level and physiological and biochemical indexes.** Associations between 18 significantly changed bacteria at the family level and 25 physiological and biochemical indexes in patients with progressive CKD. ^*^*P* < 0.05; ^**^*P* < 0.01.


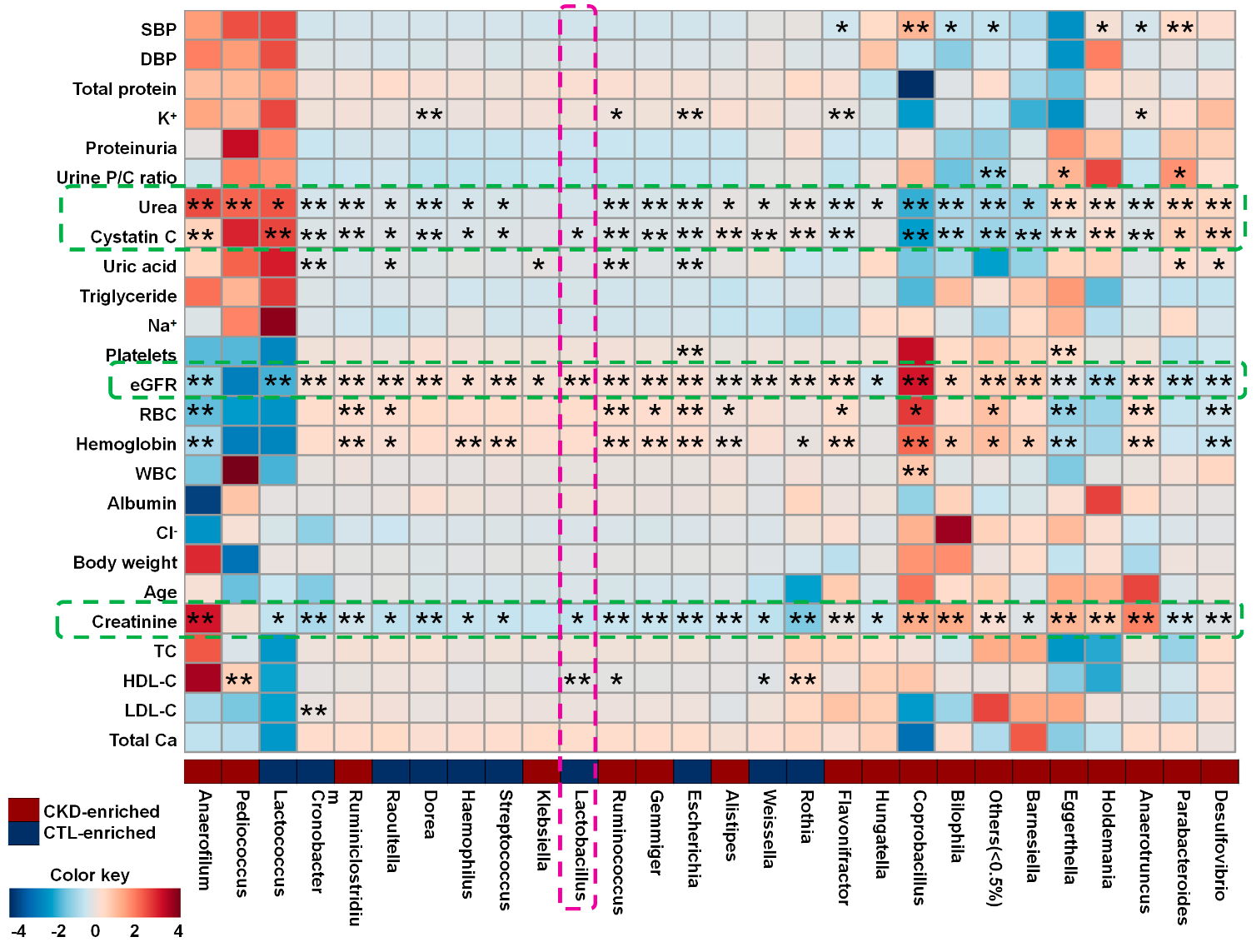


**Figure S3.**

**Associations between significantly changed bacteria at the genus level and physiological and biochemical indexes.** Associations between 28 significantly changed bacteria at the genus level and 25 physiological and biochemical indexes in patients with progressive CKD. ^*^*P* < 0.05; ^**^*P* < 0.01.





**Figure S4**.

**The relative abundances of significantly changed Bacilli, Lactobacillales and *L. ruminis*.** The relative abundances of Bacilli, Lactobacillales and *Lactobacillus ruminis* in healthy controls and five stages of patients with CKD. ^*^*P* < 0.05; ^**^*P* < 0.01 compared with healthy controls (control, n = 80/group; CKD1, n = 81/group; CKD2, n = 80/group; CKD3, n = 79/group; CKD4, n = 79/group; CKD5, n = 81/group). Data are represented as mean ± SEM.


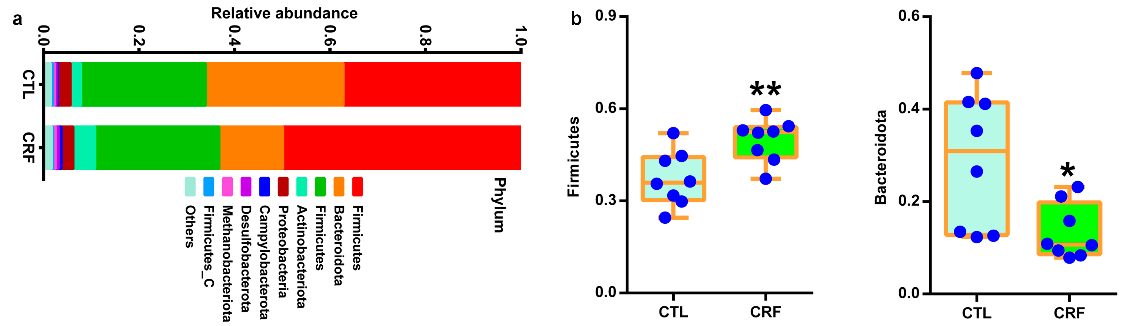


**Figure S5.**

**Altered gut microbiome profile in adenine-induced CRF rats.** **a** Taxonomic distribution of bacteria of two groups at the phylum level (top 10). **b** The abundance of Firmicutes and Bacteroidota of two groups. ^*^*P* < 0.05, ^**^*P* < 0.01 compared with control rats (n = 8/group).


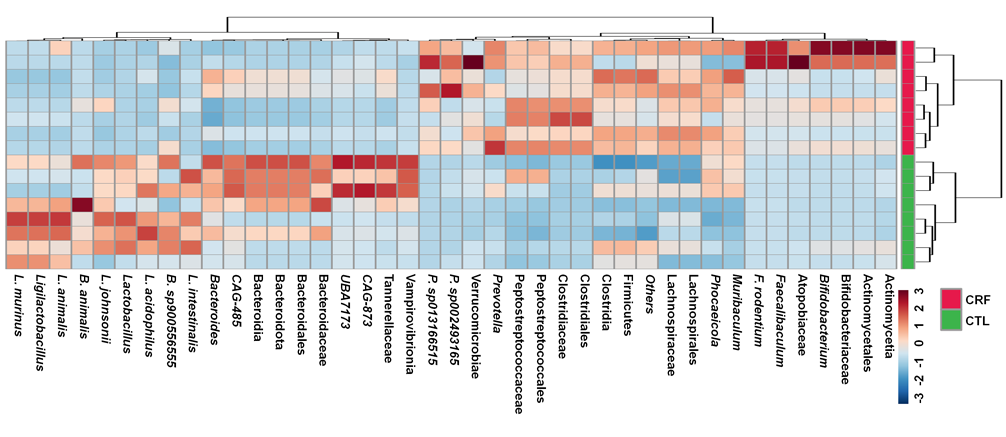


**Figure S6.**

**Significantly altered gut microbiota in feces of CRF rats.** Heatmap of 41 significantly altered bacteria from phylum, class, order, family, genus and species from fecal samples of control and adenine-induced CRF rats.


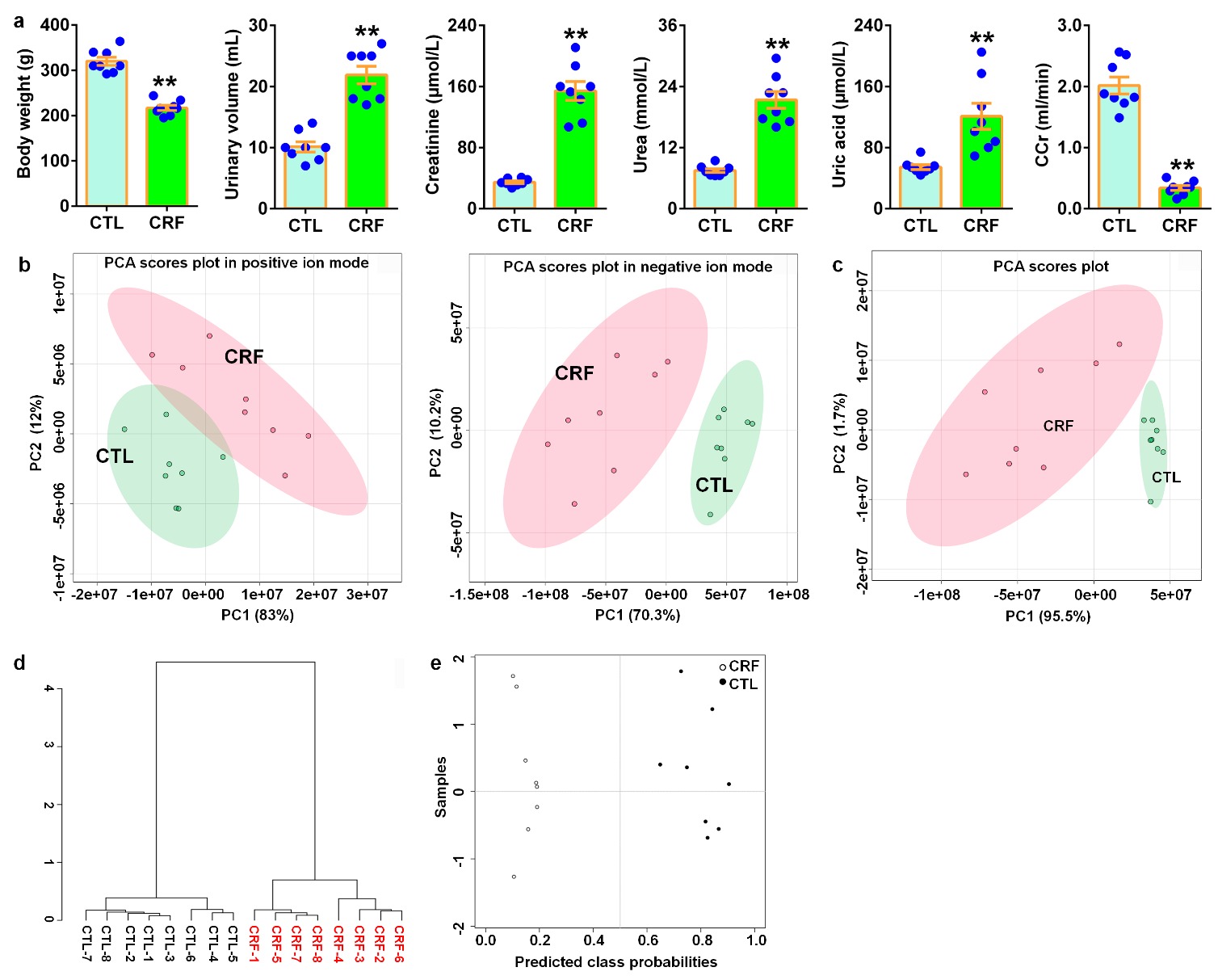


**Figure S7.**

**Altered serum metabolic profiles in CRF rats.** **a** Body weight and urinary volume as well as clinical serum biochemical indexes including creatinine, urea, uric acid and creatinine clearance rate (CCr) in control and CRF rats. **b** PCA score plots of 2278 variables from positive ion modes and 6030 variables from positive and negative ion modes in control and CRF rats. **c** PCA score plots of identified 314 significant altered metabolites in control and CRF rats. **d** Dendrogram of hierarchical clustering analysis based on 314 serum metabolites in control and CRF rats. **e** Diagnostic performances of 314 metabolites in control and CRF rats based on the based on the support vector machines method. ^**^*P* < 0.01 compared with control rats (n = 8/group). Data are represented as mean ± SEM.


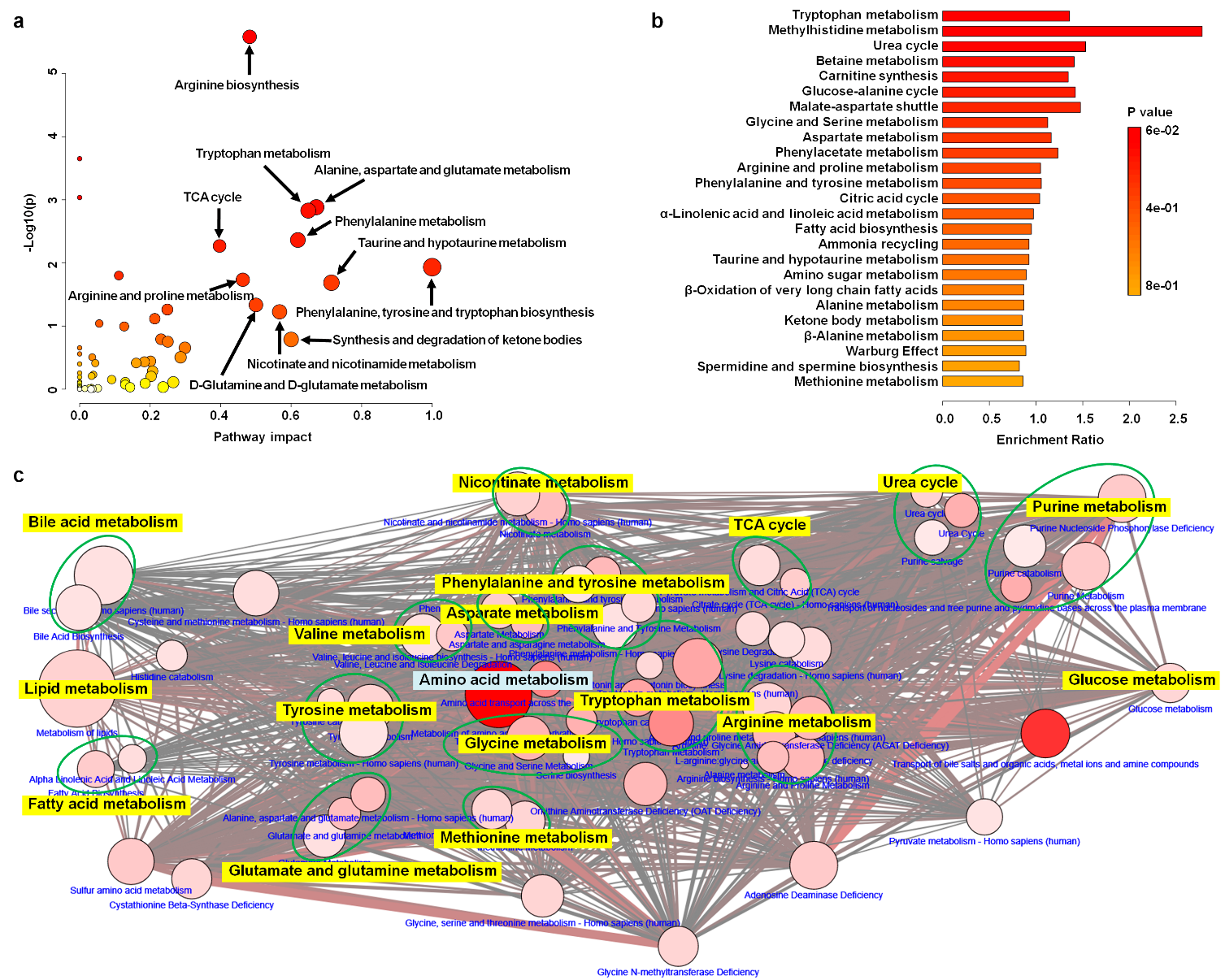


**Figure S8.**

**Metabolic pathway analysis of identified 314 important differential metabolites in CRF rats.** (a) Metabolic pathway analysis based on the KEGG metabolic library. Both the over-representation of changed metabolites within the pathway based on hypergeometric test and the impact of the altered metabolites on the function of the pathway via changes in critical junction points of the pathway showed by relative-betweeness centrality were evaluated. Results of each of 84 pathways in KEGG are simultaneously plotted to present the most significant pathways based on hypergeometric test p value presented by red shades and impact presented by circle diameter. (b) Enrichment analysis of 314 metabolites. (c) The over-representation analysis of metabolite pathway of identified 314 serum metabolites by using MetScape software running on cytoscape based on KEGG, Reactome and SMPDB database. AA, amino acids; AAD, amino acid and derivatives; CAD, carboxylic acids and derivatives; CCC, carbohydrates and carbohydrate conjugates; GP, glycerophospholipids; KAD, keto acids and derivatives; OSD, organic sulfonic acids and derivatives; PD, purines and derivatives; PDD, pyridines and derivatives; PMD, pyrimidines and derivatives; PMN, pyrimidine nucleosides; PN, purine nucleosides.


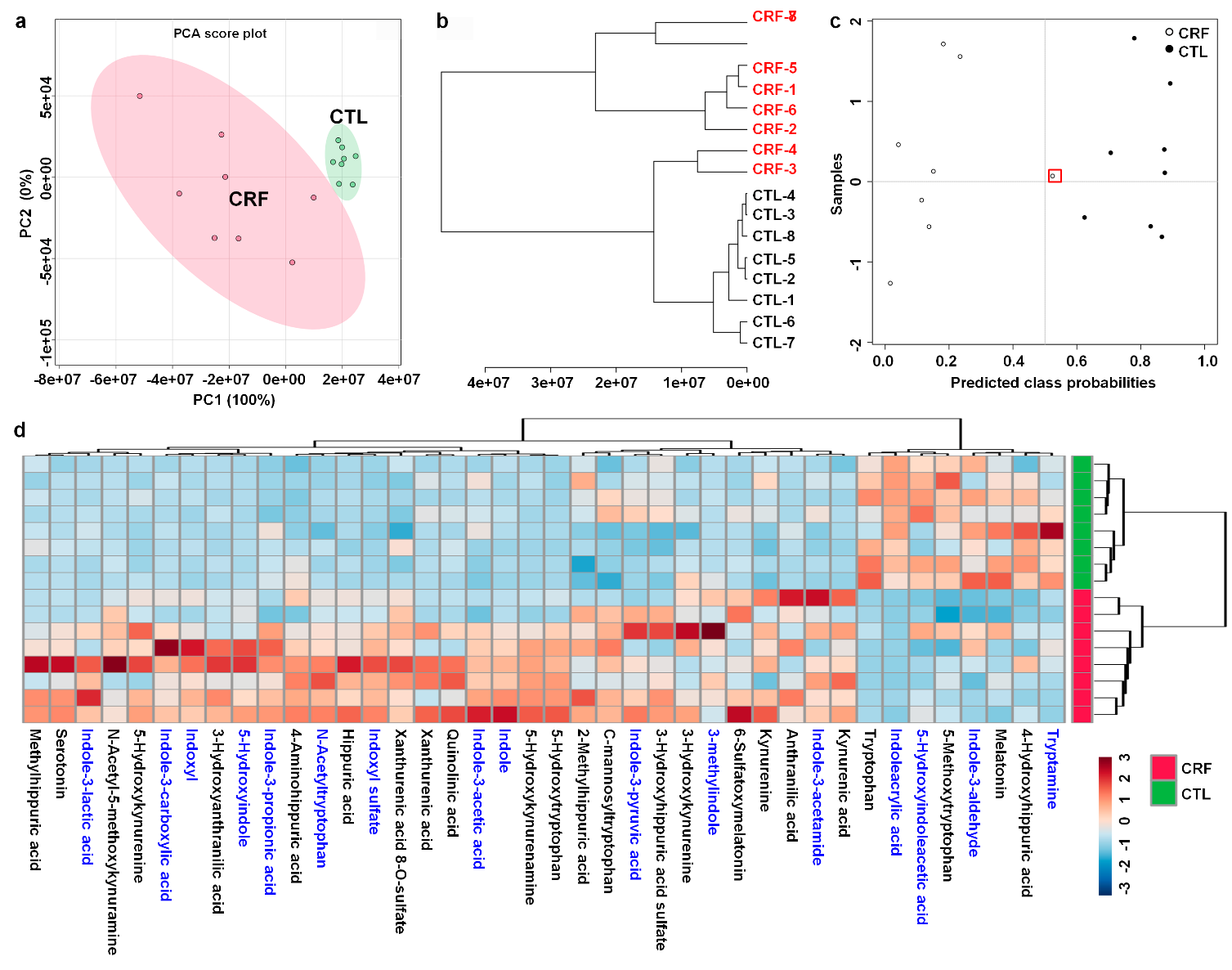


**Figure S9.**

**The perturbation of tryptophan metabolism plays a central role in CRF rats.** (a) PCA score plots of 40 tryptophan and its metabolites in control and CRF rats. (b) Dendrogram of hierarchical clustering analysis of 40 tryptophan and its metabolites in control and CRF rats. (c) Diagnostic performances of 40 tryptophan and its metabolites in control and CRF rats based on the support vector machines method. The black circles with red squares are for the incorrectly predicted samples in control group. (d) Heatmap of 40 tryptophan and its metabolites from host metabolism and gut microbiota metabolism between control and CRF rats. Red and blue in heatmap indicates increased and decreased levels, respectively. Metabolite names by black and red indicate tryptophan metabolites by host and gut microbiota metabolism, respectively.


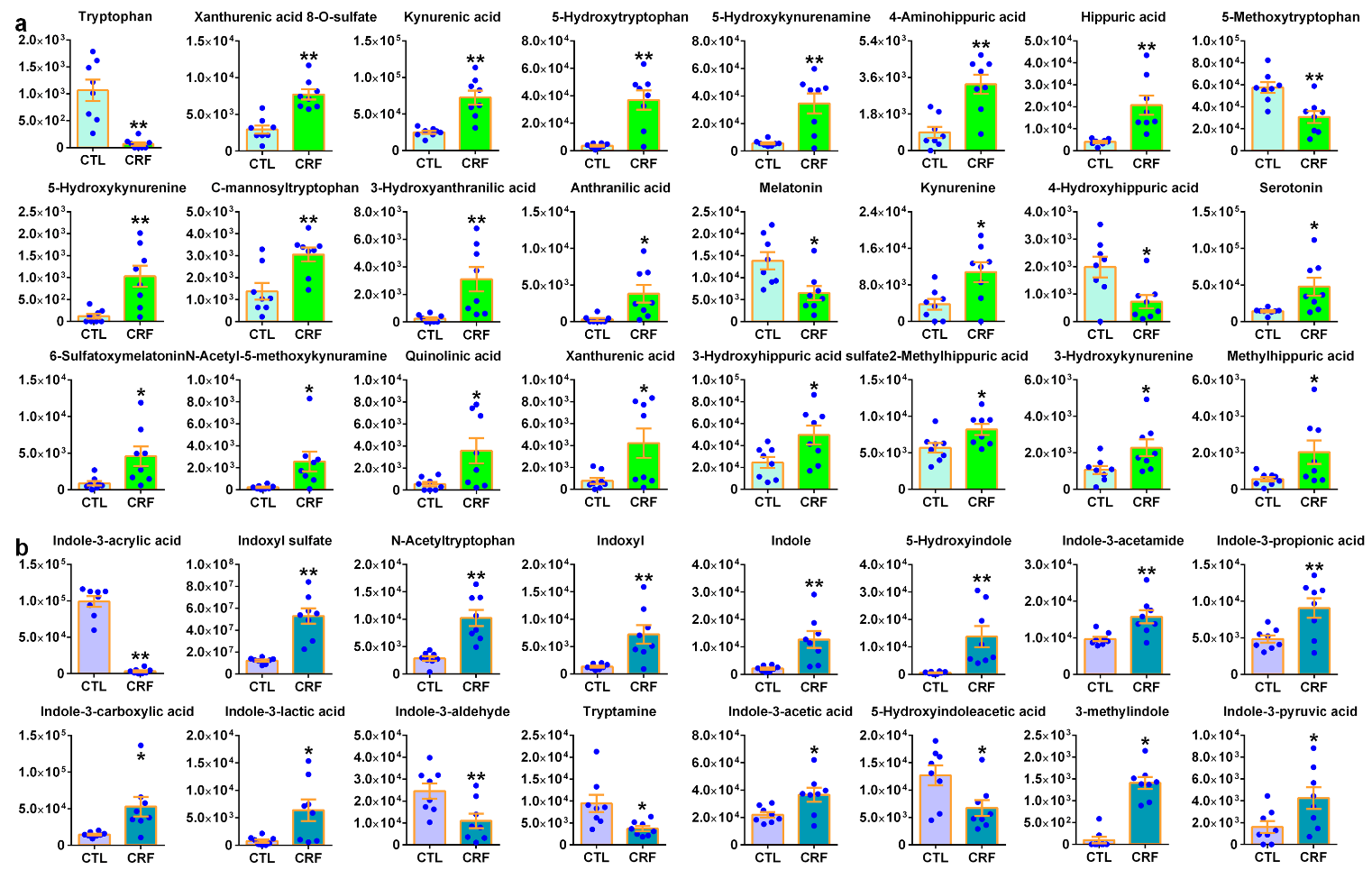


**Figure S10.**

**Altered tryptophan metabolism in CRF rats.** (a) Relative intensity of tryptophan and its 23 tryptophan metabolites by host metabolism between control and CRF rats. (b) Relative intensity of 16 tryptophan metabolites by gut microbiota metabolism between control and CRF rats. Abundance is represented as the relative intensity (y axis) of different groups (x axis). ^*^*P* < 0.05, ^**^*P* < 0.01 compared with control rats (n = 8/group). Data are represented as mean ± SEM.


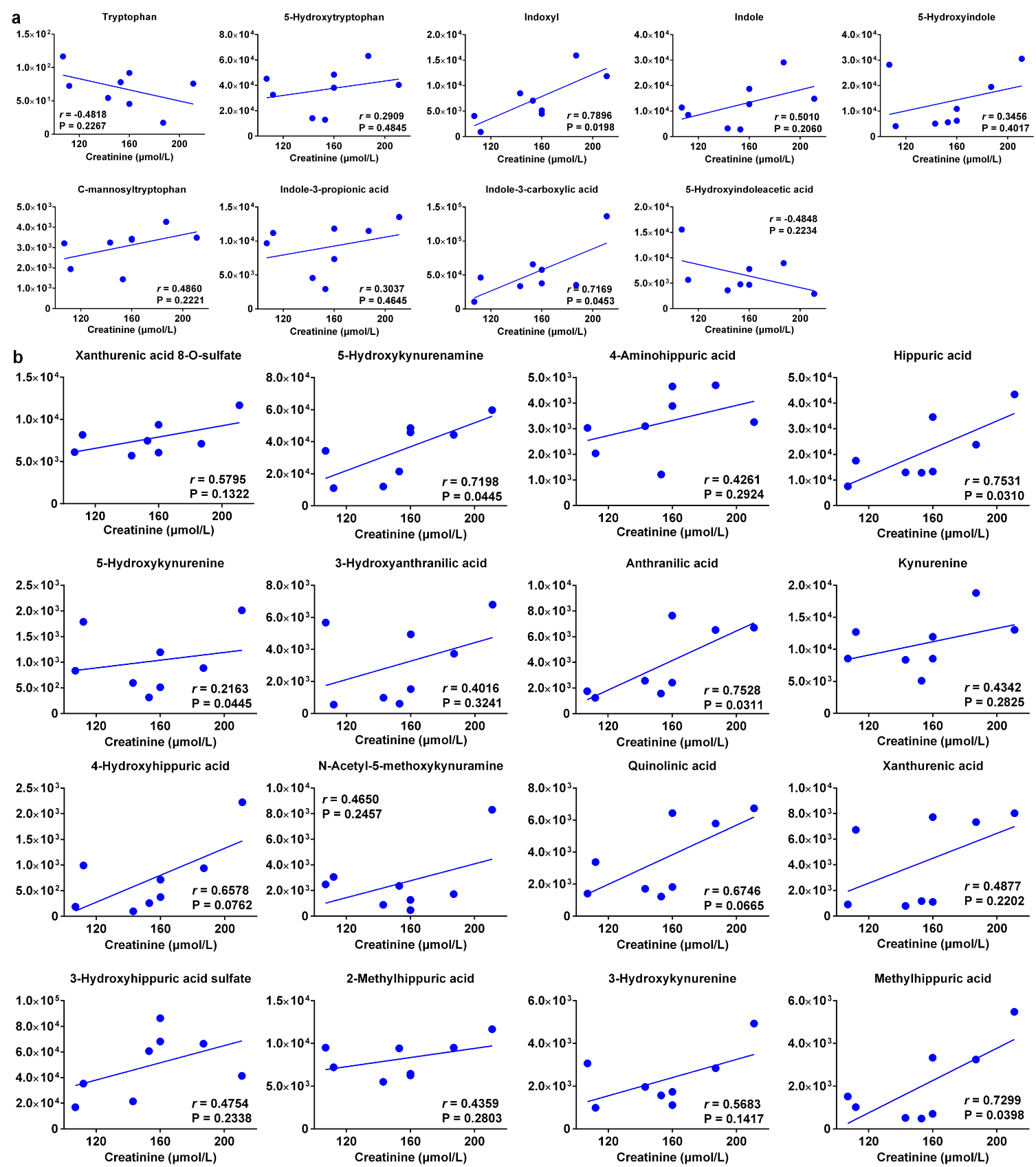


**Figure S11.**

**The associations between serum creatinine and tryptophan metabolites in CRF rats.** (a) The associations between creatinine and microbial-derived tryptophan metabolites in adenine-induced CRF rats. (b) The associations between creatinine and tryptophan metabolites by host in adenine-induced CRF rats. *r* indicated the linear coefficients.


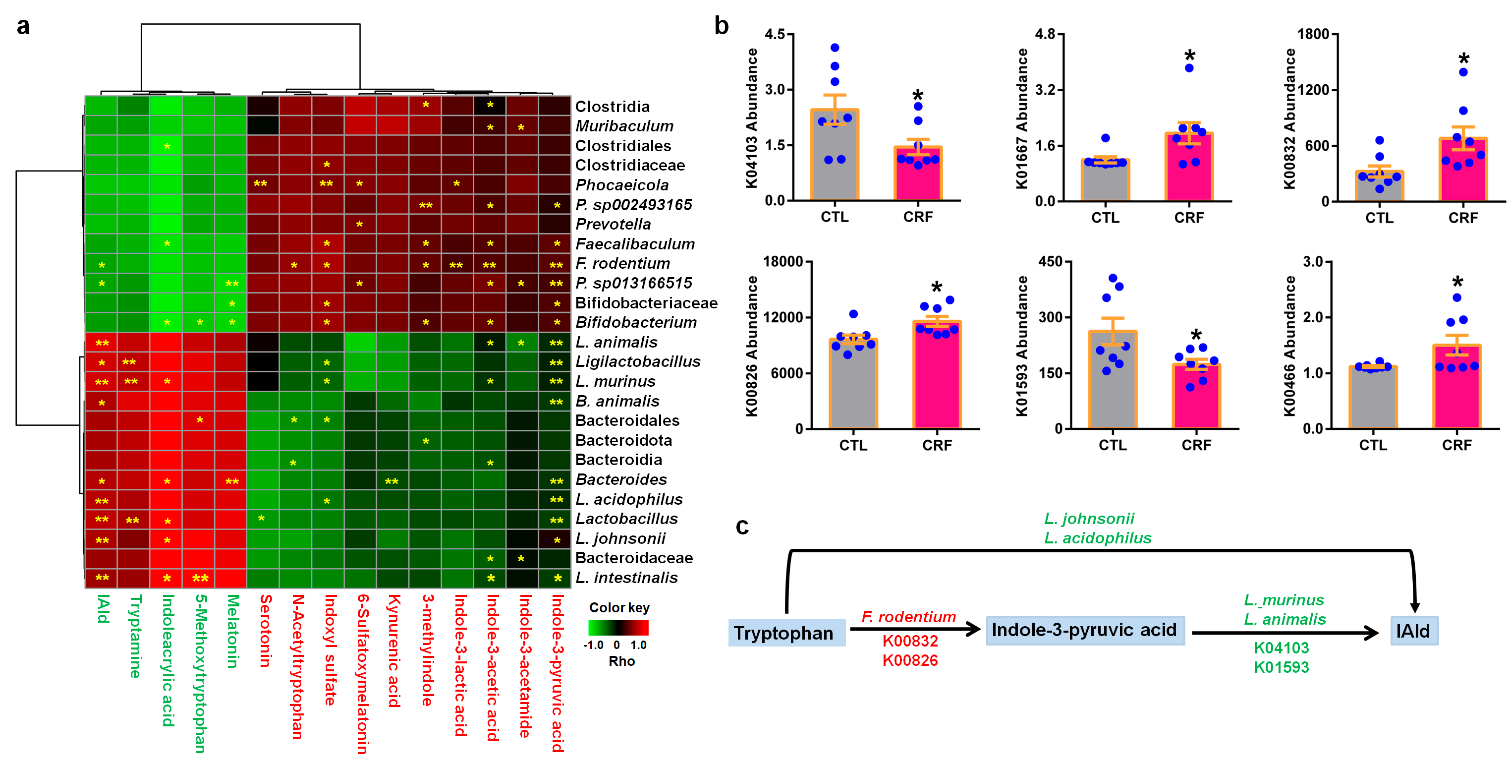


**Figure S12*.***

**Reduced *L. johnsonii* positively correlated with decreased serum IAld level in CRF rats.** (a) Heatmap of the Spearman’s rank correlation coefficient showing correlation between renal function-associated bacteria and creatinine-associated eight serum tryptophan metabolites. Bacteria or metabolites in green and red indicate control-enriched and CRF-enriched bacteria or metabolites, respectively. Rho in the color key represents the Spearman rank correlation coefficient. *^*^P* < 0.05; *^**^P* < 0.01. (b) The relative abundances of enzymes in KEGG pathway (n = 8/group). (c) Control-enriched (green) and CRF-enriched (red) microbial species associated with serum IAld levels and possessing genes involved in IAld generation in IpyA pathway. ^*^*P* < 0.05 compared with control rats.


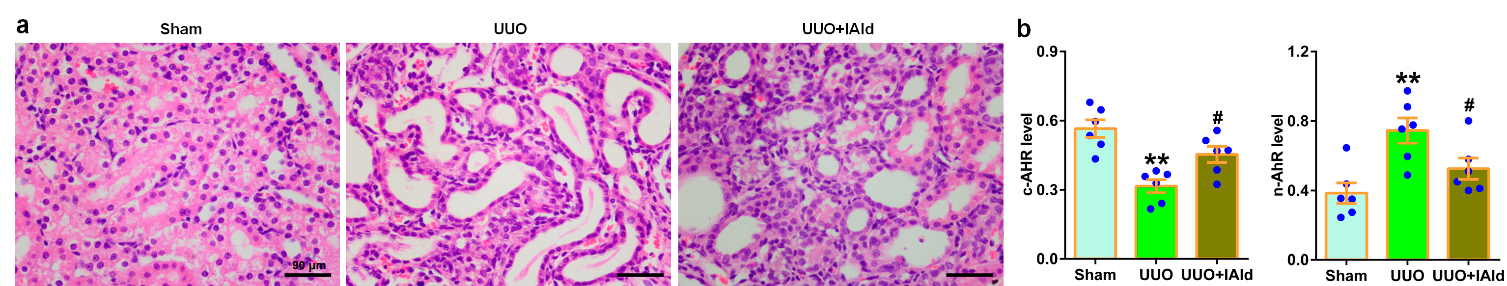


**Figure S13**.

**IAld treatment retarded renal injury and inhibited AHR signaling pathway.** (a) Images of H&E-stained kidney tissues in the UUO-induced rats treated with IAld. (b) Quantitative analysis of AHR expression in the nuclei and cytoplasm of kidney tissues in the UUO-induced rats treated with IAld. ^**^*P* < 0.01 compared with sham rats (n = 6/group). ^#^*P* < 0.05 compared with UO rats (n = 6/group). Data are represented as mean ± SEM.


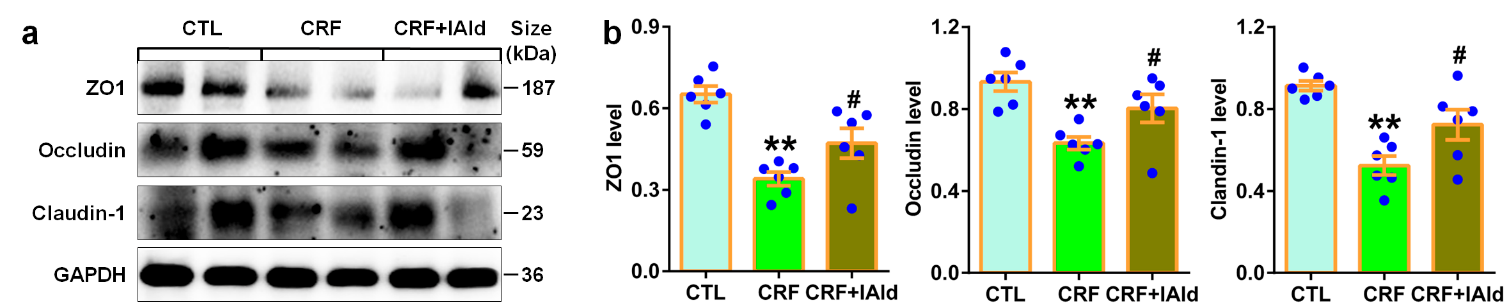


**Figure S14**.

**IAld treatment retarded colon injury in CRF rats.** (a) Protein expression of ZO1, occludin and claudin-1 of colon tissues in the different groups. (b) Quantitative analysis of ZO1, occludin and claudin-1 expression of colon tissues in the different groups. ^**^*P* < 0.01 compared with control rats (n = 6/group). ^#^*P* < 0.05 compared with CRF rats (n = 6/group). Data are represented as mean ± SEM.





**Figure S15.**

**Cell viability of IAld.** Cell viability after treatment with increasing concentrations of IAld (0–240μM) in HK-2 cells for 24 h and cell viability after treatment with 120-μM IAld at different time points (n = 6/group).

**Table S1.**

Summary of clinical and demographic baseline characteristics of healthy controls and patients with CKD

| Parameters | CTL | CKD1 | CKD2 | CKD3 | CKD4 | CKD5 | Estimate^a^ | Trend P ^b^ |
| --- | --- | --- | --- | --- | --- | --- | --- | --- |
| Sample size | 80 | 81 | 80 | 79 | 79 | 81 | - | - |
| Men (%) | 48.7 | 51.8 | 48.7 | 49.4 | 50.6 | 51.8 | 0.055 | 0.728 |
| Age (years) | 58.0±0.6 | 58.5±0.8 | 57.6±0.8 | 58.4±1.0 | 60.4±1.1 | 59.3±0.9 | 0.018 | 0.077 |
| Body weight (Kg) | 66.2±1.8 | 68.2±1.7 | 66.4±1.5 | 69.4±1.4 | 67.6±1.6 | 66.1±1.8 | 0.000 | 0.995 |
| SBP (mmHg) | 124.4±0.8 | 123.2±1.3 | 123±1.4 | 124.1±1.9 | 127.5±1.4 | 137.2±1.9^**^ | 0.033 | <0.01 |
| DBP (mmHg) | 77.2±1.2 | 75.7±0.7 | 75.6±1.2 | 75.2±1.3 | 78±0.8 | 81.4±1.1^*^ | 0.024 | <0.01 |
| eGFR (ml/min/1.73m^2^) | 108.2±1.8 | 106.1±2 | 73.1±1.1^**^ | 43.6±0.8^**^ | 24.2±0.6^**^ | 8.1±0.2^**^ | -0.040 | <0.01 |
| Creatinine (μmol/L) | 66.9±1.0 | 68.7±1.2 | 91.8±1.2^**^ | 143.9±2.1^**^ | 228.8±4.9^**^ | 569.4±16.0^**^ | 0.007 | <0.01 |
| Urea (mmol/L) | 5.2±0.2 | 5.2±0.2 | 6.0±0.3^*^ | 9.6±0.3^**^ | 12.4±0.4^**^ | 27.2±0.7^**^ | 0.157 | <0.01 |
| Cystatin C (mg/L) | 0.97±0.02 | 1.04±0.03 | 1.24±0.04^**^ | 2.1±0.08^**^ | 2.71±0.06^**^ | 4.5±0.12^**^ | 0.005 | <0.01 |
| Uric acid (μmol/L) | 364.4±8.0 | 372.2±9.4 | 382.1±8.4^**^ | 402.3±11.6^**^ | 439.9±11.3^**^ | 438.8±12.1^**^ | 1.029 | <0.01 |
| TC (mmol/L) | 4.52±0.15 | 4.89±0.15 | 4.41±0.25 | 4.77±0.19 | 4.14±0.11^*^ | 4.13±0.11^*^ | -0.143 | 0.056 |
| Triglyceride (mmol/L) | 1.43±0.06 | 1.33±0.11 | 1.41±0.09 | 1.31±0.08 | 1.73±0.09^**^ | 1.27±0.05^*^ | 0.044 | 0.678 |
| HDL-C (mmol/L) | 1.51±0.05 | 1.52±0.06 | 1.43±0.05 | 1.36±0.05^*^ | 1.37±0.04 | 1.39±0.05 | -0.121 | 0.051 |
| LDL-C (mmol/L) | 3.21±0.12 | 3.29±0.13 | 3.27±0.24 | 3.32±0.16 | 3.35±0.12 | 3.24±0.11 | 0.015 | 0.795 |
| Total protein (g/L) | 74±2.0 | 65.3±1.5^**^ | 60.6±1.6^**^ | 62.1±1.2^**^ | 63.3±1.1^**^ | 65.1±1.1^**^ | -0.023 | <0.01 |
| Albumin (g/L) | 48.1±1.6 | 34.2±1.2^**^ | 34.4±1.2^**^ | 36.5±0.9^**^ | 37.2±0.6^**^ | 35.9±0.9^**^ | -0.035 | <0.01 |
| K^+^ (mmol/L) | 3.8±0.06 | 4.05±0.05^**^ | 3.96±0.05^*^ | 4.26±0.05^**^ | 4.31±0.07^**^ | 4.73±0.07^**^ | 1.272 | <0.01 |
| Na^+^ (mmol/L) | 139.8±1.0 | 139.8±0.9 | 138.8±0.8 | 142.3±3.4 | 139.4±1.0 | 137.3±0.9 | -0.004 | 0.422 |
| Cl^-^ (mmol/L) | 103.5±0.8 | 104.4±0.7 | 104.3±0.7 | 104.9±0.7 | 105.5±0.8 | 104.7±0.7 | 0.018 | 0.126 |
| Total Ca (mmol/L) | 2.16±0.03 | 2.57±0.3 | 2.1±0.03 | 2.18±0.02 | 2.21±0.02 | 2.07±0.03^*^ | -0.099 | 0.157 |
| Proteinuria (g/24h) | 0.07±0.00 | 2.85±0.27^**^ | 1.85±0.22^**^ | 2.16±0.26^**^ | 2.03±0.21^**^ | 2.26±0.24^**^ | 0.159 | <0.01 |
| Urine P/C ratio | 0.15±0.00 | 3.98±0.39^**^ | 2.08±0.2^**^ | 2.49±0.25^**^ | 2.64±0.26^**^ | 2.73±0.26^**^ | 0.118 | <0.01 |
| WBC (10^9^/L) | 6.53±0.22 | 6.27±0.19 | 6.15±0.17 | 6.75±0.23 | 6.33±0.15 | 6.4±0.30 | 0.002 | 0.952 |
| RBC (10^12^/L) | 4.35±0.06 | 4.47±0.07 | 4.11±0.08^*^ | 4.18±0.07 | 3.71±0.07^**^ | 2.95±0.08^**^ | -1.138 | <0.01 |
| Hemoglobin (g/L) | 130.1±2.0 | 132.4±1.6 | 126.1±2.5 | 127.6±2.4 | 110.4±1.8^**^ | 90.6±1.9^**^ | -0.041 | <0.01 |
| Platelets (10^9^/L) | 215.6±6.4 | 215.1±9.1 | 195.6±7.7^*^ | 172.6±6.3^**^ | 175.8±6.2^**^ | 134.7±3.3^**^ | -0.010 | <0.01 |

Results are expressed as the means ± standard error, ^*^*P* < 0.05, ^**^*P* <0.01 compared with healthy controls. ^a^Changed tendency of each clinical biochemical index in CKD progression. Positive effect indicated that clinical biochemical index positively correlated with CKD progression; negative effect indicated that clinical biochemical index negatively correlated with CKD progression. ^b^Trend P value was computed using original linear regression.

**Table S2.**

The statistical analysis of phylum, class, order, family, genus and species in healthy controls and five stages of patients with CKD based on original linear regression.

| Level | Names | Estimate^a^ | Trend P^b^ | FDR^c^ |
| --- | --- | --- | --- | --- |
| Phylum | Candidatus Saccharibacteria | 2.73 | 6.51E-06 | 7.16E-05 |
| Phylum | Proteobacteria | -0.01 | 1.91E-03 | 5.26E-03 |
| Phylum | Tenericutes | 45.35 | 2.56E-05 | 1.41E-04 |
| Phylum | Unclassified | 0.06 | 1.27E-03 | 4.64E-03 |
| Class | Bacilli | -0.03 | 6.70E-03 | 1.90E-02 |
| Class | Clostridia | 0.01 | 2.18E-03 | 7.40E-03 |
| Class | Coriobacteriia | 2.96 | 1.69E-14 | 2.87E-13 |
| Class | Deltaproteobacteria | 0.80 | 1.79E-06 | 1.52E-05 |
| Class | Gammaproteobacteria | -0.01 | 2.13E-03 | 7.40E-03 |
| Class | Unclassified | 0.07 | 1.19E-04 | 6.72E-04 |
| Order | Acidaminococcales | 0.11 | 3.99E-03 | 1.64E-02 |
| Order | Clostridiales | 0.01 | 2.20E-03 | 1.10E-02 |
| Order | Coriobacteriales | 3.26 | 5.76E-03 | 1.64E-02 |
| Order | Desulfovibrionales | 0.80 | 1.79E-06 | 2.24E-05 |
| Order | Eggerthellales | 3.87 | 2.68E-14 | 6.71E-13 |
| Order | Enterobacteriales | -0.01 | 6.56E-03 | 1.64E-02 |
| Order | Lactobacillales | -0.03 | 6.63E-03 | 1.64E-02 |
| Order | Micrococcales | -3.56 | 8.01E-05 | 5.97E-04 |
| Order | Pasteurellales | -0.10 | 7.22E-03 | 1.64E-02 |
| Order | Veillonellales | -0.02 | 4.59E-03 | 1.64E-02 |
| Order | Unclassified | 0.07 | 9.55E-05 | 5.97E-04 |
| Family | Acidaminococcaceae | 0.11 | 3.99E-03 | 1.14E-02 |
| Family | Atopobiaceae | 3.74 | 2.16E-03 | 6.67E-03 |
| Family | Clostridiales Family XIII. Incertae Sedis | 4.76 | 9.00E-15 | 3.33E-13 |
| Family | Desulfovibrionaceae | 0.80 | 1.79E-06 | 1.66E-05 |
| Family | Eggerthellaceae | 3.87 | 2.68E-14 | 4.97E-13 |
| Family | Enterobacteriaceae | -0.01 | 6.56E-03 | 1.62E-02 |
| Family | Lachnospiraceae | -0.01 | 2.29E-02 | 4.72E-02 |
| Family | Lactobacillaceae | -0.12 | 9.37E-03 | 2.04E-02 |
| Family | Leuconostocaceae | -0.40 | 1.13E-03 | 3.78E-03 |
| Family | Micrococcaceae | -3.55 | 8.28E-05 | 3.40E-04 |
| Family | Pasteurellaceae | -0.10 | 7.22E-03 | 1.67E-02 |
| Family | Peptostreptococcaceae | -0.07 | 2.49E-05 | 1.15E-04 |
| Family | Porphyromonadaceae | 0.26 | 1.41E-04 | 5.23E-04 |
| Family | Rikenellaceae | 0.21 | 1.62E-05 | 9.96E-05 |
| Family | Ruminococcaceae | 0.02 | 1.39E-05 | 9.96E-05 |
| Family | Streptococcaceae | -0.09 | 2.40E-05 | 1.15E-04 |
| Family | Veillonellaceae | -0.02 | 4.59E-03 | 1.21E-02 |
| Family | Unclassified | 0.04 | 1.73E-06 | 1.66E-05 |
| Genus | *Escherichia* | -0.03 | 1.21E-08 | 1.45E-07 |
| Genus | *Gemmiger* | 0.02 | 1.81E-03 | 5.21E-03 |
| Genus | *Ruminococcus* | 0.10 | 8.19E-10 | 2.95E-08 |
| Genus | *Klebsiella* | 0.02 | 5.85E-04 | 2.11E-03 |
| Genus | *Streptococcus* | -0.08 | 3.21E-04 | 1.28E-03 |
| Genus | *Lactobacillus* | -0.20 | 1.97E-04 | 9.43E-04 |
| Genus | *Dorea* | -0.66 | 1.28E-09 | 3.08E-08 |
| Genus | *Haemophilus* | -0.10 | 6.79E-03 | 1.81E-02 |
| Genus | *Raoultella* | -0.36 | 8.82E-04 | 2.89E-03 |
| Genus | *Ruminiclostridium* | 0.50 | 4.81E-08 | 4.95E-07 |
| Genus | *Alistipes* | 0.21 | 1.62E-05 | 9.69E-05 |
| Genus | *Cronobacter* | -1.53 | 2.35E-04 | 9.94E-04 |
| Genus | *Others (<0.5%)* | 1.93 | 1.08E-05 | 7.09E-05 |
| Genus | *Parabacteroides* | 0.29 | 9.96E-04 | 3.12E-03 |
| Genus | *Weissella* | -0.48 | 7.85E-04 | 2.69E-03 |
| Genus | *Rothia* | -3.54 | 8.80E-05 | 4.87E-04 |
| Genus | *Barnesiella* | 1.15 | 1.65E-03 | 4.94E-03 |
| Genus | *Flavonifractor* | 1.54 | 3.05E-06 | 2.75E-05 |
| Genus | *Desulfovibrio* | 0.81 | 4.50E-06 | 3.60E-05 |
| Genus | *Hungatella* | 0.17 | 4.82E-03 | 1.33E-02 |
| Genus | *Holdemania* | 4.29 | 2.23E-04 | 9.94E-04 |
| Genus | *Bilophila* | 5.97 | 1.05E-04 | 5.42E-04 |
| Genus | *Lactococcus* | -0.30 | 5.79E-04 | 2.11E-03 |
| Genus | *Eggerthella* | 6.31 | 6.06E-12 | 4.36E-10 |
| Genus | *Anaerotruncus* | 8.26 | 1.18E-08 | 1.45E-07 |
| Genus | *Coprobacillus* | 7.15 | 1.21E-08 | 1.45E-07 |
| Genus | *Pediococcus* | 3.35 | 1.88E-02 | 4.83E-02 |
| Genus | *Anaerofilum* | 11.20 | 9.87E-06 | 7.09E-05 |
| Species | *Alistipes finegoldii DSM 17242* | 0.34 | 1.73E-04 | 9.57E-04 |
| Species | *Alistipes indistinctus YIT 12060* | 2.07 | 1.91E-04 | 1.02E-03 |
| Species | *Alistipes onderdonkii* | 0.41 | 7.69E-04 | 3.30E-03 |
| Species | *Anaerostipes caccae* | 1.06 | 1.07E-02 | 3.55E-02 |
| Species | *Anaerotruncus colihominis DSM 17241* | 8.26 | 1.18E-08 | 2.01E-07 |
| Species | *Bacteroides eggerthii DSM 20697* | 0.97 | 1.04E-04 | 6.16E-04 |
| Species | *Bacteroides ovatus* | 0.23 | 3.86E-05 | 3.02E-04 |
| Species | *Bacteroides plebeius DSM 17135* | -0.20 | 7.05E-04 | 3.13E-03 |
| Species | *Barnesiella intestinihominis YIT 11860* | 1.16 | 1.39E-03 | 5.62E-03 |
| Species | *Bilophila wadsworthia* | 5.97 | 1.05E-04 | 6.16E-04 |
| Species | *Blautia luti* | -0.16 | 1.69E-05 | 1.41E-04 |
| Species | *Blautia schinkii* | 2.08 | 1.07E-05 | 9.45E-05 |
| Species | *Clostridium perfringens ATCC 13124* | -0.57 | 1.53E-02 | 4.52E-02 |
| Species | *Coprobacillus cateniformis JCM 10604* | 7.15 | 1.21E-08 | 2.01E-07 |
| Species | *Coprococcus eutactus* | 0.17 | 1.52E-02 | 4.52E-02 |
| Species | *Cronobacter malonaticus* | -1.53 | 2.35E-04 | 1.16E-03 |
| Species | *Dorea longicatena* | -0.66 | 1.28E-09 | 3.41E-08 |
| Species | *Eggerthella lenta DSM 2243* | 6.31 | 6.06E-12 | 2.73E-10 |
| Species | *Erysipelatoclostridium ramosum DSM 1402* | 0.41 | 1.99E-03 | 7.37E-03 |
| Species | *Eubacterium coprostanoligenes* | 0.15 | 7.87E-11 | 2.62E-09 |
| Species | *Flavonifractor plautii* | 1.56 | 5.13E-06 | 4.87E-05 |
| Species | *Gemmiger formicilis* | 0.02 | 1.81E-03 | 6.88E-03 |
| Species | *Haemophilus parainfluenzae ATCC 33392* | -0.11 | 5.87E-03 | 2.00E-02 |
| Species | *Holdemania filiformis* | 4.29 | 2.23E-04 | 1.14E-03 |
| Species | *Hungatella hathewayi* | 0.17 | 4.82E-03 | 1.73E-02 |
| Species | *Klebsiella pneumoniae* | 0.02 | 5.85E-04 | 2.78E-03 |
| Species | *Lactobacillus johnsonii* | -0.12 | 3.96E-02 | 1.51E-02 |
| Species | *Lactobacillus ruminis* | -0.76 | 8.52E-05 | 5.67E-04 |
| Species | *Megamonas funiformis YIT 11815* | -1.35 | 6.92E-05 | 5.11E-04 |
| Species | *Parabacteroides distasonis* | 0.60 | 1.33E-02 | 4.21E-02 |
| Species | *Parabacteroides goldsteinii* | 0.74 | 1.45E-02 | 4.49E-02 |
| Species | *Rothia mucilaginosa* | -3.70 | 7.41E-05 | 5.19E-04 |
| Species | *Ruminococcus bromii* | 0.12 | 6.15E-12 | 2.73E-10 |
| Species | *Ruminococcus callidus* | -0.76 | 5.34E-03 | 1.87E-02 |
| Species | *Ruminococcus faecis JCM 15917* | -0.19 | 1.23E-03 | 5.09E-03 |
| Species | *Streptococcus lutetiensis* | -0.32 | 1.11E-08 | 2.01E-07 |
| Species | *TM7 phylum sp. oral clone DR034* | 2.58 | 1.51E-03 | 5.91E-03 |
| Species | *Weissella cibaria* | -0.51 | 6.96E-04 | 3.13E-03 |
| Species | *[Clostridium] bolteae* | 0.72 | 5.45E-07 | 6.59E-06 |
| Species | *[Clostridium] colinum* | -1.63 | 2.02E-06 | 2.06E-05 |
| Species | *[Clostridium] scindens* | 2.48 | 2.91E-08 | 4.30E-07 |
| Species | *[Clostridium] spiroforme DSM 1552* | 8.60 | 3.42E-14 | 4.55E-12 |
| Species | *[Eubacterium] hallii* | -0.16 | 2.37E-07 | 3.15E-06 |
| Species | *[Eubacterium] tortuosum* | 3.04 | 1.27E-02 | 4.11E-02 |
| Species | *Unclassified* | -0.01 | 1.07E-04 | 6.16E-04 |
| Species | *Others (<0.5%)* | 1.50 | 1.87E-06 | 2.06E-05 |

^a^Positive effect indicates that the relative abundance of gut bacteria is positively associated with CKD stage; negative effect indicates that the relative abundance of gut bacteria is negatively associated with CKD stage. ^b^Trend P value was computed using original linear regression. ^c^ FDR value was obtained from the adjusted *P* value using the Benjamini Hochberg method.

**Table S3.**

Identification of differential metabolites in serum of control and adenine-induced CRF rats

| Metabolites | FC^a^ | P^b^ | P^c^ | FDR^d^ | | Ion mode | | Class | |  |
| --- | --- | --- | --- | --- | --- | --- | --- | --- | --- | --- |
| Indole-3-acrylic acid | 0.03 | 2.5E-09 | 1.6E-04 | | 7.8E-07 | | Pos | | TD | |
| Indoxyl sulfate | 4.35 | 4.9E-05 | 1.6E-04 | | 6.2E-04 | | Neg | | TD | |
| Xanthurenic acid 8-O-sulfate | 2.63 | 1.1E-04 | 3.1E-04 | | 9.8E-04 | | Pos | | TD | |
| Tryptophan | 0.06 | 1.9E-04 | 1.6E-04 | | 1.2E-03 | | Pos | | TD | |
| Kynurenic acid | 2.91 | 2.4E-04 | 3.1E-04 | | 1.5E-03 | | Pos | | TD | |
| N-Acetyltryptophan | 3.61 | 2.6E-04 | 1.6E-04 | | 1.6E-03 | | Pos | | TD | |
| 5-Hydroxytryptophan | 10.27 | 3.1E-04 | 3.0E-03 | | 1.8E-03 | | Pos | | TD | |
| 5-Hydroxykynurenamine | 6.13 | 1.4E-03 | 1.0E-02 | | 4.2E-03 | | Pos | | TD | |
| 4-Aminohippuric acid | 3.63 | 1.7E-03 | 1.0E-02 | | 4.8E-03 | | Pos | | TD | |
| Hippuric acid | 5.19 | 2.0E-03 | 1.6E-04 | | 5.3E-03 | | Pos | | TD | |
| 5-Methoxytryptophan | 0.53 | 2.5E-03 | 7.0E-03 | | 6.3E-03 | | Pos | | TD | |
| 5-Hydroxykynurenine | 8.43 | 3.5E-03 | 7.0E-03 | | 8.1E-03 | | Pos | | TD | |
| Indoxyl | 5.45 | 3.7E-03 | 4.7E-03 | | 8.3E-03 | | Pos | | TD | |
| Indole | 6.10 | 3.8E-03 | 1.9E-03 | | 8.3E-03 | | Pos | | TD | |
| 5-Hydroxyindole | 22.78 | 4.1E-03 | 1.6E-04 | | 8.7E-03 | | Pos | | TD | |
| C-mannosyltryptophan | 2.22 | 4.8E-03 | 4.7E-03 | | 9.4E-03 | | Neg | | TD | |
| 3-Hydroxyanthranilic acid | 13.12 | 6.1E-03 | 6.2E-04 | | 1.1E-02 | | Neg | | TD | |
| Indole-3-acetamide | 1.63 | 6.8E-03 | 7.0E-03 | | 1.2E-02 | | Pos | | TD | |
| Indole-3-propionic acid | 1.88 | 9.8E-03 | 3.8E-02 | | 1.6E-02 | | Neg | | TD | |
| Anthranilic acid | 12.98 | 1.2E-02 | 1.9E-03 | | 1.8E-02 | | Pos | | TD | |
| Melatonin | 0.47 | 1.2E-02 | 7.0E-03 | | 1.8E-02 | | Pos | | TD | |
| Kynurenine | 2.88 | 1.2E-02 | 2.1E-02 | | 1.9E-02 | | Pos | | TD | |
| Indole-3-carboxylic acid | 3.59 | 1.3E-02 | 7.0E-03 | | 1.9E-02 | | Pos | | TD | |
| Indole-3-lactic acid | 8.33 | 1.4E-02 | 2.8E-02 | | 2.0E-02 | | Pos | | TD | |
| Indole-3-aldehyde | 0.44 | 1.4E-02 | 1.5E-02 | | 2.0E-02 | | Pos | | TD | |
| Tryptamine | 0.39 | 1.4E-02 | 4.7E-03 | | 2.1E-02 | | Pos | | TD | |
| 4-Hydroxyhippuric acid | 0.36 | 1.4E-02 | 2.8E-02 | | 2.1E-02 | | Pos | | TD | |
| Serotonin | 3.38 | 1.5E-02 | 1.0E-02 | | 2.1E-02 | | Pos | | TD | |
| Indole-3-acetic acid | 1.66 | 1.9E-02 | 3.8E-02 | | 2.6E-02 | | Pos | | TD | |
| 6-Sulfatoxymelatonin | 5.29 | 1.9E-02 | 4.7E-03 | | 2.6E-02 | | Pos | | TD | |
| N-Acetyl-5-methoxykynuramine | 11.86 | 2.0E-02 | 7.0E-03 | | 2.7E-02 | | Neg | | TD | |
| Quinolinic acid | 6.69 | 2.2E-02 | 3.8E-02 | | 2.9E-02 | | Pos | | TD | |
| 5-Hydroxyindoleacetic acid | 0.53 | 2.3E-02 | 3.8E-02 | | 3.0E-02 | | Neg | | TD | |
| Xanthurenic acid | 5.40 | 2.4E-02 | 3.8E-02 | | 3.0E-02 | | Pos | | TD | |
| 3-Hydroxyhippuric acid sulfate | 2.02 | 2.6E-02 | 6.5E-02 | | 3.2E-02 | | Neg | | TD | |
| 2-Methylhippuric acid | 1.44 | 2.7E-02 | 2.1E-02 | | 3.3E-02 | | Pos | | TD | |
| 3-methylindole | 13.87 | 3.3E-02 | 1.1E-03 | | 3.8E-02 | | Neg | | TD | |
| 3-Hydroxykynurenine | 2.14 | 3.3E-02 | 2.8E-02 | | 3.8E-02 | | Neg | | TD | |
| Indole-3-pyruvic acid | 2.65 | 3.4E-02 | 5.0E-02 | | 3.9E-02 | | Neg | | TD | |
| Methylhippuric acid | 3.66 | 3.9E-02 | 3.8E-02 | | 4.3E-02 | | Pos | | TD | |
| Cysteate | 8.76 | 2.4E-05 | 1.6E-04 | | 4.5E-04 | | Neg | | AD | |
| Homoarginine | 12.11 | 3.2E-05 | 1.6E-04 | | 5.6E-04 | | Neg | | AD | |
| Valine | 0.65 | 3.6E-05 | 1.6E-04 | | 5.4E-04 | | Pos | | AD | |
| γ-Glutamylleucine | 2.55 | 4.2E-05 | 1.6E-04 | | 5.8E-04 | | Neg | | AD | |
| N-Acetyl-L-aspartic acid | 5.34 | 4.5E-05 | 3.1E-04 | | 5.9E-04 | | Neg | | AD | |
| Taurine | 0.12 | 5.5E-05 | 1.6E-04 | | 6.4E-04 | | Pos | | AD | |
| Phenylacetylglycine | 2.34 | 5.6E-05 | 1.6E-04 | | 6.3E-04 | | Neg | | AD | |
| N6,N6,N6-Trimethyllysine | 14.19 | 6.3E-05 | 1.6E-04 | | 6.4E-04 | | Neg | | AD | |
| Betaine | 0.67 | 1.2E-04 | 3.1E-04 | | 1.0E-03 | | Pos | | AD | |
| Phenylalanine | 6.37 | 1.3E-04 | 6.2E-04 | | 1.1E-03 | | Pos | | AD | |
| Sarcosine | 4.83 | 1.5E-04 | 1.6E-04 | | 1.1E-03 | | Neg | | AD | |
| Aspartic acid | 12.16 | 1.8E-04 | 1.6E-04 | | 1.3E-03 | | Pos | | AD | |
| γ-Glutamylisoleucine | 10.11 | 1.9E-04 | 1.6E-04 | | 1.3E-03 | | Neg | | AD | |
| Pipecolate | 5.22 | 3.8E-04 | 1.6E-04 | | 1.9E-03 | | Pos | | AD | |
| Lysine | 0.33 | 5.0E-04 | 3.1E-04 | | 2.3E-03 | | Pos | | AD | |
| γ-Glutamylglutamine | 21.13 | 6.3E-04 | 1.6E-04 | | 2.6E-03 | | Neg | | AD | |
| Betonicine | 3.17 | 6.9E-04 | 1.6E-04 | | 2.7E-03 | | Neg | | AD | |
| O-Sulfo-L-tyrosine | 5.24 | 7.9E-04 | 4.7E-03 | | 3.0E-03 | | Neg | | AD | |
| Methionine | 0.48 | 8.1E-04 | 1.9E-03 | | 3.0E-03 | | Pos | | AD | |
| Allantoate | 5.05 | 8.4E-04 | 7.0E-03 | | 3.0E-03 | | Neg | | AD | |
| Threonine | 0.51 | 8.8E-04 | 1.9E-03 | | 3.1E-03 | | Pos | | AD | |
| Tyrosine | 0.62 | 9.0E-04 | 1.9E-03 | | 3.1E-03 | | Pos | | AD | |
| N-ε-Acetyllysine | 2.84 | 1.9E-03 | 4.7E-03 | | 5.2E-03 | | Neg | | AD | |
| 5-Oxoproline | 5.90 | 2.1E-03 | 1.1E-03 | | 5.5E-03 | | Pos | | AD | |
| Serine | 0.54 | 2.8E-03 | 3.0E-03 | | 6.8E-03 | | Pos | | AD | |
| α-Aminoadipate | 0.49 | 3.0E-03 | 4.7E-03 | | 7.4E-03 | | Pos | | AD | |
| S-Adenosylmethionine | 3.55 | 3.2E-03 | 7.0E-03 | | 7.6E-03 | | Neg | | AD | |
| 1-Methylhistidine | 4.11 | 4.1E-03 | 1.5E-02 | | 8.8E-03 | | Neg | | AD | |
| Homocysteic acid | 1.81 | 4.2E-03 | 2.8E-02 | | 8.8E-03 | | Pos | | AD | |
| Proline betaine | 5.97 | 4.2E-03 | 6.2E-04 | | 8.8E-03 | | Pos | | AD | |
| Glutamine | 6.29 | 4.4E-03 | 1.6E-04 | | 9.0E-03 | | Pos | | AD | |
| Homocystine | 24.21 | 4.5E-03 | 1.6E-04 | | 9.0E-03 | | Neg | | AD | |
| N-Butyrylglycine | 1.55 | 4.7E-03 | 1.6E-04 | | 9.4E-03 | | Neg | | AD | |
| 3-Methylhistidine | 26.80 | 5.0E-03 | 4.7E-03 | | 9.6E-03 | | Neg | | AD | |
| Arginine | 0.38 | 5.6E-03 | 1.0E-02 | | 1.1E-02 | | Neg | | AD | |
| Acetylalanine | 9.95 | 5.6E-03 | 1.1E-03 | | 1.1E-02 | | Neg | | AD | |
| Asymmetric dimethylarginine | 4.16 | 6.4E-03 | 1.5E-02 | | 1.2E-02 | | Neg | | AD | |
| β-Aminoisobutyric acid | 8.47 | 7.8E-03 | 7.0E-03 | | 1.3E-02 | | Neg | | AD | |
| Cysteine-S-sulfate | 3.63 | 8.3E-03 | 2.8E-02 | | 1.4E-02 | | Pos | | AD | |
| Cinnamoylglycine | 1.79 | 8.8E-03 | 1.5E-02 | | 1.5E-02 | | Neg | | AD | |
| 2-Furoylglycine | 7.11 | 9.2E-03 | 3.0E-03 | | 1.5E-02 | | Neg | | AD | |
| Thyroxine | 2.45 | 1.1E-02 | 7.0E-03 | | 1.8E-02 | | Pos | | AD | |
| Symmetric dimethylarginine | 3.16 | 1.2E-02 | 1.0E-01 | | 1.8E-02 | | Neg | | AD | |
| Proline | 5.84 | 1.2E-02 | 2.1E-02 | | 1.8E-02 | | Neg | | AD | |
| Alanine | 0.75 | 1.4E-02 | 1.5E-02 | | 2.0E-02 | | Pos | | AD | |
| Asparagine | 2.65 | 2.0E-02 | 1.0E-02 | | 2.7E-02 | | Pos | | AD | |
| Hypotaurine | 22.66 | 2.0E-02 | 3.8E-02 | | 2.7E-02 | | Pos | | AD | |
| Acetylhomoserine | 10.40 | 2.1E-02 | 1.5E-02 | | 2.8E-02 | | Pos | | AD | |
| N-Acetylglutamate | 1.46 | 2.2E-02 | 5.0E-02 | | 2.9E-02 | | Neg | | AD | |
| Leucine | 0.14 | 2.3E-02 | 3.3E-01 | | 2.9E-02 | | Pos | | AD | |
| Dimethylglycine | 3.73 | 2.4E-02 | 1.0E-02 | | 3.0E-02 | | Neg | | AD | |
| γ-Glutamylphenylalanine | 8.16 | 2.4E-02 | 1.0E-02 | | 3.0E-02 | | Pos | | AD | |
| Ornithine | 2.80 | 2.8E-02 | 3.8E-02 | | 3.4E-02 | | Pos | | AD | |
| Isoleucine | 0.50 | 2.9E-02 | 6.5E-02 | | 3.4E-02 | | Neg | | AD | |
| Citrulline | 10.78 | 3.1E-02 | 1.5E-02 | | 3.6E-02 | | Pos | | AD | |
| Creatinine | 3.76 | 3.7E-02 | 2.1E-02 | | 4.1E-02 | | Neg | | AD | |
| Glutamic acid | 0.66 | 3.8E-02 | 8.3E-02 | | 4.2E-02 | | Neg | | AD | |
| N-Acetyl-L-methionine | 2.45 | 4.2E-02 | 6.5E-02 | | 4.4E-02 | | Pos | | AD | |
| S-Adenosylhomocysteine | 2.34 | 4.5E-02 | 3.8E-02 | | 4.7E-02 | | Neg | | AD | |
| Hydroxyproline | 2.72 | 4.7E-02 | 1.5E-02 | | 4.8E-02 | | Neg | | AD | |
| Creatine | 0.45 | 5.0E-02 | 3.8E-02 | | 5.0E-02 | | Pos | | AD | |
| Phenylacetylglutamine | 5.08 | 5.0E-02 | 1.6E-01 | | 5.0E-02 | | Pos | | AD | |
| Pentanoate | 0.10 | 3.0E-06 | 1.6E-04 | | 1.2E-04 | | Pos | | Fatty acyls | |
| Eicosapentaenoic acid | 0.38 | 2.4E-05 | 1.6E-04 | | 4.8E-04 | | Neg | | Fatty acyls | |
| 2-Hydroxyoctarate | 4.22 | 5.8E-05 | 3.1E-04 | | 6.3E-04 | | Neg | | Fatty acyls | |
| Decanoate | 0.29 | 8.3E-05 | 1.1E-03 | | 7.7E-04 | | Neg | | Fatty acyls | |
| Decadienylcarnitine | 2.02 | 1.4E-04 | 1.6E-04 | | 1.1E-03 | | Pos | | Fatty acyls | |
| 2-Hydroxyvaleric acid | 5.07 | 1.5E-04 | 6.2E-04 | | 1.1E-03 | | Neg | | Fatty acyls | |
| Pantothenic acid | 10.26 | 3.1E-04 | 3.1E-04 | | 1.7E-03 | | Neg | | Fatty acyls | |
| 11,12,15-THETA | 4.89 | 7.0E-04 | 3.1E-04 | | 2.7E-03 | | Pos | | Fatty acyls | |
| Docosapentaenoic acid | 3.77 | 1.1E-03 | 6.2E-04 | | 3.6E-03 | | Neg | | Fatty acyls | |
| 3-methylbutanoate | 0.16 | 1.4E-03 | 1.9E-03 | | 4.3E-03 | | Pos | | Fatty acyls | |
| Octanoate | 0.26 | 1.6E-03 | 1.1E-03 | | 4.8E-03 | | Neg | | Fatty acyls | |
| Decenoylcarnitine | 4.90 | 1.7E-03 | 6.2E-04 | | 4.8E-03 | | Pos | | Fatty acyls | |
| Hexanoate | 0.19 | 1.9E-03 | 4.7E-03 | | 5.1E-03 | | Neg | | Fatty acyls | |
| 14-HDoHE | 3.35 | 2.1E-03 | 1.6E-04 | | 5.4E-03 | | Pos | | Fatty acyls | |
| Sebacate | 1.85 | 2.8E-03 | 2.1E-02 | | 6.8E-03 | | Pos | | Fatty acyls | |
| 5-Acetamidovalerate | 7.47 | 3.5E-03 | 1.6E-04 | | 8.1E-03 | | Neg | | Fatty acyls | |
| 3-Hydroxybutyrate | 0.56 | 3.6E-03 | 3.0E-03 | | 8.0E-03 | | Neg | | Fatty acyls | |
| Itaconate | 2.54 | 3.9E-03 | 3.0E-03 | | 8.4E-03 | | Pos | | Fatty acyls | |
| Palmitoleic acid | 3.06 | 4.2E-03 | 3.1E-04 | | 8.8E-03 | | Neg | | Fatty acyls | |
| 2-Isopropylmalate | 2.20 | 4.9E-03 | 3.1E-04 | | 9.5E-03 | | Pos | | Fatty acyls | |
| Azelate | 1.80 | 7.8E-03 | 2.1E-02 | | 1.3E-02 | | Neg | | Fatty acyls | |
| Palmitic acid | 0.47 | 7.8E-03 | 7.0E-03 | | 1.3E-02 | | Pos | | Fatty acyls | |
| heptanoate | 1.70 | 8.1E-03 | 2.8E-02 | | 1.4E-02 | | Neg | | Fatty acyls | |
| Pelargonic acid | 0.29 | 8.5E-03 | 4.7E-03 | | 1.4E-02 | | Neg | | Fatty acyls | |
| Ophthalmate | 3.68 | 1.0E-02 | 2.8E-02 | | 1.7E-02 | | Neg | | Fatty acyls | |
| 2-Hydroxyisobutyrate | 5.28 | 1.2E-02 | 3.8E-02 | | 1.8E-02 | | Neg | | Fatty acyls | |
| Adipate | 13.06 | 1.5E-02 | 2.1E-02 | | 2.1E-02 | | Pos | | Fatty acyls | |
| 3-Carboxy-4-methyl-5-propyl-2-furanpropanoate | 3.23 | 1.9E-02 | 7.0E-03 | | 2.6E-02 | | Pos | | Fatty acyls | |
| 2-Hydroxy-4-methylpentanoate | 6.87 | 2.0E-02 | 6.5E-02 | | 2.6E-02 | | Pos | | Fatty acyls | |
| Valerylcarnitine | 2.60 | 2.7E-02 | 2.1E-02 | | 3.3E-02 | | Pos | | Fatty acyls | |
| Methylglutarylcarnitine | 2.36 | 2.8E-02 | 5.0E-02 | | 3.4E-02 | | Pos | | Fatty acyls | |
| γ-Butyrobetaine | 1.40 | 3.0E-02 | 5.0E-02 | | 3.6E-02 | | Neg | | Fatty acyls | |
| 20-Hydroxy-leukotriene B4 | 1.82 | 3.1E-02 | 2.8E-02 | | 3.6E-02 | | Pos | | Fatty acyls | |
| Butyrate | 0.70 | 3.1E-02 | 3.8E-02 | | 3.7E-02 | | Pos | | Fatty acyls | |
| Eicosatrienoic acid | 0.63 | 3.8E-02 | 1.0E-02 | | 4.2E-02 | | Neg | | Fatty acyls | |
| Oleic acid | 0.66 | 3.9E-02 | 8.3E-02 | | 4.3E-02 | | Neg | | Fatty acyls | |
| Docosahexaenoic acid | 0.81 | 4.0E-02 | 3.8E-02 | | 4.3E-02 | | Neg | | Fatty acyls | |
| Propionic acid | 0.52 | 4.1E-02 | 1.5E-02 | | 4.4E-02 | | Neg | | Fatty acyls | |
| Citraconate | 4.21 | 4.1E-02 | 2.3E-01 | | 4.4E-02 | | Neg | | Fatty acyls | |
| Pimelic acid | 2.89 | 4.2E-02 | 1.6E-01 | | 4.5E-02 | | Neg | | Fatty acyls | |
| Acetic acid | 0.66 | 4.5E-02 | 1.3E-01 | | 4.7E-02 | | Pos | | Fatty acyls | |
| 3-Hydroxy-3-methylglutarate | 12.14 | 4.6E-02 | 2.3E-01 | | 4.7E-02 | | Pos | | Fatty acyls | |
| Glutarylcarnitine | 3.12 | 4.7E-02 | 2.8E-02 | | 4.8E-02 | | Neg | | Fatty acyls | |
| LysoPE(18:2) | 2.65 | 1.3E-06 | 1.6E-04 | | 1.3E-04 | | Neg | | GP | |
| LysoPE(20:4) | 3.34 | 9.9E-06 | 1.6E-04 | | 2.4E-04 | | Neg | | GP | |
| Glycerylphosphorylcholine | 0.42 | 2.3E-05 | 3.1E-04 | | 5.2E-04 | | Pos | | GP | |
| LysoPC(14:0) | 0.04 | 2.5E-04 | 1.6E-04 | | 1.5E-03 | | Neg | | GP | |
| LysoPC(18:0) | 2.65 | 5.3E-04 | 1.6E-04 | | 2.3E-03 | | Pos | | GP | |
| PC(36:2) | 0.65 | 6.7E-04 | 6.2E-04 | | 2.7E-03 | | Neg | | GP | |
| LysoPC(20:1) | 0.78 | 8.0E-04 | 1.1E-03 | | 3.0E-03 | | Neg | | GP | |
| LysoPC(17:0) | 3.60 | 8.7E-04 | 1.6E-04 | | 3.1E-03 | | Pos | | GP | |
| LysoPC(16:0) | 1.60 | 1.9E-03 | 4.7E-03 | | 5.3E-03 | | Neg | | GP | |
| LysoPE(16:0) | 1.82 | 2.0E-03 | 1.6E-04 | | 5.4E-03 | | Pos | | GP | |
| LysoPC(20:4) | 0.76 | 3.5E-03 | 1.0E-02 | | 8.0E-03 | | Pos | | GP | |
| LysoPE (22:1) | 1.99 | 7.5E-03 | 3.0E-03 | | 1.3E-02 | | Neg | | GP | |
| LysoPC(16:1) | 0.77 | 2.7E-02 | 2.1E-02 | | 3.3E-02 | | Pos | | GP | |
| LysoPC(18:1) | 1.78 | 2.7E-02 | 2.8E-02 | | 3.3E-02 | | Pos | | GP | |
| LysoPE(20:2) | 0.82 | 2.8E-02 | 1.0E-01 | | 3.4E-02 | | Neg | | GP | |
| LysoPE(18:0) | 0.62 | 3.1E-02 | 6.5E-02 | | 3.6E-02 | | Neg | | GP | |
| LysoPE(22:0) | 1.32 | 4.0E-02 | 1.0E-01 | | 4.4E-02 | | Neg | | GP | |
| LysoPC(18:2) | 45.73 | 4.9E-02 | 2.1E-02 | | 5.0E-02 | | Neg | | GP | |
| LysoPC(18:4) | 1.37 | 5.0E-02 | 6.5E-02 | | 5.0E-02 | | Pos | | GP | |
| Taurochenodesoxycholic acid | 3.07 | 8.1E-06 | 1.6E-04 | | 2.3E-04 | | Pos | | SL | |
| ketocholesterol | 5.54 | 8.4E-06 | 3.1E-04 | | 2.2E-04 | | Neg | | SL | |
| Glycohyodeoxycholic acid | 3.03 | 3.5E-05 | 1.6E-04 | | 5.6E-04 | | Pos | | SL | |
| Aldosterone | 2.43 | 8.4E-05 | 1.6E-04 | | 7.5E-04 | | Pos | | SL | |
| Corticosterone | 8.47 | 1.5E-03 | 1.6E-04 | | 4.5E-03 | | Pos | | SL | |
| Glycocholic acid | 1.37 | 5.7E-03 | 1.0E-02 | | 1.1E-02 | | Neg | | SL | |
| Dehydroepiandrosterone sulfate | 0.27 | 6.7E-03 | 3.0E-03 | | 1.2E-02 | | Neg | | SL | |
| Lithocholic acid | 2.64 | 1.0E-02 | 2.8E-02 | | 1.6E-02 | | Pos | | SL | |
| Taurocholic acid | 4.70 | 1.3E-02 | 2.1E-02 | | 2.0E-02 | | Pos | | SL | |
| Deoxycholic acid | 2.45 | 1.5E-02 | 2.8E-02 | | 2.1E-02 | | Neg | | SL | |
| 7-Ketolithocholic acid | 0.57 | 1.5E-02 | 1.5E-02 | | 2.1E-02 | | Neg | | SL | |
| Cholate | 3.35 | 2.8E-02 | 1.0E-02 | | 3.4E-02 | | Pos | | SL | |
| Chenodeoxycholic acid | 3.83 | 2.8E-02 | 6.5E-02 | | 3.4E-02 | | Neg | | SL | |
| 4-Androsten-3β,17β-diol disulfate | 0.68 | 3.9E-02 | 2.8E-02 | | 4.3E-02 | | Pos | | SL | |
| Tetracosahexaenoic acid | 1.41 | 4.7E-02 | 8.3E-02 | | 4.8E-02 | | Pos | | SL | |
| Equol 7-Glucoronide | 5.99 | 5.2E-03 | 1.0E-02 | | 1.0E-02 | | Neg | | Polyketides | |
| Threonate | 4.96 | 1.8E-04 | 1.9E-03 | | 1.3E-03 | | Pos | | CCC | |
| Sucrose | 1.57 | 4.9E-04 | 6.2E-04 | | 2.3E-03 | | Neg | | CCC | |
| Maltose | 9.31 | 5.2E-04 | 6.2E-04 | | 2.3E-03 | | Neg | | CCC | |
| Lactose | 5.24 | 9.4E-04 | 4.7E-03 | | 3.2E-03 | | Neg | | CCC | |
| 1,5-Anhydrosorbitol | 11.21 | 3.8E-03 | 1.5E-02 | | 8.3E-03 | | Pos | | CCC | |
| Gulose | 4.04 | 7.8E-03 | 1.9E-03 | | 1.4E-02 | | Pos | | CCC | |
| 3-Methoxy-4-hydroxyphenylglycol glucuronide | 2.74 | 1.7E-02 | 1.0E-02 | | 2.4E-02 | | Pos | | CCC | |
| Sorbitol | 66.31 | 2.7E-02 | 1.6E-04 | | 3.3E-02 | | Pos | | CCC | |
| Glucaric acid | 2.26 | 1.8E-06 | 1.6E-04 | | 9.3E-05 | | Neg | | CCC | |
| Mucate | 69.09 | 2.3E-06 | 1.6E-04 | | 1.0E-04 | | Neg | | CCC | |
| N-Acetylglucosamine | 18.10 | 3.2E-04 | 1.6E-04 | | 1.8E-03 | | Neg | | CCC | |
| Glucose 6-phosphate | 4.27 | 3.4E-04 | 1.1E-03 | | 1.8E-03 | | Neg | | CCC | |
| Gluconate | 3.91 | 4.2E-04 | 1.6E-04 | | 2.1E-03 | | Pos | | CCC | |
| Glyceraldehyde | 3.36 | 1.4E-03 | 1.6E-04 | | 4.3E-03 | | Pos | | CCC | |
| Glucuronic acid | 7.32 | 1.4E-03 | 3.1E-04 | | 4.2E-03 | | Neg | | CCC | |
| Glycerol | 4.49 | 2.6E-03 | 1.9E-03 | | 6.6E-03 | | Neg | | CCC | |
| Galacturonic acid | 4.36 | 3.8E-03 | 1.5E-02 | | 8.3E-03 | | Pos | | CCC | |
| Fructose-6-Phosphate | 2.02 | 4.7E-03 | 1.0E-02 | | 9.4E-03 | | Neg | | CCC | |
| N-Acetylneuraminic acid | 5.73 | 8.1E-03 | 1.6E-04 | | 1.4E-02 | | Neg | | CCC | |
| p-Cresol glucuronide | 2.15 | 1.2E-02 | 1.5E-02 | | 1.9E-02 | | Pos | | CCC | |
| N-Acetylmannosamine | 2.11 | 2.0E-02 | 2.1E-02 | | 2.6E-02 | | Neg | | CCC | |
| Ribulose 5-phosphate | 0.72 | 3.6E-02 | 5.0E-02 | | 4.1E-02 | | Pos | | CCC | |
| Phenol glucuronide | 10.88 | 4.1E-02 | 7.0E-03 | | 4.4E-02 | | Pos | | CCC | |
| Erythronic acid | 5.26 | 4.5E-02 | 2.8E-02 | | 4.7E-02 | | Neg | | CCC | |
| 5-Aminolevulinic acid | 0.50 | 3.4E-04 | 1.1E-03 | | 1.8E-03 | | Pos | | CAD | |
| Lactic acid | 0.60 | 6.6E-04 | 1.9E-03 | | 2.7E-03 | | Neg | | CAD | |
| Maleic acid | 2.84 | 1.2E-03 | 7.0E-03 | | 3.8E-03 | | Neg | | CAD | |
| Malate | 16.82 | 1.2E-03 | 1.6E-04 | | 3.9E-03 | | Pos | | CAD | |
| Nicotinuric acid | 5.73 | 1.5E-03 | 3.0E-03 | | 4.4E-03 | | Pos | | CAD | |
| Succinic acid | 7.54 | 1.5E-03 | 3.1E-04 | | 4.4E-03 | | Neg | | CAD | |
| 4-Acetamidobutanoic acid | 0.10 | 1.8E-03 | 1.1E-03 | | 5.1E-03 | | Neg | | CAD | |
| Malonic acid | 2.88 | 4.8E-03 | 4.7E-03 | | 9.5E-03 | | Neg | | CAD | |
| Isocitric acid | 1.78 | 6.2E-03 | 1.5E-02 | | 1.1E-02 | | Neg | | CAD | |
| Aconitic acid | 17.63 | 8.9E-03 | 2.1E-02 | | 1.5E-02 | | Neg | | CAD | |
| Methylmalonic acid | 59.56 | 1.4E-02 | 4.7E-03 | | 2.0E-02 | | Neg | | CAD | |
| Citramalate | 1.29 | 2.7E-02 | 2.1E-02 | | 3.3E-02 | | Neg | | CAD | |
| Citrate | 2.10 | 4.6E-02 | 2.8E-02 | | 4.7E-02 | | Pos | | CAD | |
| Glutaric acid | 2.67 | 4.7E-02 | 6.5E-02 | | 4.8E-02 | | Neg | | CAD | |
| Fumaric acid | 1.83 | 4.9E-02 | 1.3E-01 | | 5.0E-02 | | Neg | | CAD | |
| Isethionate | 4.39 | 7.1E-06 | 1.6E-04 | | 2.2E-04 | | Neg | | OSD | |
| p-Cresol sulfate | 5.63 | 3.5E-05 | 1.6E-04 | | 5.7E-04 | | Neg | | OSD | |
| 4-Phenylethyl Sulfate | 40.61 | 3.8E-05 | 1.6E-04 | | 5.4E-04 | | Neg | | OSD | |
| 2-Aminophenol sulfate | 3.65 | 2.5E-04 | 1.6E-04 | | 1.5E-03 | | Neg | | OSD | |
| Tyramine-O-sulfate | 4.68 | 5.1E-04 | 1.6E-04 | | 2.3E-03 | | Neg | | OSD | |
| Pyrocatechol Sulfate | 7.37 | 1.1E-03 | 1.0E-02 | | 3.6E-03 | | Neg | | OSD | |
| Glycerolphosphate | 6.70 | 1.8E-03 | 3.0E-03 | | 5.1E-03 | | Neg | | OSD | |
| Taurocyamine | 4.32 | 2.8E-03 | 1.6E-04 | | 6.8E-03 | | Pos | | OSD | |
| Phenol sulfate | 4.41 | 6.7E-03 | 1.1E-03 | | 1.2E-02 | | Pos | | OSD | |
| MOPEG | 2.11 | 4.1E-02 | 6.5E-02 | | 4.4E-02 | | Neg | | OSD | |
| 7-Methylguanine | 4.70 | 1.3E-03 | 1.9E-03 | | 4.0E-03 | | Neg | | PD | |
| Adenine | 1.84 | 2.6E-03 | 1.9E-03 | | 6.5E-03 | | Pos | | PD | |
| Hypoxanthine | 0.52 | 3.1E-03 | 7.0E-03 | | 7.5E-03 | | Neg | | PD | |
| Dimethyluric acid | 11.99 | 3.2E-03 | 6.2E-04 | | 7.6E-03 | | Pos | | PD | |
| Uric acid | 9.92 | 3.5E-03 | 3.0E-03 | | 8.0E-03 | | Pos | | PD | |
| Kinetin | 0.50 | 4.7E-03 | 4.7E-03 | | 9.5E-03 | | Pos | | PD | |
| 1-Methyladenosine | 1.63 | 1.3E-02 | 2.1E-02 | | 2.0E-02 | | Pos | | PD | |
| 2,8-Dihydroxyadenine | 2.31 | 2.3E-02 | 3.8E-02 | | 3.0E-02 | | Pos | | PD | |
| Xanthine | 0.61 | 3.7E-02 | 2.8E-02 | | 4.1E-02 | | Neg | | PD | |
| Methyluric acid | 3.41 | 3.8E-02 | 2.1E-02 | | 4.2E-02 | | Neg | | PD | |
| 2-Oxoisopentanoate | 0.48 | 4.9E-05 | 1.6E-04 | | 6.0E-04 | | Neg | | KAD | |
| 4-Methyl-2-oxopentanoate | 0.24 | 5.9E-04 | 1.1E-03 | | 2.5E-03 | | Pos | | KAD | |
| 3-Methyl-2-oxovaleric acid | 6.32 | 2.0E-03 | 1.9E-03 | | 5.4E-03 | | Neg | | KAD | |
| 2-Oxoadipate | 3.21 | 5.7E-03 | 1.9E-03 | | 1.1E-02 | | Pos | | KAD | |
| Pyruvic acid | 7.82 | 6.4E-03 | 1.6E-04 | | 1.2E-02 | | Neg | | KAD | |
| 4-Oxopentanoate | 2.56 | 1.1E-02 | 4.7E-03 | | 1.8E-02 | | Neg | | KAD | |
| 2-Oxoglutarate | 54.41 | 2.0E-02 | 6.2E-04 | | 2.7E-02 | | Pos | | KAD | |
| acetoacetate | 1.48 | 3.1E-02 | 1.5E-02 | | 3.6E-02 | | Neg | | KAD | |
| Xanthosine | 2.18 | 2.4E-05 | 1.6E-04 | | 5.0E-04 | | Neg | | PN | |
| N2,N2-Dimethylguanosine | 4.00 | 3.3E-04 | 3.1E-04 | | 1.8E-03 | | Pos | | PN | |
| N6-Succinyl Adenosine | 3.95 | 7.9E-04 | 6.2E-04 | | 3.0E-03 | | Pos | | PN | |
| Inosine | 0.23 | 1.2E-03 | 3.0E-03 | | 3.9E-03 | | Neg | | PN | |
| Methylinosine | 1.87 | 2.1E-03 | 4.7E-03 | | 5.5E-03 | | Neg | | PN | |
| Guanosine | 5.52 | 3.4E-03 | 1.1E-03 | | 7.9E-03 | | Neg | | PN | |
| 2-Deoxyadenosine | 4.04 | 1.1E-02 | 7.0E-03 | | 1.7E-02 | | Neg | | PN | |
| Adenosine | 3.33 | 1.8E-02 | 3.8E-02 | | 2.4E-02 | | Pos | | PN | |
| Phytosphingosine | 0.03 | 1.5E-08 | 1.6E-04 | | 2.3E-06 | | Neg | | Amines | |
| 1-Aminopyrene | 11.67 | 5.1E-06 | 1.6E-04 | | 1.8E-04 | | Pos | | Amines | |
| Trimethylamine N-oxide | 3.69 | 8.3E-04 | 1.6E-04 | | 3.0E-03 | | Pos | | Amines | |
| Dimethylamine | 7.92 | 3.5E-03 | 1.6E-04 | | 8.0E-03 | | Pos | | Amines | |
| Aspidospermine | 4.27 | 2.3E-02 | 8.3E-02 | | 2.9E-02 | | Pos | | Amines | |
| Sphinganine | 0.22 | 3.7E-02 | 3.0E-03 | | 4.2E-02 | | Neg | | Amines | |
| Sphingosine | 5.69 | 4.1E-02 | 6.5E-02 | | 4.4E-02 | | Neg | | Amines | |
| Spermidine | 1.36 | 4.6E-02 | 8.3E-02 | | 4.7E-02 | | Pos | | Amines | |
| Methylguanidine | 13.40 | 1.5E-04 | 1.6E-04 | | 1.1E-03 | | Neg | | Guanidines | |
| Guanidinopropionic acid | 2.96 | 8.9E-04 | 3.1E-04 | | 3.1E-03 | | Pos | | Guanidines | |
| Guanidinosuccinic acid | 2.28 | 2.7E-03 | 1.1E-03 | | 6.7E-03 | | Pos | | Guanidines | |
| Guanidinobutanoic acid | 3.45 | 4.4E-03 | 1.6E-04 | | 9.0E-03 | | Pos | | Guanidines | |
| Guanidine | 7.28 | 7.4E-03 | 4.7E-03 | | 1.3E-02 | | Pos | | Guanidines | |
| Guanidoacetic acid | 1.57 | 3.4E-02 | 3.8E-02 | | 3.9E-02 | | Pos | | Guanidines | |
| 2-Deoxyuridine | 3.15 | 9.3E-04 | 1.0E-02 | | 3.2E-03 | | Neg | | PMN | |
| Uridine | 4.19 | 1.4E-03 | 1.9E-03 | | 4.3E-03 | | Pos | | PMN | |
| Cytidine | 9.33 | 5.2E-03 | 3.1E-04 | | 1.0E-02 | | Pos | | PMN | |
| Thymidine | 3.83 | 1.3E-02 | 3.0E-03 | | 2.0E-02 | | Pos | | PMN | |
| 3-Methyluridine | 9.33 | 2.0E-02 | 1.9E-03 | | 2.7E-02 | | Pos | | PMN | |
| Methyl pyridone carboxamide | 25.75 | 7.4E-04 | 1.6E-04 | | 2.9E-03 | | Neg | | PDD | |
| Nicotinamide | 0.40 | 7.4E-03 | 1.1E-03 | | 1.3E-02 | | Pos | | PDD | |
| 1-Methylnicotinamide | 0.55 | 1.2E-02 | 2.8E-02 | | 1.8E-02 | | Pos | | PDD | |
| Orotate | 9.71 | 2.5E-02 | 1.1E-03 | | 3.1E-02 | | Pos | | PMD | |
| Cytosine | 2.04 | 4.2E-02 | 2.1E-02 | | 4.5E-02 | | Neg | | PMD | |
| Urea | 4.21 | 6.2E-03 | 6.2E-04 | | 1.1E-02 | | Pos | | Ureas | |
| Ureidopropionic acid | 3.99 | 1.4E-02 | 1.9E-03 | | 2.0E-02 | | Pos | | Ureas | |
| Allantoin | 3.62 | 6.4E-05 | 1.6E-04 | | 6.2E-04 | | Neg | | Imidazoles | |
| Urocanic acid | 6.66 | 3.6E-02 | 2.8E-02 | | 4.1E-02 | | Neg | | Imidazoles | |
| Carnitine | 0.43 | 1.5E-06 | 1.6E-04 | | 1.1E-04 | | Pos | | Others | |
| 4-Hydroxybenzenesulfonic acid | 9.58 | 1.6E-06 | 1.6E-04 | | 9.8E-05 | | Neg | | Others | |
| Phthalic acid | 5.74 | 5.9E-05 | 3.1E-04 | | 6.1E-04 | | Neg | | Others | |
| 3-Phosphoglyceric acid | 0.21 | 6.8E-05 | 1.1E-03 | | 6.5E-04 | | Pos | | Others | |
| 4-Hydroxy-3-methoxybenzoate | 10.52 | 1.2E-04 | 6.2E-04 | | 1.0E-03 | | Neg | | Others | |
| Homogentisic acid | 7.07 | 1.8E-04 | 7.0E-03 | | 1.3E-03 | | Neg | | Others | |
| Quinate | 11.78 | 2.4E-04 | 1.6E-04 | | 1.5E-03 | | Pos | | Others | |
| Choline | 1.76 | 2.7E-04 | 1.1E-03 | | 1.6E-03 | | Pos | | Others | |
| Nicotinamide adenine dinucleotide | 15.33 | 3.5E-04 | 1.6E-04 | | 1.8E-03 | | Neg | | Others | |
| 2-Hydroxypyridine | 10.40 | 4.6E-04 | 1.1E-03 | | 2.2E-03 | | Neg | | Others | |
| 4-Pyridoxic acid | 7.48 | 4.6E-04 | 1.6E-04 | | 2.2E-03 | | Neg | | Others | |
| Cinnabarinic acid | 2.96 | 4.9E-04 | 6.2E-04 | | 2.3E-03 | | Pos | | Others | |
| 5'-Phosphoribosyl-N-formylglycinamide | 0.42 | 5.0E-04 | 4.7E-03 | | 2.3E-03 | | Pos | | Others | |
| Pseudouridine | 2.40 | 5.4E-04 | 1.6E-04 | | 2.3E-03 | | Neg | | Others | |
| 5-Methoxysalicylic acid | 3.28 | 5.7E-04 | 3.0E-03 | | 2.4E-03 | | Neg | | Others | |
| Phenylpyruvic acid | 9.08 | 1.9E-03 | 3.0E-03 | | 5.3E-03 | | Neg | | Others | |
| N-Acetylcarnosine | 4.27 | 3.7E-03 | 6.2E-04 | | 8.3E-03 | | Pos | | Others | |
| 3-(4-Hydroxyphenyl)propionate | 10.60 | 4.3E-03 | 4.7E-03 | | 8.9E-03 | | Neg | | Others | |
| Phenylacetic acid | 112.48 | 8.1E-03 | 3.1E-04 | | 1.4E-02 | | Neg | | Others | |
| Trigonelline | 2.12 | 9.3E-03 | 1.0E-02 | | 1.5E-02 | | Pos | | Others | |
| Homovanillic acid | 2.84 | 9.5E-03 | 4.7E-03 | | 1.5E-02 | | Pos | | Others | |
| Myoinositol | 1.99 | 1.4E-02 | 2.1E-02 | | 2.0E-02 | | Pos | | Others | |
| Methyltetrahydrofolate | 4.95 | 1.6E-02 | 2.1E-02 | | 2.2E-02 | | Neg | | Others | |
| 3-Mercaptolactic acid | 22.59 | 1.7E-02 | 2.1E-02 | | 2.4E-02 | | Pos | | Others | |
| Dimethyl sulfone | 11.60 | 1.9E-02 | 3.0E-03 | | 2.6E-02 | | Pos | | Others | |
| Carnosine | 2.16 | 2.2E-02 | 7.0E-03 | | 2.9E-02 | | Neg | | Others | |
| Phenyllactic acid | 0.72 | 2.9E-02 | 6.5E-02 | | 3.5E-02 | | Pos | | Others | |
| 4-Hydroxy-3-methoxymandelate | 4.94 | 3.4E-02 | 2.1E-02 | | 3.9E-02 | | Pos | | Others | |
| Cyclic GMP | 7.04 | 4.4E-02 | 7.0E-03 | | 4.6E-02 | | Neg | | Others | |
| 4-Hydroxybenzoate | 65.92 | 4.6E-02 | 6.5E-02 | | 4.8E-02 | | Neg | | Others | |
| 5-Methylcytosine | 3.78 | 4.6E-02 | 1.6E-01 | | 4.8E-02 | | Pos | | Others | |

^a^FC was obtained by comparing those metabolites in CRF rats with control rats; FC with a value > 1 indicated a relatively higher intensity present in CRF rats, whereas a value < 1 indicated a relatively lower intensity compared with control rats. ^b^*P*-values from one-way ANOVA; ^c^*P*-values from Mann-Whitney U-test. ^d^FDR value was obtained from the adjusted *P* value using the Benjamini Hochberg method. AAD, amino acid and derivatives; CAD, carboxylic acids and derivatives; CCC, carbohydrates and carbohydrate conjugates; GP, glycerophospholipids; KAD, keto acids and derivatives; OSD, organic sulfonic acids and derivatives; PD, purines and derivatives; PDD, pyridines and derivatives; PMD, pyrimidines and derivatives; PMN, pyrimidine nucleosides; PN, purine nucleosides; SL, sterol lipids; TD, tryptophan and derivatives.

**Table S4.**

Top seven metabolic pathways based on low p values or with high impact

| Pathway Name | Total | Hits | p | -log(p) | Holm p | FDR | Impact |
| --- | --- | --- | --- | --- | --- | --- | --- |
| Arginine biosynthesis | 14 | 10 | 2.6E-06 | 5.6E+00 | 2.2E-04 | 2.2E-04 | 4.8E-01 |
| Tryptophan metabolism | 14 | 14 | 1.5E-03 | 2.8E+00 | 1.2E-01 | 2.5E-02 | 6.5E-01 |
| Alanine, aspartate and glutamate metabolism | 28 | 11 | 1.3E-03 | 2.9E+00 | 1.1E-01 | 2.5E-02 | 6.7E-01 |
| Phenylalanine metabolism | 12 | 6 | 4.3E-03 | 2.4E+00 | 3.4E-01 | 6.1E-02 | 6.2E-01 |
| [Phenylalanine, tyrosine and tryptophan biosynthesis](https://dev.metaboanalyst.ca/MetaboAnalyst/Secure/pathway/ResultView.xhtml) | 4 | 3 | 1.2E-02 | 1.9E+00 | 9.0E-01 | 1.2E-01 | 1.0E+00 |
| [Taurine and hypotaurine metabolism](https://dev.metaboanalyst.ca/MetaboAnalyst/Secure/pathway/ResultView.xhtml) | 8 | 4 | 2.1E-02 | 1.7E+00 | 1.0E+00 | 1.6E-01 | 7.1E-01 |
| [Synthesis and degradation of ketone bodies](https://dev.metaboanalyst.ca/MetaboAnalyst/Secure/pathway/ResultView.xhtml) | [5](https://dev.metaboanalyst.ca/MetaboAnalyst/Secure/pathway/ResultView.xhtml) | 2 | 1.6E-01 | 7.9E-01 | 1.0E+00 | 7.2E-01 | 6.0E-01 |

**Table S5.**

Analysis of PLS-DA based ROC curves of 8 metabolites in healthy control and CRF rats

| Metabolites | AUC | 95% CI | Sensitivity | Specificity |
| --- | --- | --- | --- | --- |
| Indole-3-aldehyde | 0.938 | 0.721 - 0.998 | 87.5 | 87.5 |
| Kynurenic acid | 0.984 | 0.768 - 1.000 | 100 | 87.5 |
| Indoxyl sulfate | 1.000 | 0.794 - 1.000 | 100 | 100 |
| 5-Methoxytryptophan | 0.953 | 0.720 - 1.000 | 100 | 87.5 |
| 3-Methylindole | 0.953 | 0.720 - 1.000 | 100 | 87.5 |
| N-Acetyltryptophan | 1.000 | 0.794 - 1.000 | 100 | 100 |
| Indole-3-acetamide | 0.891 | 0.636 - 0.989 | 87.5 | 87.5 |
| 6-Sulfatoxymelatonin | 0.969 | 0.743 - 1.000 | 100 | 87.5 |

**Table S6.**

Clinical and demographic baseline characteristics of health controls and patients with CKD

| Variable | Healthy controls | CKD |
| --- | --- | --- |
| Sample size | 80 | 120 |
| Men (%) | 52.3 | 48.3 |
| Age (years) | 54.9±1.4 | 57.8±1.4 |
| Body weight (kg) | 66.4±1.3 | 77.4±1.0** |
| Body mass index (kg/m2) | 24.5±0.38 | 25.7±0.3* |
| SBP (mmHg) | 121.6±2.0 | 140.1±2.1** |
| DBP (mmHg) | 75.4±1.3 | 80.0±1.4* |
| eGFR (ml/min/1.73m^2^) | 99.9±1.3 | 14.2±0.7** |
| Total protein (g/L) | 72.8±0.3 | 61.1±0.8** |
| Albumin (g/L) | 47.3±0.5 | 35.1±0.6** |
| TC (mmol/L) | 4.55±0.14 | 4.50±0.13 |
| Triglyceride (mmol/L) | 1.82±0.18 | 1.60±0.12 |
| HDL-C (mmol/L) | 1.51±0.08 | 1.55±0.04 |
| LDL-C (mmol/L) | 2.93±0.11 | 3.55±0.15** |
| Creatinine (μmol/L) | 68.4±1.5 | 481.8±29.9** |
| Urea (mmol/L) | 5.19±0.11 | 18.9±0.7** |
| Uric acid (μmol/L) | 328.9±10.4 | 420.4±12.5** |
| Urine P/C ratio | 0.149±0.005 | 2.52±0.21** |
| Proteinuria (g/24h) | 0.064±0.002 | 2.03±0.18** |

Results are expressed as the mean ± SE. **P*<0.05, ***P*<0.01 compared with healthy controls.

**Table S7.**

Catalog pathway of gut microbiota in the fecal samples of control and adenine-induced CRF rats

| Catalog pathway | CTL | CRF | P | P^a^ |
| --- | --- | --- | --- | --- |
| Biosynthesis of other secondary metabolites | 318666 | 293962 | 1.20E-04 | 1.32E-03 |
| Glycan biosynthesis and metabolism | 693742 | 656063 | 6.53E-03 | 3.59E-02 |
| Xenobiotics biodegradation and metabolism | 156542 | 166060 | 3.42E-02 | 1.25E-01 |
| Nucleotide metabolism | 680193 | 705403 | 6.20E-02 | 1.42E-01 |
| Metabolism of cofactors and vitamins | 830066 | 810096 | 6.45E-02 | 1.42E-01 |
| Carbohydrate metabolism | 1771811 | 1735577 | 1.90E-01 | 3.48E-01 |
| Metabolism of other amino acids | 372648 | 365254 | 2.69E-01 | 4.23E-01 |
| Lipid metabolism | 430309 | 420981 | 3.45E-01 | 4.74E-01 |
| Amino acid metabolism | 1227032 | 1238280 | 3.92E-01 | 4.79E-01 |
| Metabolism of terpenoids and polyketides | 192963 | 194484 | 6.64E-01 | 7.30E-01 |
| Energy metabolism | 801588 | 801679 | 9.94E-01 | 9.94E-01 |
