# Supplementary figures and images for: Targeting Lactobacillus johnsonii to reverse chronic kidney disease
Source: Signal Transduct Target Ther. 2024 Aug 5;9:195. doi: 10.1038/s41392-024-01913-1 (PMC11298530; doi:10.1038/s41392-024-01913-1)

Figure 4d

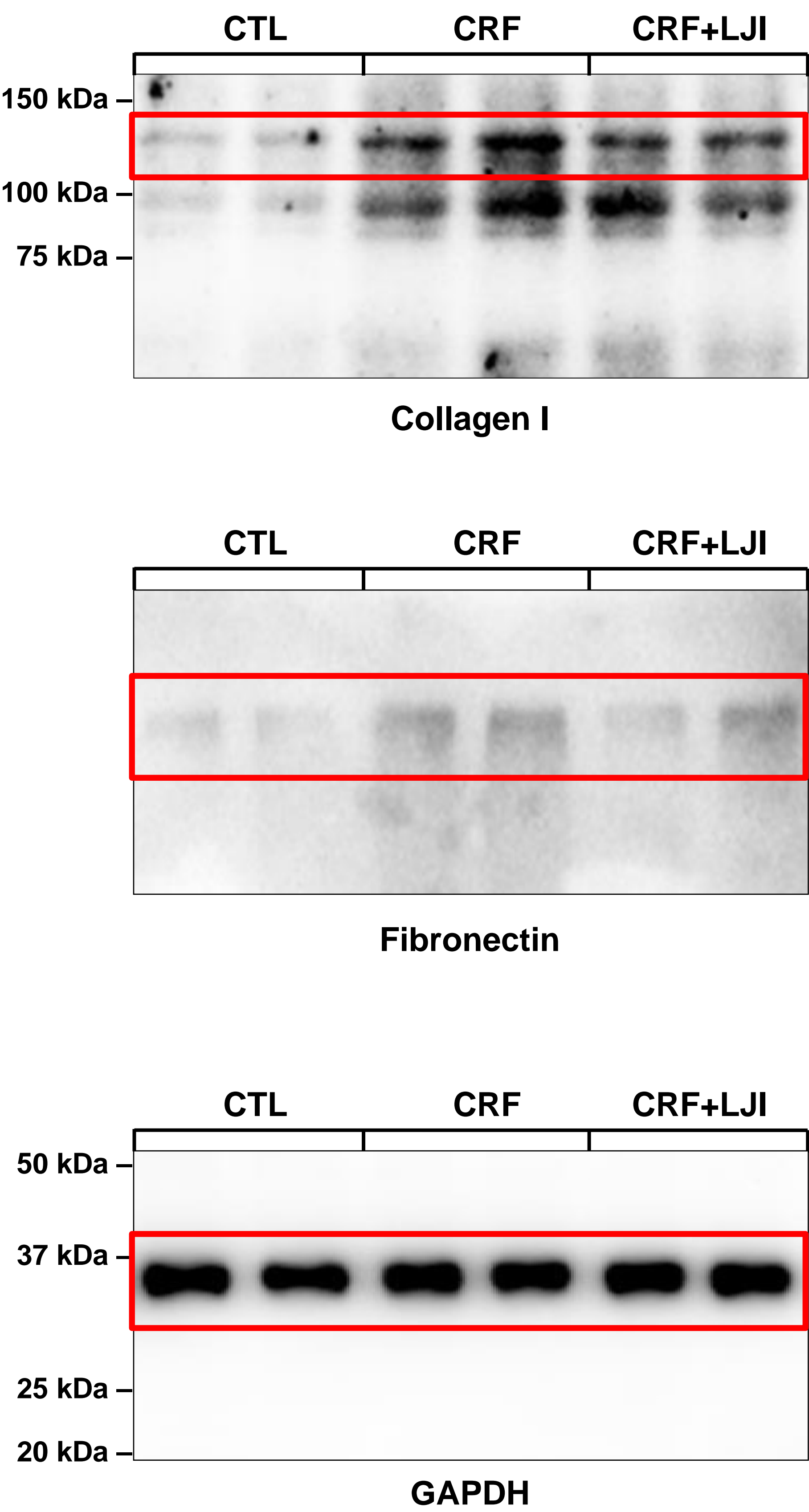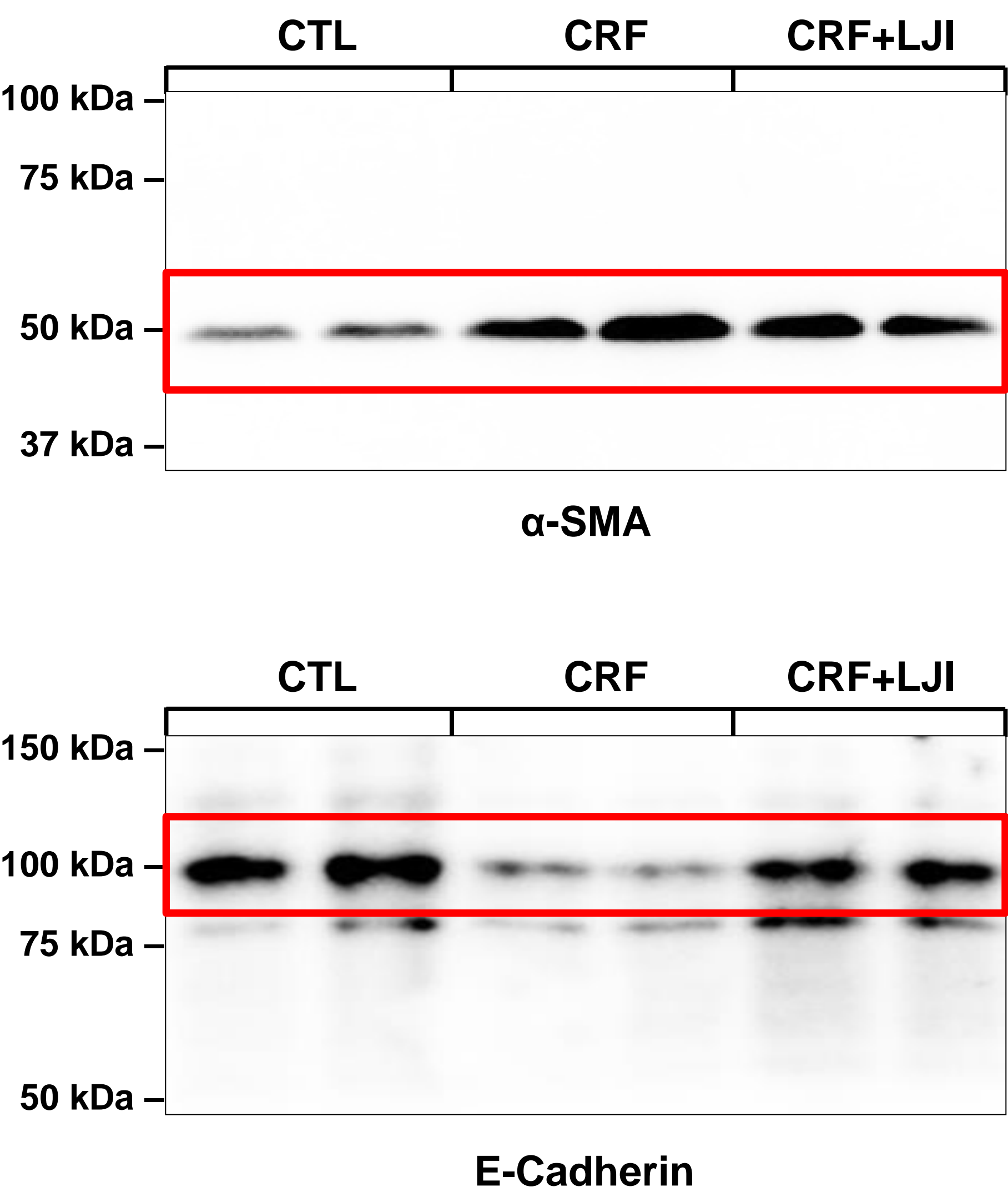

Figure 4f

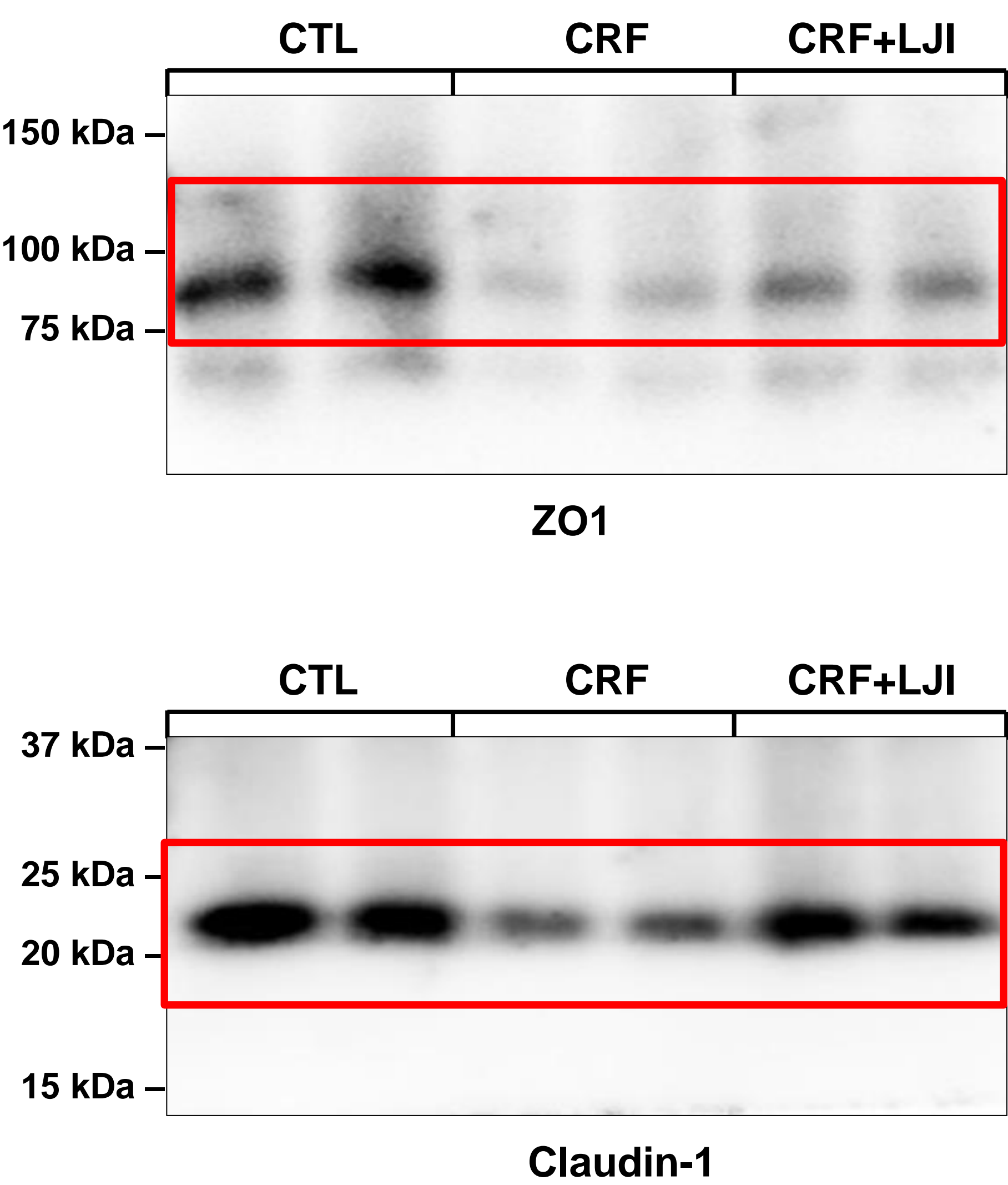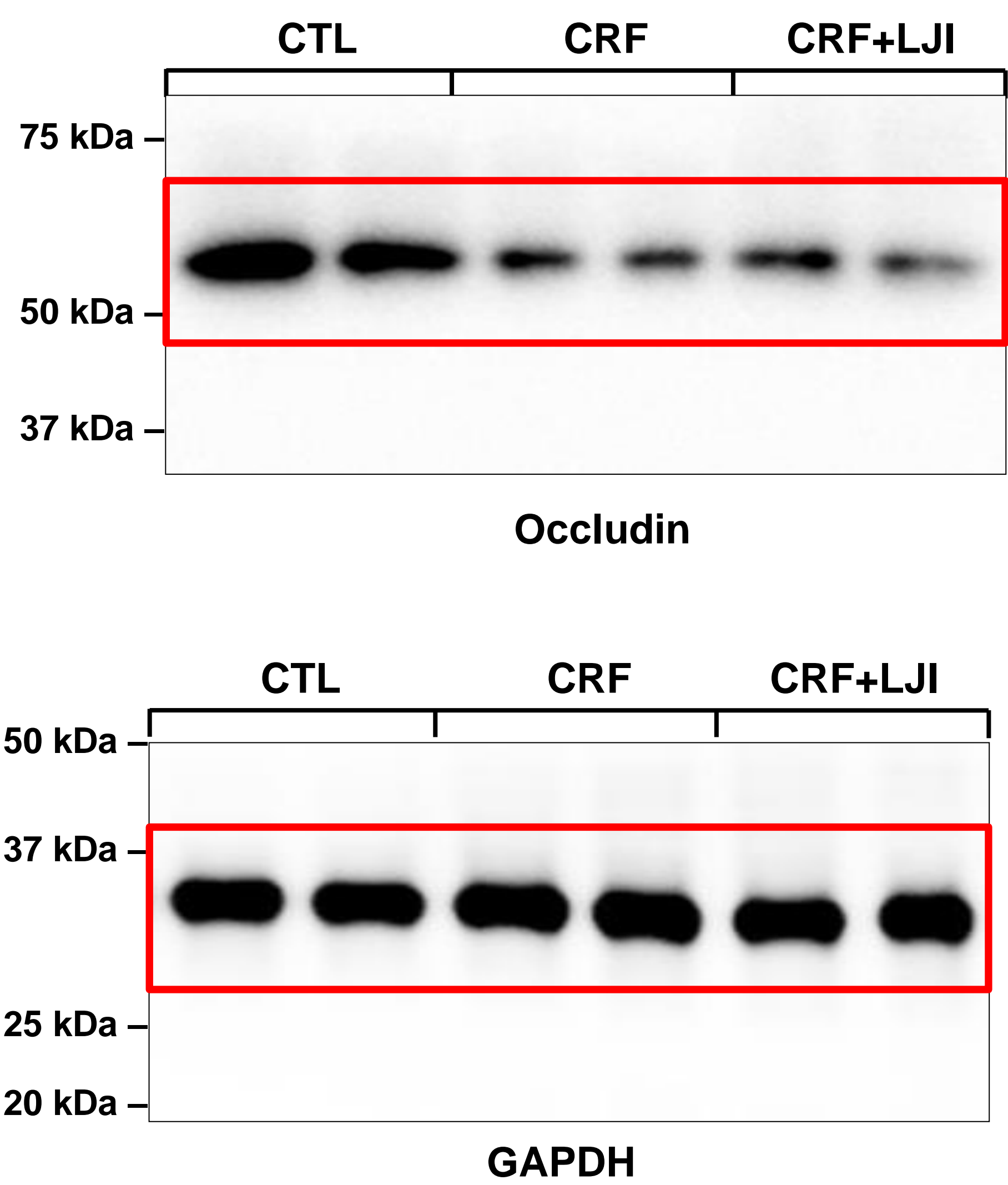

Figure 8e

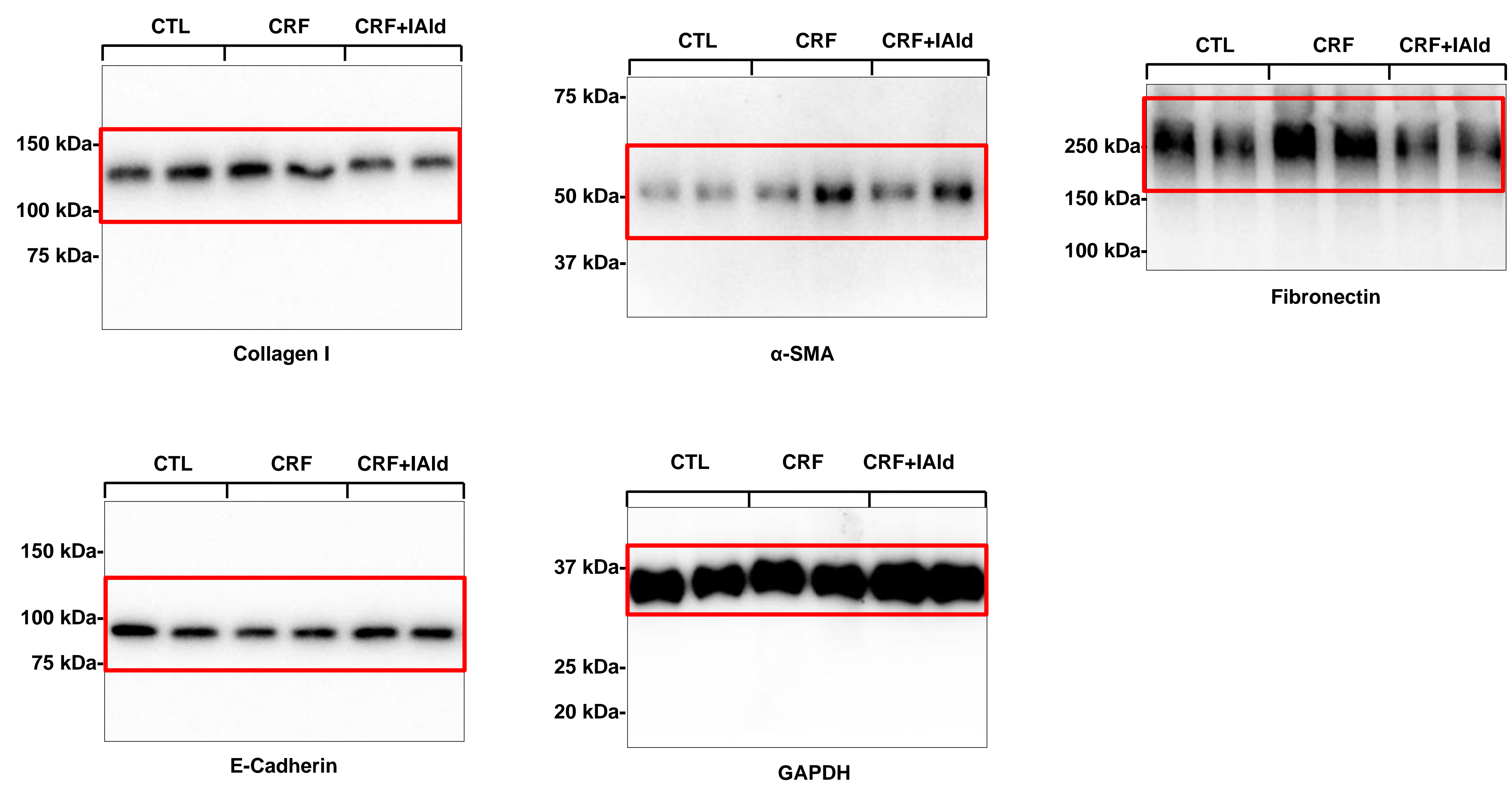

Figure 8g

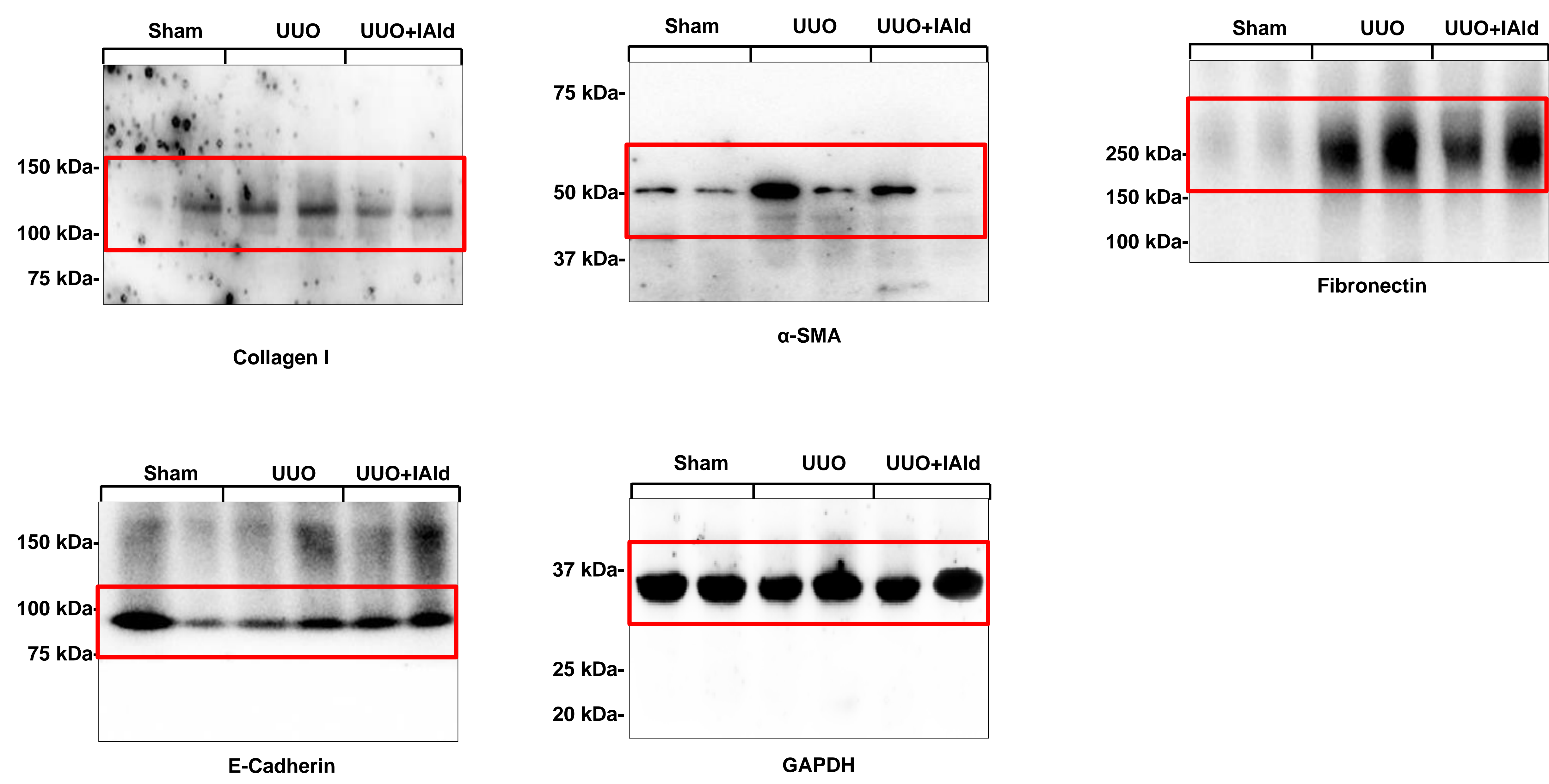

Figure 8k

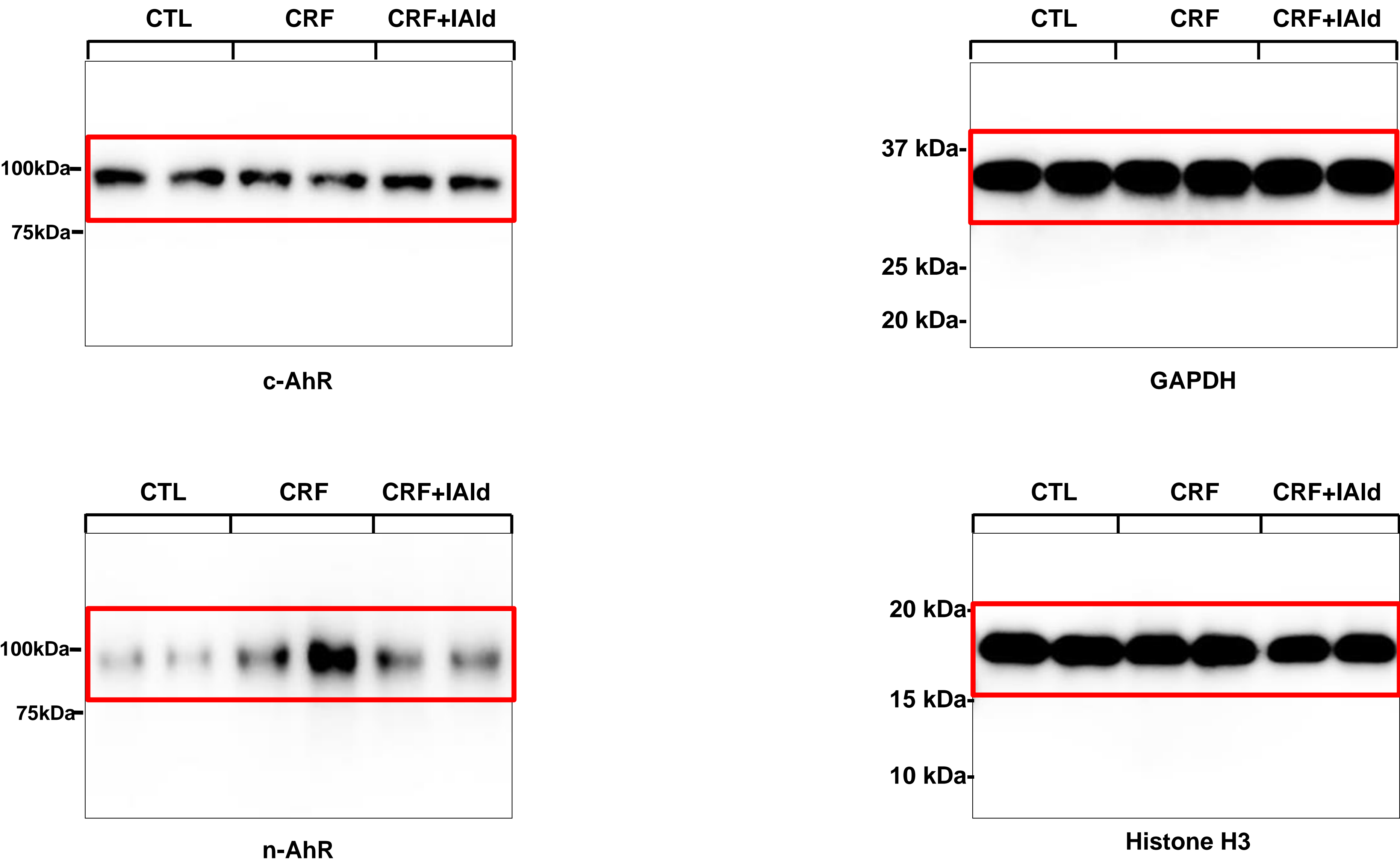

Figure 8n

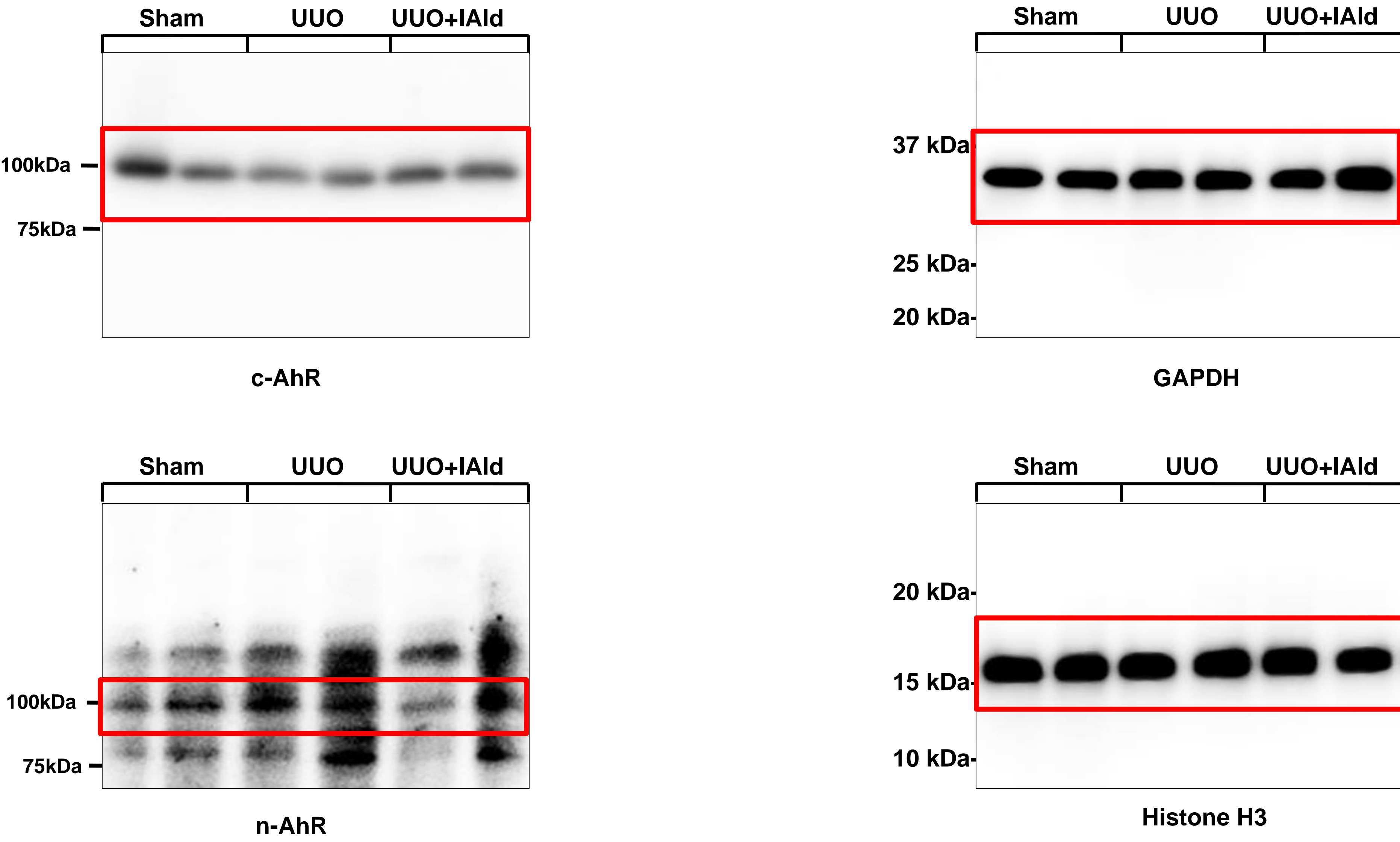

Figure 9d

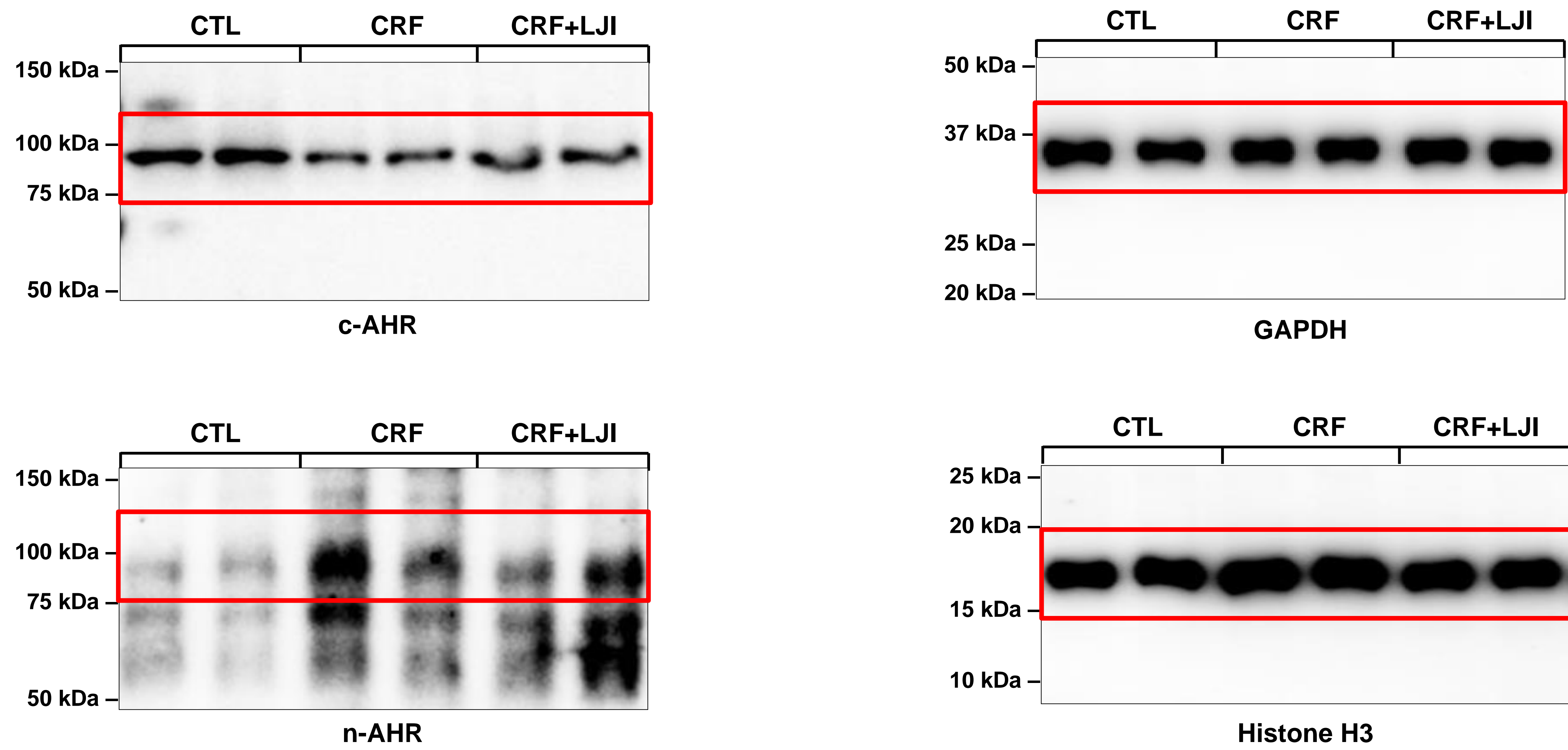

Figure 9h

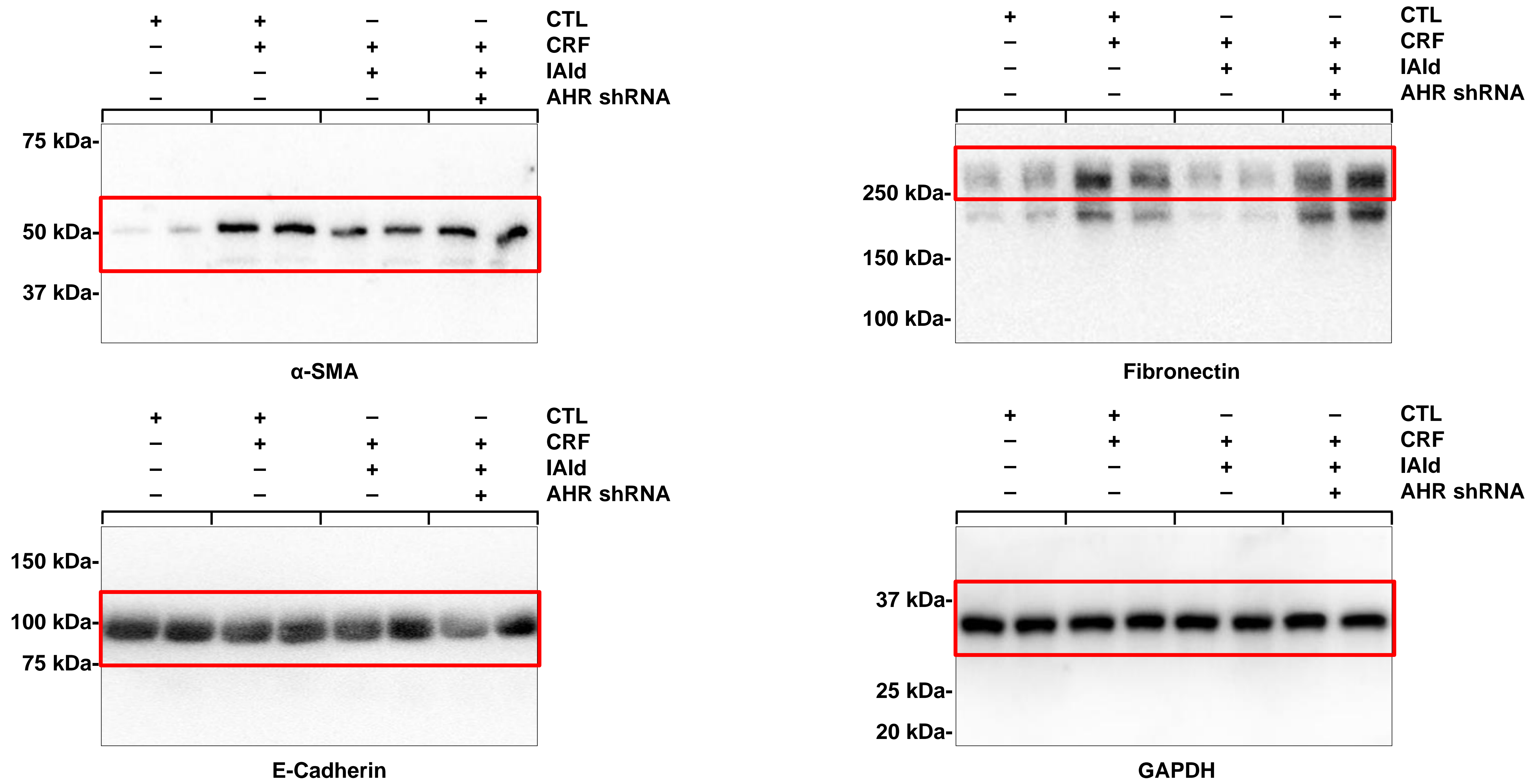

Figure 9k

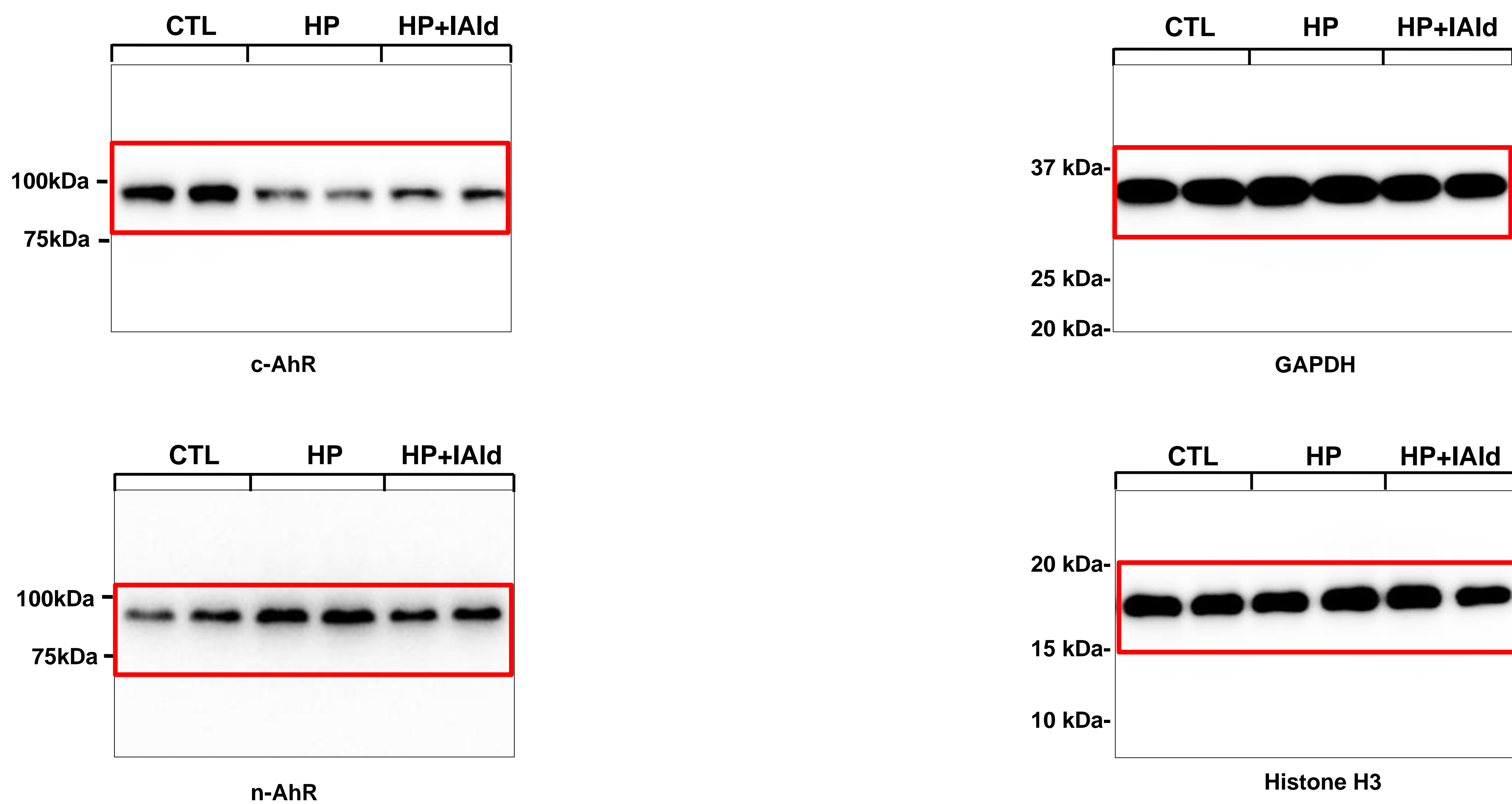

Figure 9n

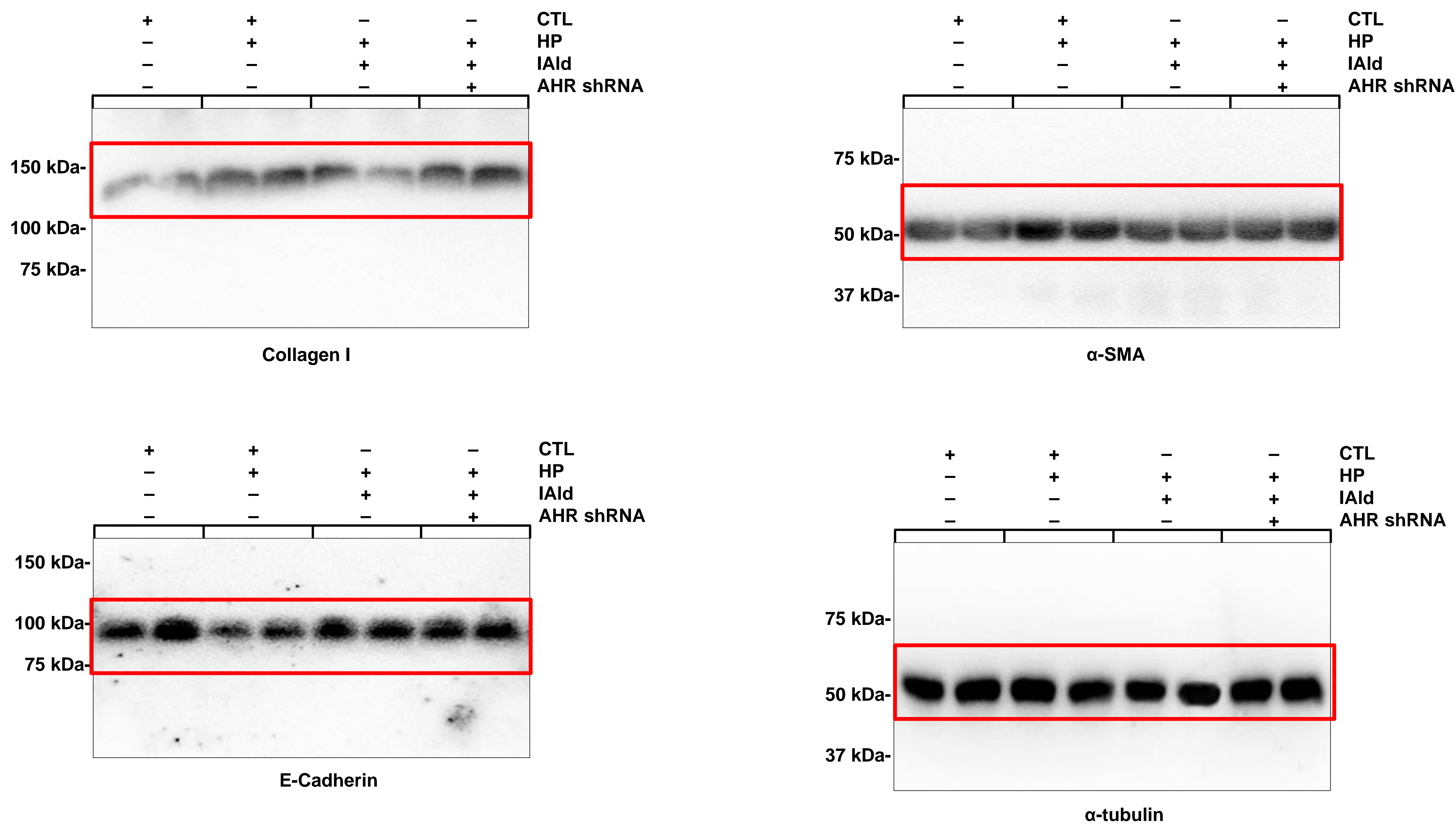

Figure S14b

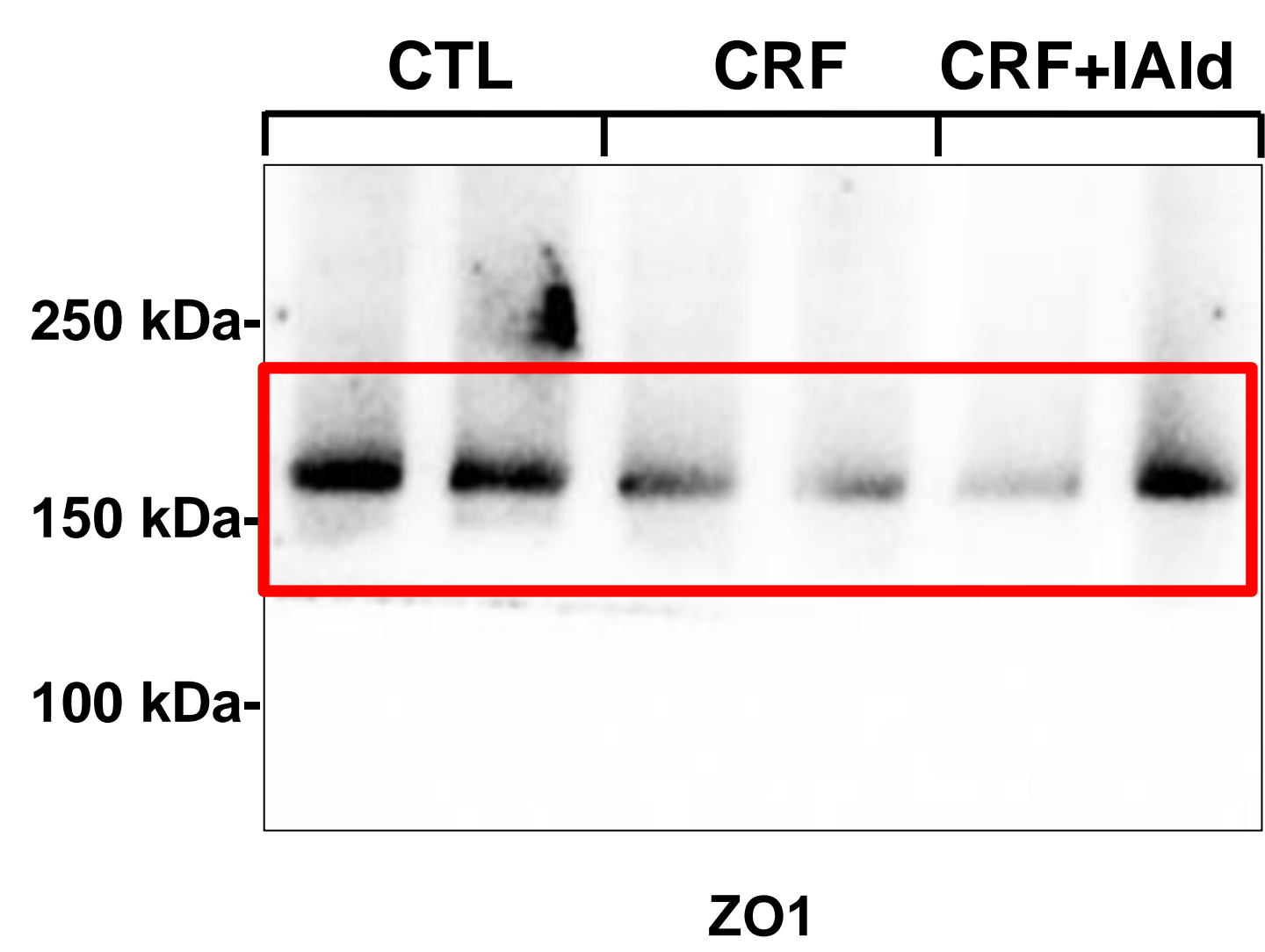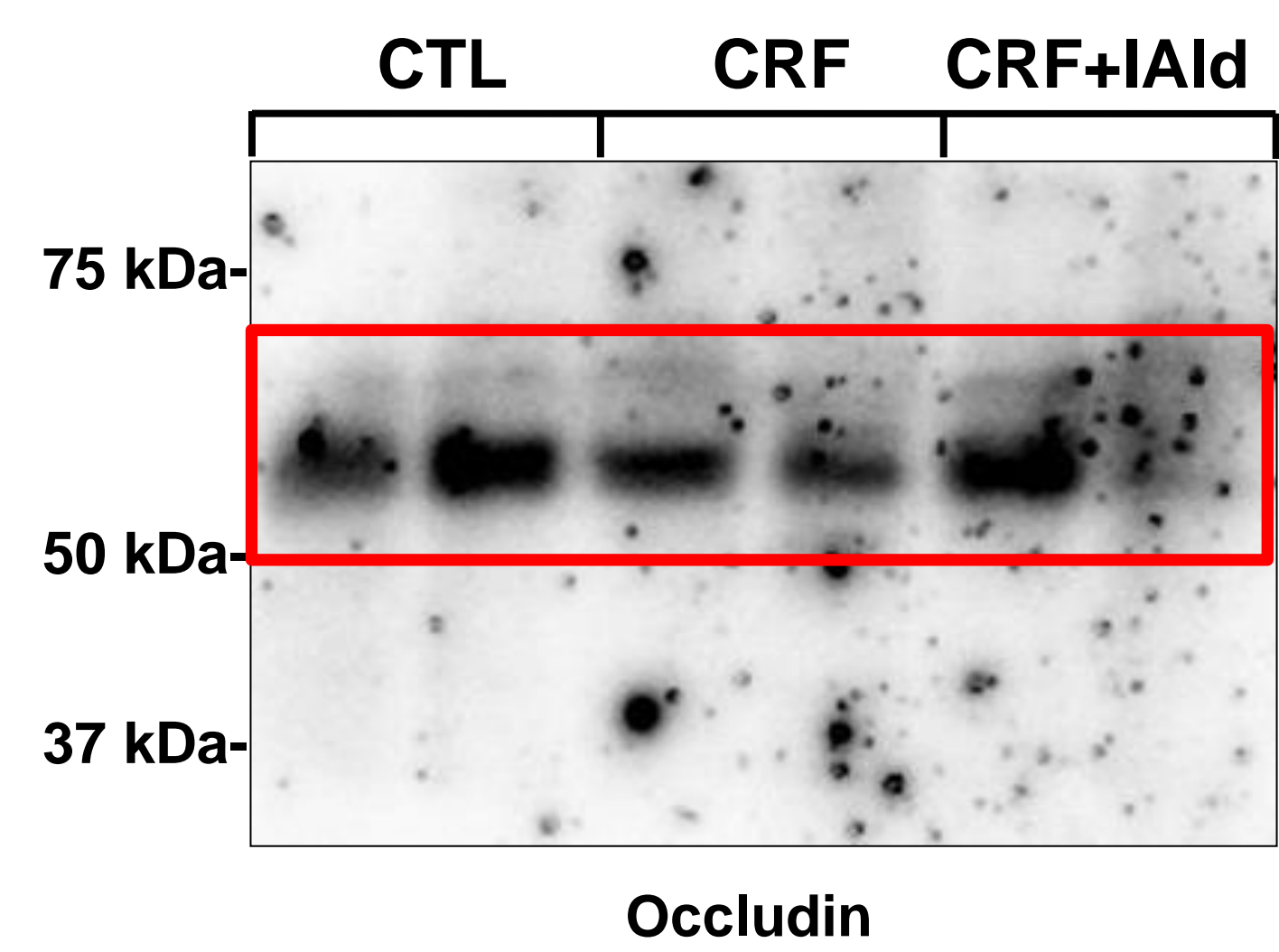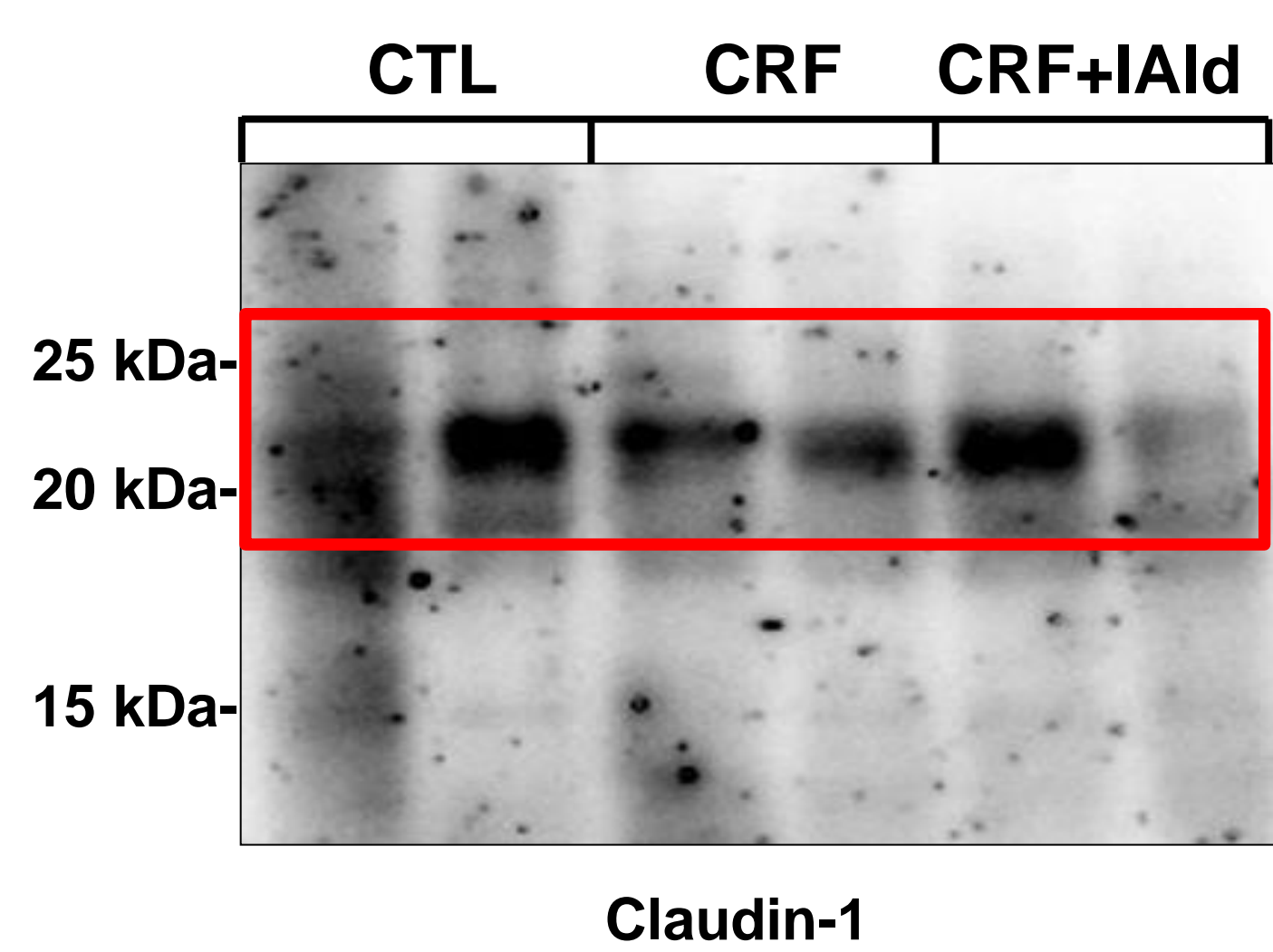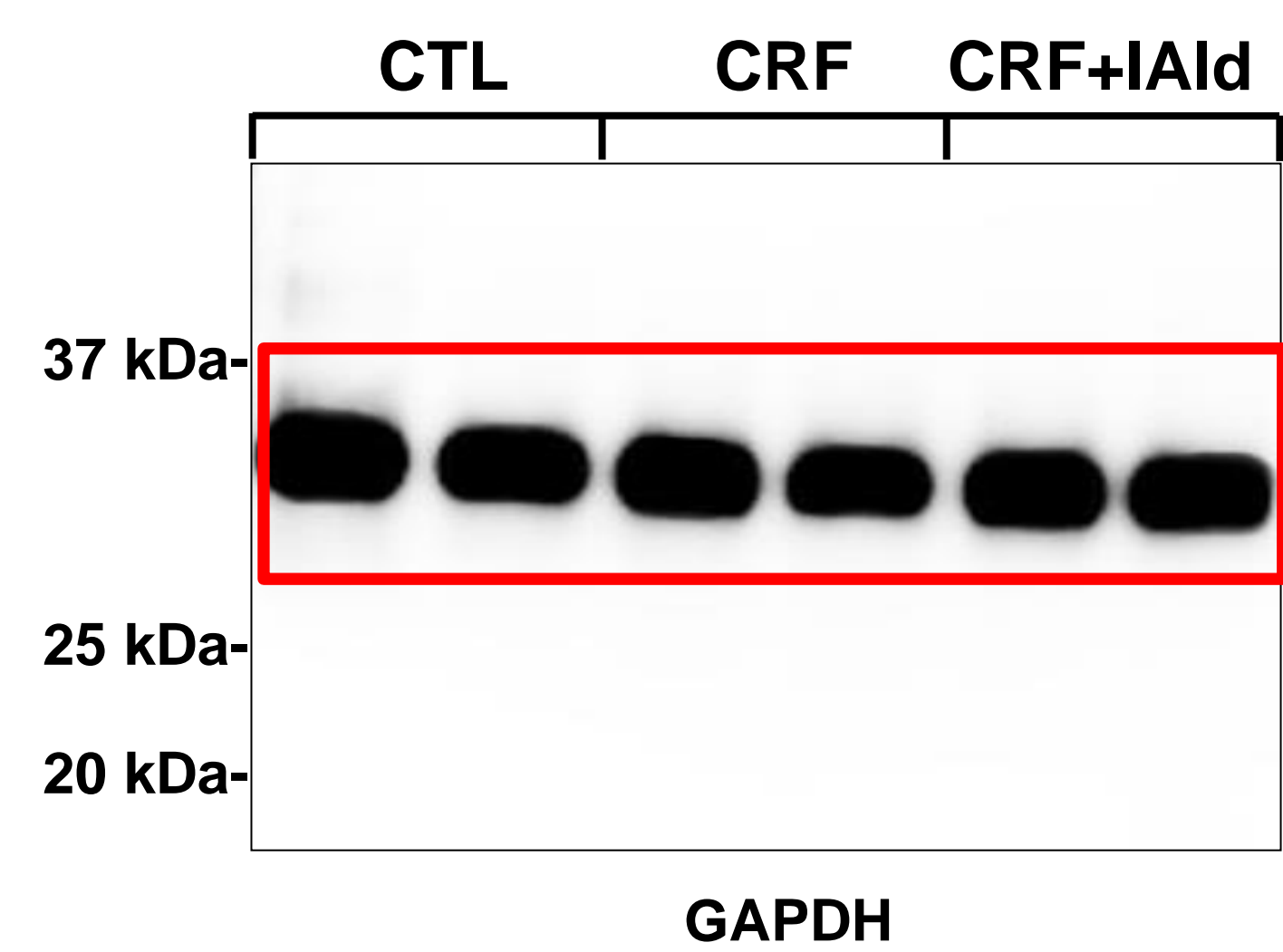

Supplement: Supplementary file 2 — Original and and uncropped Western blots [file 41392_2024_1913_MOESM2_ESM.pdf]
